# A mutation in the c-Fos gene associated with congenital generalized lipodystrophy

**DOI:** 10.1186/1750-1172-8-119

**Published:** 2013-08-07

**Authors:** Birgit Knebel, Jorg Kotzka, Stefan Lehr, Sonja Hartwig, Haluk Avci, Sylvia Jacob, Ulrike Nitzgen, Martina Schiller, Winfried März, Michael M Hoffmann, Eva Seemanova, Jutta Haas, Dirk Muller-Wieland

**Affiliations:** 1 Institute of Clinical Biochemistry and Pathobiochemistry, German Diabetes Center at the Heinrich-Heine-University Duesseldorf, Leibniz Center for Diabetes Research, Duesseldorf, Germany; 2 2nd Clinical Institute of Medical and Chemical Laboratory Diagnostics, Medical University of Graz, Graz, Austria; 3 Synlab Centre of Laboratory Diagnostics Heidelberg, Heidelberg, Germany; 4 Division of Clinical Chemistry, University Medical Center, Freiburg, Germany; 5 Department of Medicine, University Medical Center, Freiburg, Germany; 6 Department of Clinical Genetics, Institute of Biology, and Medical Genetics, 2nd Medical School, Charles University, Prague, Czech Republic; 7 Institute for Diabetes Research, Department of General Internal Medicine, Asklepios Clinic St. Georg, Asklepios Campus Hamburg, Medical Faculty of Semmelweis University, Hamburg, Germany

**Keywords:** Congenital lipodystrophy, Immediate early genes, Protein/DNA interaction, Transcriptional regulation

## Abstract

**Background:**

Congenital generalized lipodystrophy (CGL) or Berardinelli–Seip congenital lipodystrophy (BSCL) is a rare genetic syndrome characterized by the absence of adipose tissue. As CGL is thought to be related to malfunctions in adipocyte development, genes involved in the mechanisms of adipocyte biology and maintenance or differentiation of adipocytes, especially transcription factors are candidates. Several genes (BSCL1-4) were found to be associated to the syndrome but not all CGL patients carry mutations in these genes.

**Methods and results:**

In a patient with CGL and insulin resistance we investigated the known candidate genes but the patient did not carry a relevant mutation. Analyses of the insulin activated signal transduction pathways in isolated fibroblasts of the patient revealed a postreceptor defect altering expression of the immediate early gene c-fos. Sequence analyses revealed a novel homozygous point mutation (c.–439, T→A) in the patients’ c-fos promoter. The point mutation was located upstream of the well characterized promoter elements in a region with no homology to any known cis-elements. The identified mutation was not detected in a total of n=319 non lipodystrophic probands. *In vitro* analyses revealed that the mutation facilitates the formation of a novel and specific protein/DNA complex. Using mass spectrometry we identified the proteins of this novel complex. Cellular investigations demonstrate that the wild type c-fos promoter can reconstitute the signaling defect in the patient, excluding further upstream signaling alterations, and *vice versa* the investigations with the c-fos promoter containing the identified mutation generally reduce basal and inducible c-fos transcription activity. As a consequence of the identified point mutation gene expression including c-Fos targeted genes is significantly altered, shown exemplified in cells of the patient.

**Conclusion:**

The immediate-early gene c-fos is one essential transcription factor to initiate adipocyte differentiation. According to the role of c-fos in adipocyte differentiation our findings of a mutation that initiates a repression mechanism at c-fos promoter features the hypothesis that diminished c-fos expression might play a role in CGL by interfering with adipocyte development.

## Introduction

Lipodystrophy can be acquired or inherited and results in partially or complete loss of adipose tissue. In the most severe form, congenital generalized lipodystrophy (CGL) or Berardinelli–Seip congenital lipodystrophy (BSCL), total absence of adipose tissue is associated with altered development, fatty liver, muscular hypertrophy, hypertriglyceridemia, acanthosis nigricans, hyperinsulinism and type-2-diabetes [1,2].

CGL is rare with estimated 1:10 million births and thought to be a genetic syndrome with autosomal recessive trait [2]. In humans several candidate genes (BSCL1-4) were found to be associated to the syndrome [1,3]. BSCL1/AGPAT2 and BSCL2/seipin are identified in the majority of CGL patients. In single families BSCL3/caveolin-1 and BSCL4/PTRF-Cavin were identified. The BSCL genes are part of mechanisms involved in the adipocyte formation and growth including lipid droplet formation vesicle transport or and glycerophospholipid synthesis [1]. Thus CGL have been valuable models for the identification of new genetic loci involved in development, distribution and plasticity of white fat cells. Although patients are rare and an estimated 1 of 4 existing cases has been included into studies, not all individuals identified bare mutations in these target genes [1].

Lipodystrophy resemble syndromes of disturbed adipocyte biology or metabolism but the severe congenital forms are thought to be related to malfunctions in adipocyte development [4,5]. Therefore genes involved in the differentiation of adipocytes, especially transcription factors, are hot candidates. Prominent examples are PPARγ, SREBP-1c in HIV therapy and SREBP-1c or C/EBP in mouse models causing lipodystrophy [2,4-8]. Another potential candidate is the transcription factor c-Fos a member of the AP-1 complex that is essential to initiate adipocyte differentiation. Accompanied with peak c-fos gene expression a sequential gene expression cascade of specific transcription factors necessary in adipocyte development is temporarily initiated leading to fully differentiated adipocytes [9,10]. C-fos has been proven to be essential in this transcriptional activation and knockdown of c-fos abolished the ongoing differentiation process [10].

Since a mutation in the known BSCL genes have not been found a patient with CGL and insulin resistance we examined insulin signaling as central metabolic signal transduction pathways and show the identification of a homozygous point mutation in the c-fos promoter (c.–439, T→A) in this patient. This mutation causes a novel protein/DNA complex which ubiquitously lowers basal and inducible c-fos promoter activity. According to the role of c-fos in adipocyte differentiation our investigations provoke the hypothesis that diminished c-fos expression interferes with adipocyte development and might play a role in CGL.

## Methods

### Cell culture

Fibroblasts initiated from skin biopsies of patient and healthy caucasian volunteer controls were expanded for 4 cycles and stored in liquid nitrogen. Cells were recultured (DMEM, 10% FCS; Life Technologies, Darmstadt, Germany) and expanded for a maximum of 3 passages before harvesting. For exogenous stimulation fibroblasts were grown to 70% confluence and serum starved (1% FCS) for 40 h (quiescent fibroblasts indicated as basal in figure legends) prior to induction with 10^-7^ M insulin, 10^-8^ M IGF-1, 1.5×10^-8^ EGF or 3.3×10^-9^ M PDGF. Preadipocytes (3T3-L1) and muscle cells (A7r5) were cultured in DMEM and liver cells (HepG2) in RPMI 1640 (Life Technologies) supplemented with 10% FCS. Institutional research ethics approval (PV3641) in line with the Helsinki Declaration was obtained for this study.

### Nuclear extracts

Nuclear extracts from fibroblasts of the patient and controls, or HepG2 cells were prepared as described [11].

### Insulin induced signal transduction

MAPK activity assays and western blotting with polyclonal anti-Akt or anti-phospho Akt antibody (New England Biolabs, Frankfurt, Germany) or polyclonal anti-MAPK antibody (BD Transduction; Heidelberg, Germany) were performed as described [11].

### Real-time (RT) PCR

Total RNA was extracted with RNeasy Mini Spin Kit (Qiagen, Hilden, Germany). RT- PCR analyses were performed in triplicates with c-fos gene-specific probes and 18S RNA internal standard (Applied Biosystems, Darmstadt, Germany) as described [12]. Expression results were determined as relative RNA amounts of target.

### Plasmid constructs for transient transfection of primary fibroblasts

C-fos promoter (nt −734 to +43; numbering based on TS (=1) according to K00650) was PCR amplified from genomic DNA of controls and patient using following primers: -734 to −712: 5’-GCGAGGAACAGTGCTAGTATTGCT-3’/ +43 to +12: 5’-CGGCTCAGTCTTGGCTTCTCAGTTGCTCGCT-3’. Wild type c-fos promoter fragment and the corresponding fragment of the patient were inserted in sense orientation into pGL3basic vector (Promega, Mannheim, Germany) to bring the reporter gene luciferase under control of wild type c-fos promoter (pc-fos-wt) or the mutated promoter (pc-fos -439T>A). The expression vector pFA-Elk-1 containing the regulative domains of transcription factor Elk-1 (aa 307 to 427) fused to the heterologous DNA binding domains of Gal4 (aa 1 to 147) under control of a MLV-promoter was used (Life Technologies). For monitoring transactivation the reporter plasmid pGal4-Luc5 containing the luciferase gene under control of 5× Gal4 binding elements was cotransfected in these experiments (Life Technologies). As reference of transfection efficiency the β-galactosidase expression vector pEF-ßGal was used. Independent plasmid preparations and cells were used for replicate experiments.

### Transfection

Cell suspensions (2×10^5^ cells/well) of 3T3-L1, A7r5, HepG2 cells or fibroblasts of patient and control were mixed with vectors as indicated in figure legends and pulsed for 18 msec (3T3-L1, A7r5, HepG2) or 9 msec (fibroblasts). For exogenous induction, cells were serum-starved on day one following transfection for 40 h and incubated with 10^-7^M insulin or 3.3×10^-9^M PDGF for 3 h before harvesting. Transfection, monitoring of transfection efficiency and luciferase assays were performed as described [11].

### Direct sequence analyses

Genomic DNA was extracted from fibroblasts of patient or control using the Qiagen blood kit™. For reevaluation of the identified mutation only limited amounts of genomic DNA of the patient’s parents but no biopsies were available. The coding sequence of AGPAT2 (NM_006412.3), caveolin-1 (NM_001753.4; NM_001172895.1), seipin (NM_001122955.3), CAV/PTFR (NM_012232) and c-fos promoter (K00650) were analyzed by direct sequencing (ABI PRISM 3100, Applied Biosystems).

### Restriction analyses for the identified c-fos promoter mutation

Genomic DNA of 319 unrelated caucasian subjects was isolated from PMBCs and c-fos promoter (−603 to +12) was amplified (−603 to −579: 5’ primer: 5’-AGGCTTAAGTCCTCGGGGTCCTGT-3’; +43 to +12: 3’ primer: 5’-CGGCTCAGTCTTGGCTTCTCAGTTGCTCGCT-3’). PCR products were reamplified with a mutated primer (−441: C>*A)* introducing a Tsp509I restriction site solely in wild type c-fos promoter (5’ primer: -470 to −440, c-fos mut −441: C>A: 5’-CATTGAACCAGGTGCGAATGTTCTCTCT*A*A-3‘ and 3‘ primer −337 to −307: 5’-AGATGTCCTAATATGGACATCCTGTGTAAG-3’). PCR products were Tsp509I digested and size fractionated. Genomic DNA of patient was always treated in parallel for control. 10% of samples were randomly chosen to confirm results by direct sequencing.

### DNaseI protection analyses

Footprinting analyses were performed using the sure track footprinting kit™ (GE Healthcare, Munich, Germany). Promoter fragments (nt −462 to −325) were isolated from pc-fos-wt or pc-fos-patient by EcoRI/EcoNI restriction and cohesive ends were labeled with 20 μCi [α^32^P] dATP and Klenow fragment. 100.000 cpm of radiolabeled fragments were incubated with increasing concentrations of nuclear extracts (4 μg, 8 μg, 20 μg, 40 μg) from human liver cells (HepG2) and subsequently digested for 1 min with different concentrations of DNaseI (0.11U, 0.33U, 1.0U). Reactions were terminated and deproteinized by LiCl precipitation. DNA fragments were precipitated and separated on an 8% PAGE containing 7 M urea. Purine nucleotide sequence ladders from the EcoRI/EcoNI promoter fragments were loaded in parallel to confirm sequence of protected areas.

### Electrophoretic mobility shift assay (EMSA)

EMSA were performed according to [13] and reactions were analyzed on 5% non-denaturating PAGE. For analyses of the *in vitro* ternary complex formation with nuclear extracts of fibroblasts from control or the patient, 2 pmol s*re*-element promoter fragments (−331 to −280 5’CCCCTTACACGGATGTCCATATTAGGACATCTGCGTCAGCAGGTTTCCACG; 3’GGAATGTGCCTACAGGTAATAATCCTGTAGACGCAGTCGTCCAAAGGTGCCC) were labeled with 20 μCi [α32P]dGTP using 5U Klenow fragment, prior to use. For completion experiments 100- or 10-fold molar excess of unlabeled *sre* fragments or unspecific SP-1 promoter fragment (5’-GTTAGGGGCGGGATGGGCGGAGTT -3’) were used.

Analyses of the novel protein/DNA interaction at the c-fos promoter were performed with c-fos-wt (−451 to −431: 5’-TGTTCTCTCTCATTCTGCGCCG-3’) or c-fos-patient (−451 to −431, -439T>A: 5’-TGTTCTCTCTCA*A*TCTGCGCCG-3’) endlabeled with 5U PNK and 20 μCi [γ^32^P]dATP, prior to use. For competition experiments 10× or 100× unlabeled c-fos-wt or pc-fos-patient fragment were used. Experiments were performed with nuclear extracts (5 μg) of human liver cells (HepG2).

### Protein identification of protein/DNA complex proteins by MALDI-MS

For preparative EMSA a Cy3-labeled c-fos-patient fragment was used in the procedure. Gels were scanned using a Typhoon scanner (GE, Freiburg, Germany) and fluorescence marked bands were cut from gels. The gel slices were placed on a 10% SDS-PAGE for separation of complex proteins. Four independent EMSA replicates were performed and cutted protein/DNA complex were separated on four SDS-PAGE each. Of all protein bands three different punch samples were excised and subjected to mass spectrometry analyses according to [14] for identification. Acquired mass spectra (peptide mass fingerprint) were automatically calibrated and annotated using Compass 1.3 software and xml formatted peak lists were transferred to Proteinscape3.0 (Bruker Daltonik, Bremen Germany). MS peptide mass fingerprint were used to search a human sub-set of Swiss-Prot (Sprot_2011; 20249 20401 protein entries) non-redundant database using Mascot search engine (Version 2.2, Matrix Science Ltd, London, UK) Mass tolerance was set to 50 ppm for peptide spectra and a combined mascot score over 70 was taken significant (p < 0.01). For verifying the results each protein spot was picked and identified from at least three physically different gels.

### Affymetrix chip expression analyses: identification of differentially regulated transcripts independent to individual expression variation

Fibroblasts of patient and 6 individual controls were cultured to passage 6 each. Four replicate analyses of patient cells (initiated from two primary stored cell pools) and 6 individual controls (initiated from one primary stored cell pool each) were used. Equal amounts of total RNA were processed according to the GeneChip One-Cycle eukaryotic Target Labeling Assay (GeneChip Expression analysis technical Manual, http://www.affymetrix.com/support/Technical/manual/expression_manual.affx) and used for expression analyses with Hu95A_v2 Arrays (Affymetrix UK Ltd). Syntheses of cRNA and fragmentation were quality controlled and monitored with a RNA 6000 nano kit (Agilent, Taufkirchen, Germany). Detection of probe sets was performed using a GeneChip scanner (GCOS 1.4 package, Affymetrix). The original CEL files were directly implemented into Genespring 12.0 (Agilent) for analyses. The Genespring 12.0 Volcano Plot analyses workflow with default settings (paired t-test, multiple testing correction: Benjamini-Hochberg) of the gene expression data sets were used to identify genes with statistic significant expression (p < 0.05) and a minimum 1.5-fold difference among conditions. Full data sets are available under accession number GSE39825 (http://www.ncbi.nlm.nih.gov/geo/).

### Web based functional annotation of differentially expressed genes and identified proteins

For functional annotation and conserved promoter element site search web based tools were used (http://david.abcc.ncifcrf.gov/) [15,16]. For functional annotation protein IDs or Affymetrix IDs, fold change, and t-test p-value of detection significance were imported to Ingenuity Pathway Analysis (IPA) System (http://www.ingenuity.com). IPA was carried out with p < 0.002 as cutoff point. Pathways indicating altered transcriptional regulation were deduced from fold change differences observed.

### Statistical analyses

Data are given as means ± S.D. Students t-test was used to determine statistical significance.

## Results

### Patient characteristic and genetics

The female caucasian patient was born at term with reduced birth weight (2,950 g). The parents were healthy, not consanguineous and gave birth to four further healthy children. At age of one year the patient retrieved to thrive and beginning lipodystrophy was diagnosed. Pronounced *acanthosis nigricans* was observed, being a hint for altered insulin signaling. Until the age of five years prediabetes, progressive hepatomegaly and lipoatrophy appeared with complete loss of adipose tissue. Physical examination revealed generalized decreased subcutaneous adipose tissue, distended abdomen with enlarged palpable liver and growth retardation from 10% (1 year) to 75% (7 years) of normal range. The patient had the typical appearance of congenital generalized lipodystrophy including hypertrichiosis, hepatomegaly, splenomegaly, but no mental retardation. The patient had marked muscularity probably due to missing subcutaneous adipose tissue. The main known candidate genes associated with congenital generalized lipodystrophy, i.e. BSCL1/AGPAT2, BSCL2/seipin, BSCL3/caveolin-1 and BSCL4/PRTF were analyzed. No sequence alteration specific for the lipodystrophic phenotype was identified (data not shown). At age of five years laboratory analyses showed elevated plasma cholesterol levels (450 mg/dl) and modestly elevated triglycerides (218 mg/dl). Glucose intolerance detected by oGTT showed an increase of blood glucose levels from 103 mg/dl (normal range 65 to 100 mg/dl) up to 176 mg/dl (normal range 80 to 126 mg/dl) and plasma insulin levels from 96 mU/l to 276 mU/l, respectively, indicating insulin resistance. The patient died at the age of eight during a hyper acute varicella infection as primary cause of death. An autopsy was not performed.

### Insulin mediated transcriptionally activation of the c-fos gene

As the patient was the only known case of lipodystrophy in the family nothing remained but analyzing a possible defect in insulin signaling, we characterized known signaling pathways. For this purpose we utilized primary fibroblasts initiated from skin biopsies.

**Figure 1:**
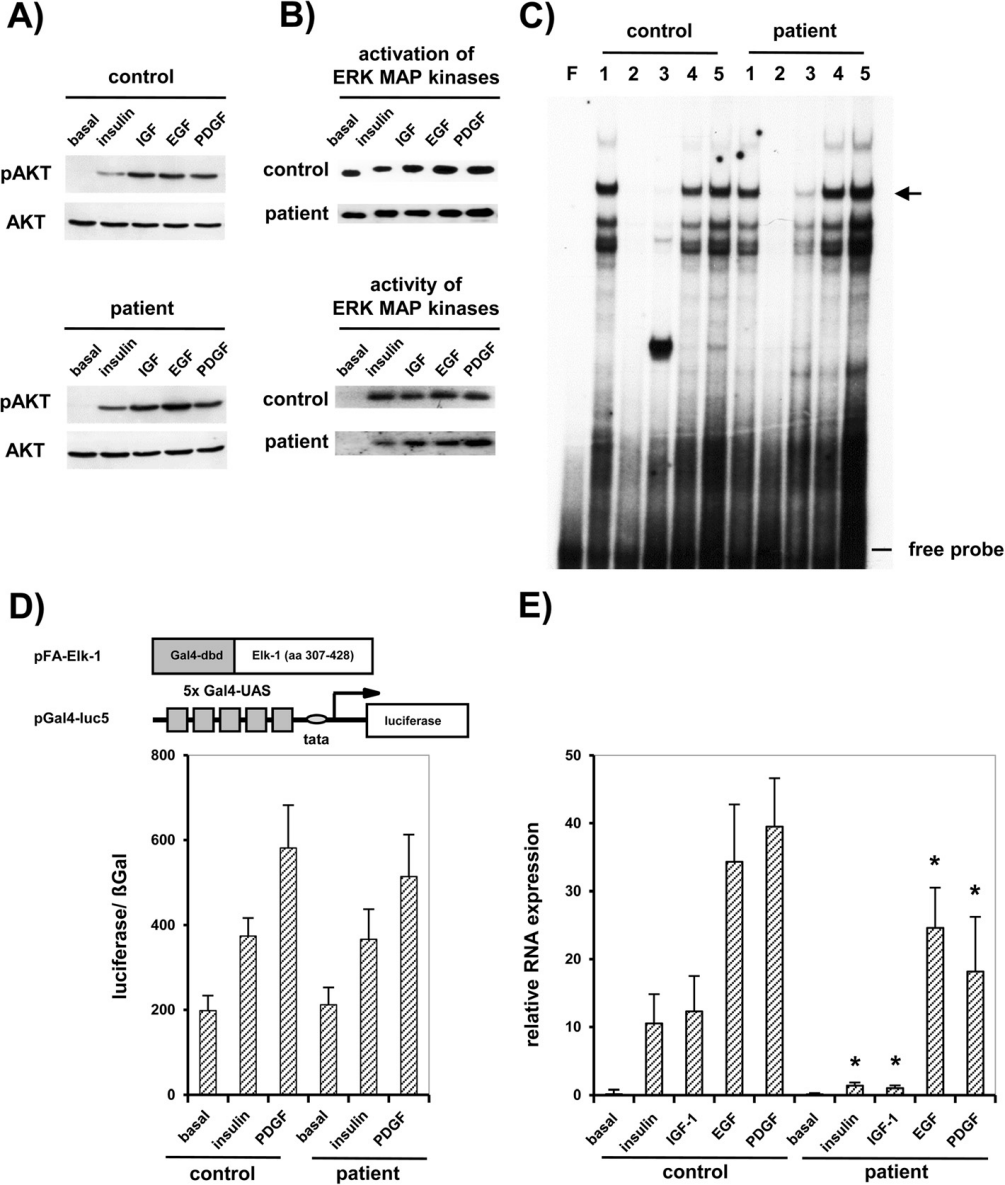
**Localization of the postreceptor defect to c-fos expression. A)** Western blot analyses of Akt phosphorylation and abundance. **B)** Phosphorylation of MAPK was assayed by western blot analyses. Activity of MAPK was detected in in-gel kinase assays. **C)** Formation of the ternary complex at *sre* element of c-fos promoter in control and patient. The specific complex is indicated by an arrow (lane F: free probe, 1: no competition; competition: 2: 100x SRE, 3: 10x SRE, 4: 100x SP-1, 5: 10x SP-1). **D)** Activation of ternary complex factor Elk-1 following insulin and PDGF stimulation in patient and control cells. Results are given as means (±S.D.). *p< 0.05 *vs* basal control. **E)** Transcriptional activation of c-fos mRNA. The mRNA levels were normalized against 18S rRNA as internal control. Values are means (±S.D.) from four independent experiments, each performed in triplicate. *p< 0.05 *vs* basal control.

The *in vitro* insulin receptor binding capacity, auto- and substrate-phosphorylation was in normal range (data not shown). The insulin mediated signaling cascade including Akt abundance or phosphorylation (Figure 1A) and ERK1/2-MAPK activation or activity (Figure 1B) was comparable to controls in patient cells, indicating no disturbance in these pathways. A definite endpoint of MAPK cascades is the transcriptional activation of the immediate-early genes c-fos [17], whereas the c-fos gene activity is directly related to formation of a ternary transcription activation complex at the *sre*-element and the phosphorylation of the ternary complex factor Elk-1 by ERK1/2-MAPK [18]. Investigations showed that the formation of the ternary complex (Figure 1C) and Elk-1-activation dependent transcription was not altered in cells of the patient (Figure 1D). Nevertheless in patient cells induction of c-fos mRNA expression was nearly completely lost following insulin and IGF-1 induction and clearly reduced following PDGF and EGF induction in contrast to control cells (Figure 1E). These findings focused the defect to c-fos gene directly.

### Identification of a point mutation in c-fos promoter causing a novel specific protein/DNA interaction

**Figure 2:**
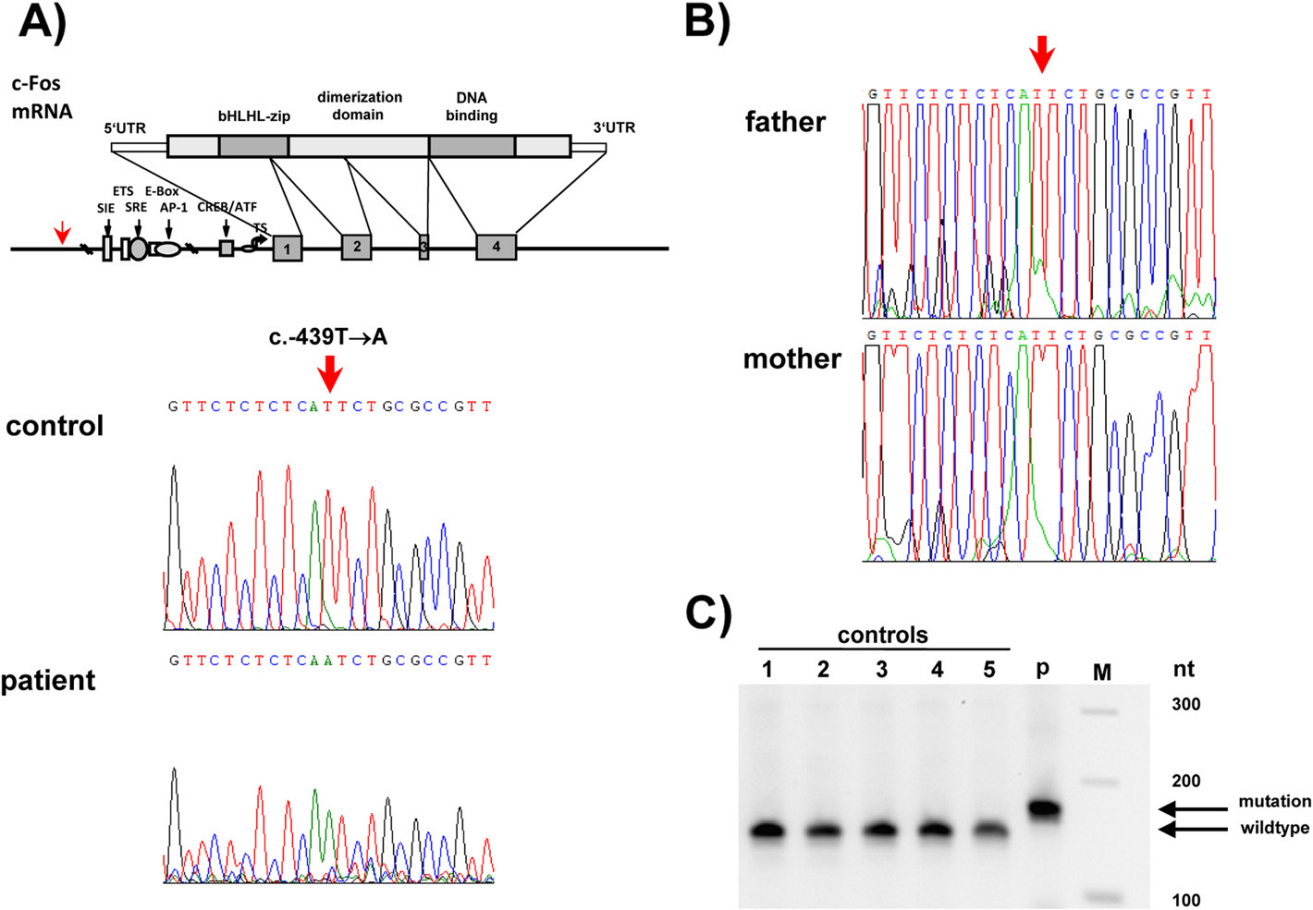
**Identification and impact of a homozygous c-fos promoter point mutation in the patient. A)** A homozygous point mutation in c-fos promoter (T → A) at position c.-439 was identified in the patient. **B)** The mutation was not identified in the patient’s father or mother or **C)** in a restriction based assay with 319 control subjects. Representative samples are shown (lane 1–5; p: patient; M: size standard).

Sequence analyses of the patients’ c-fos gene identified next to a common heterozygous SNP in 5’UTR (rs7101; c.–60) a novel homozygous point mutation in the promoter at position c.–439 (Figure 2A) upstream of well characterized regulatory promoter elements. This mutation was not identified in 319 control subjects or in the confirmed parents of the patient, indicating a *de novo* mutation (Figure 2B).

**Figure 3:**
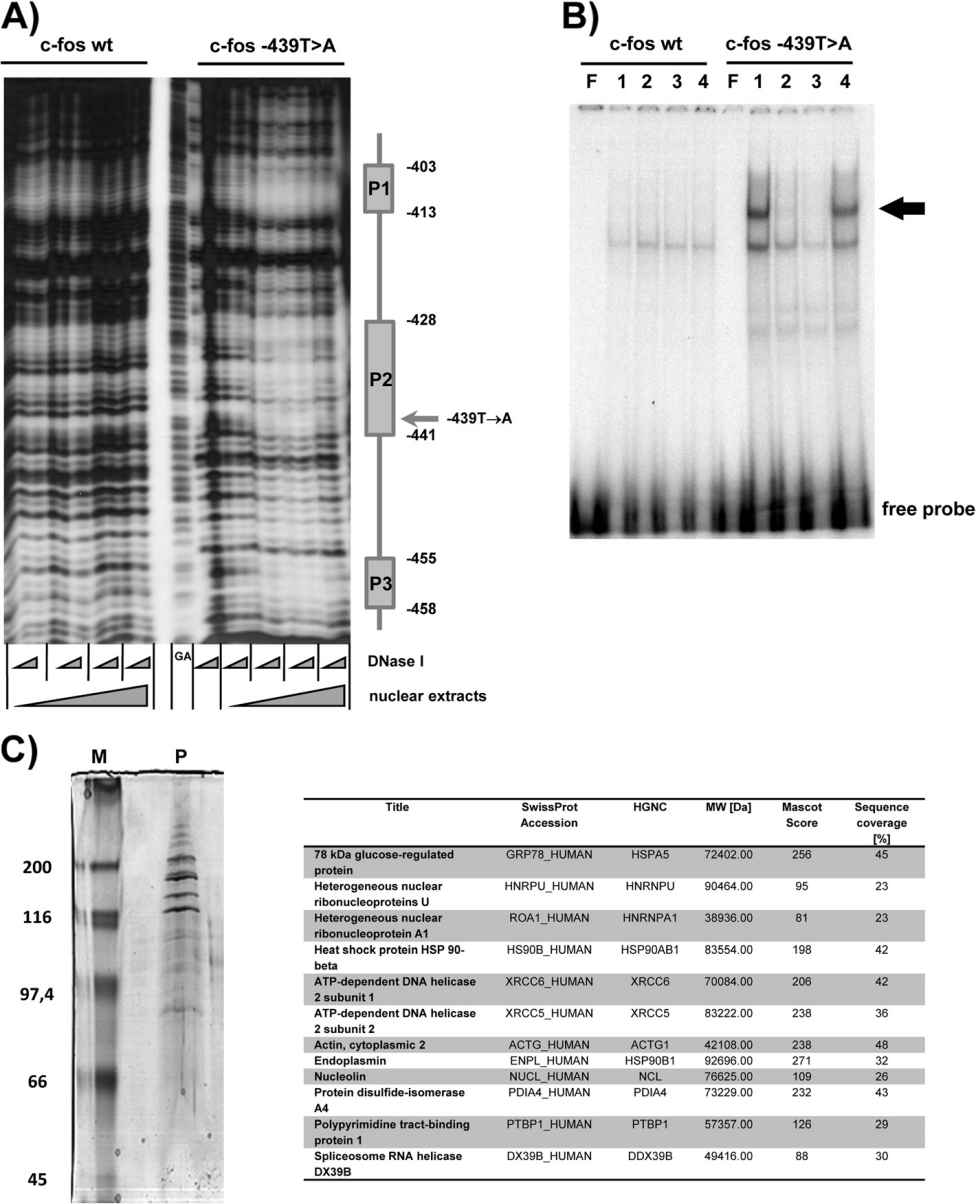
**Identification and characterization of a novel protein/DNA complex forming specifically at the mutation identified in the patient. A)** Protein binding to c-fos promoter and mapping of nucleotides necessary for complex formation. Radiolabeled c-fos-wt or mutated pc-fos-c.–439 T→A DNA fragments were subjected to DNaseI protection assay with increasing amounts (◄: 4 μg, 8 μg, 20 μg, 40 μg) of liver cells (HepG2) nuclear protein extracts and digested with varying DNaseI concentrations (◄: 0.11U, 0.33U to 1.0U). A protein/DNA interaction does solely occur with c-fos–c.-439T>A. The sequence of protected areas P1 (nt −413 to −403 (CCCAGCCGCGG) P2 (nt −441 to −428 (CA*A*TCTGCGCCGTT) and P3 (nt −458 to −455 (GTGC) indicated the mutation being located in P2. A typical result from 5 experiments is shown (lane GA: purine sequence ladder). **B)** EMSA with nuclear protein extracts from liver cells (HepG2) using c-fos-wt (−451 to −430) or mutated pc-fos-c.–439 T→A as probe. The specific complex is indicated by an arrow. Mutation specific complex formation was tested by cross competition with 100-fold excess of non radiolabeled fragments of either c-fos-wt or pc-fos-c.–439 T→A (lane F. free probe, 1: no competition; competition: 2: 50x, 3: 100x specific competitor, 4: 100x cross competition) **C)** Size fractionation of EMSA protein band on denaturing SDS PAGE. All resulting protein bands were subjected to mass spectrometry. Acquired data from each individual spot were used to search a human sub-set of Swiss-Prot (Sprot_2011; 20249 protein entries) for protein identification.

One functional possibility of a point mutation in a promoter is the direct interference with protein/DNA interactions. Utilizing the c-fos promoter in nuclease protection assays revealed novel protein binding sites (P1-P3) only with DNA-fragments bearing the identified point mutation in P2 (Figure 3A). EMSA confirmed that this specific protein/DNA interaction only occurs if the point mutation was present (Figure 3B). A mass spectrometry approach revealed that the novel formed protein/DNA complex consisted of at least 13 proteins (Figure 3C). Database analyses showed that identified proteins were not classical transcription factors, but nucleases, helicases or structural proteins (Table 1).

**Table 1:** Functional annotation of proteins identified in the novel DNA binding complex

**Category**	**Term**	**EASEScore**	**Benjamini**	**Fisher exact**	**Fold enrichment**
**GOTERM_MF**	Nucleotide binding	2.20E-07	6.80E-06	4.10E-08	5.3
**GOTERM_MF**	RNA binding	1.80E-04	3.70E-03	1.90E-05	9
**GOTERM_MF**	Structure-specific DNA binding	2.10E-04	3.30E-03	6.90E-06	29.8
**GOTERM_MF**	ATP binding	6.00E-04	7.40E-03	1.10E-04	5.1
**GOTERM_MF**	Adenyl ribonucleotide binding	6.40E-04	6.60E-03	1.30E-04	5.1
**GOTERM_MF**	Adenyl nucleotide binding	8.50E-04	7.50E-03	1.70E-04	4.8
**GOTERM_MF**	Ribonucleotide binding	1.90E-03	1.20E-02	4.60E-04	4.1
**GOTERM_MF**	Purine nucleotide binding	2.40E-03	1.40E-02	6.10E-04	3.9
**GOTERM_MF**	ATP-dependent helicase activity	3.00E-03	1.50E-02	8.70E-05	33.1
**GOTERM_MF**	Purine NTP-dependent helicase activity	3.00E-03	1.50E-02	8.70E-05	33.1
**GOTERM_MF**	Double-stranded telomeric DNA binding	4.20E-03	1.90E-02	7.80E-06	432.8
**GOTERM_MF**	Protein C-terminus binding	6.00E-03	2.30E-02	2.60E-04	23
**GOTERM_MF**	Sequence-specific DNA binding	1.30E-02	4.50E-02	1.70E-03	7.1
**GOTERM_MF**	ATP-dependent DNA helicase activity	2.10E-02	7.00E-02	2.30E-04	86.6
**GOTERM_MF**	Single-stranded RNA binding	2.30E-02	6.90E-02	2.70E-04	80.1
**GOTERM_MF**	ATPase activity	3.10E-02	8.90E-02	3.10E-03	9.7
**GOTERM_MF**	DNA helicase activity	3.30E-02	9.10E-02	6.00E-04	54.1
**GOTERM_MF**	Single-stranded DNA binding	4.60E-02	1.20E-01	1.10E-03	39.3
**GOTERM_MF**	DNA-dependent ATPase activity	4.70E-02	1.20E-01	1.20E-03	38
**GOTERM_MF**	Promoter binding	4.70E-02	1.20E-01	1.20E-03	38
**GOTERM_MF**	Double-stranded DNA binding	7.90E-02	1.90E-01	3.50E-03	22.3

For functional annotation of proteins identified and listed in Figure 3C web based tools as http://david.abcc.ncifcrf.gov/ were used. Within these analyses proteins were classified according to their molecular function given in the column “term”. The data sets were analyzed with the categorical over-representation function of EASE (upper bound of the distribution of Jacknife Fisher exact probabilities). Fold enrichment indicates the enrichment of term in search string.

### The point mutation c.–439 T→A in the 5’UTR of c-fos gene results in ubiquitous impairment of c-fos promoter activity

**Figure 4:**
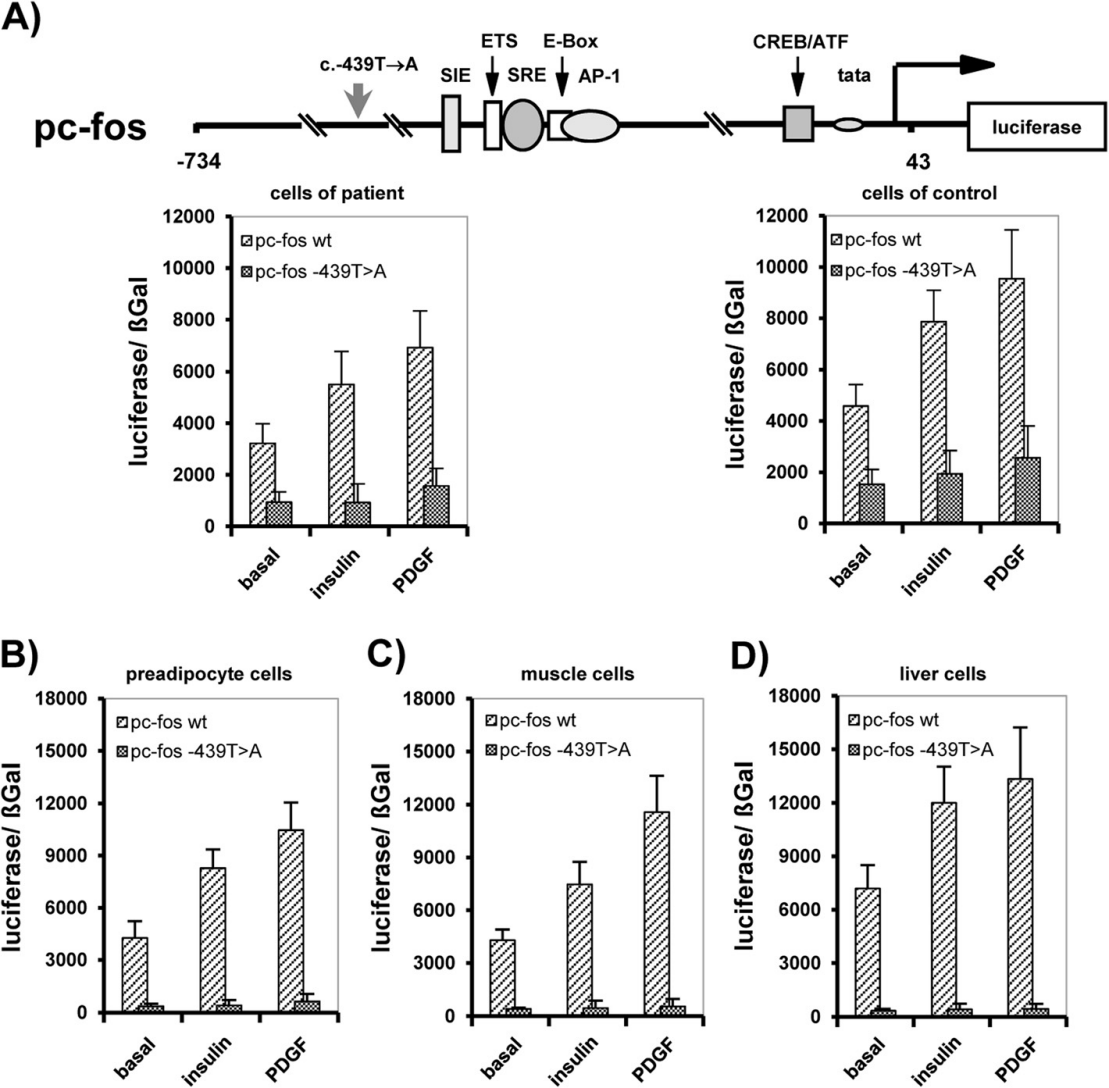
**Effect of the identified homozygous c-fos promoter point mutation on c-fos transcription. A)** Basal and inducible c-fos promoter activity is dependent on wt c-fos promoter and abrogated by mutated c-fos promoter (pc-fos-c.–439 T→A) in patient and control cells. Data of replicate promoter reporter analyses (n=6) are given as mean (±S.D.; p< 0.05). General transcriptional impairment due to c-fos promoter (pc-fos-c.–439 T→A) mutation in **B)** preadipocytes (3T3L1), **C)** muscle cells (A7r5) and **D)** liver cells (HepG2). Data of promoter reporter analyses are given as mean of replicate experiments (n=6) (±S.D; p<0.05).

To test the regulatory relevance of identified mutation in the c-fos promoter we performed promoter reporter gene analyses. Transfecting a wild type c-fos promoter into the patient’s cells revealed that the basal and inducible promoter activity is completely reconstituted (Figure 4A). *Vice versa* transfecting a c-fos promoter bearing the identified mutation into control cells revealed that the observed diminished expression and inducibility of c-fos was dependent from cellular environment (Figure 4A). These data exclude further proximal signaling defects and support a defect intrinsic to the c-fos promoter as the mutation identified. Further analyses demonstrated that this observation was not cell specific as in preadipocytes, muscle and liver cells c-fos promoter activity and activation was evenly inhibited by the point mutation (Figure 4B-D).

### Biological relevance of reduced basal and inducible c-fos expression

**Figure 5:**
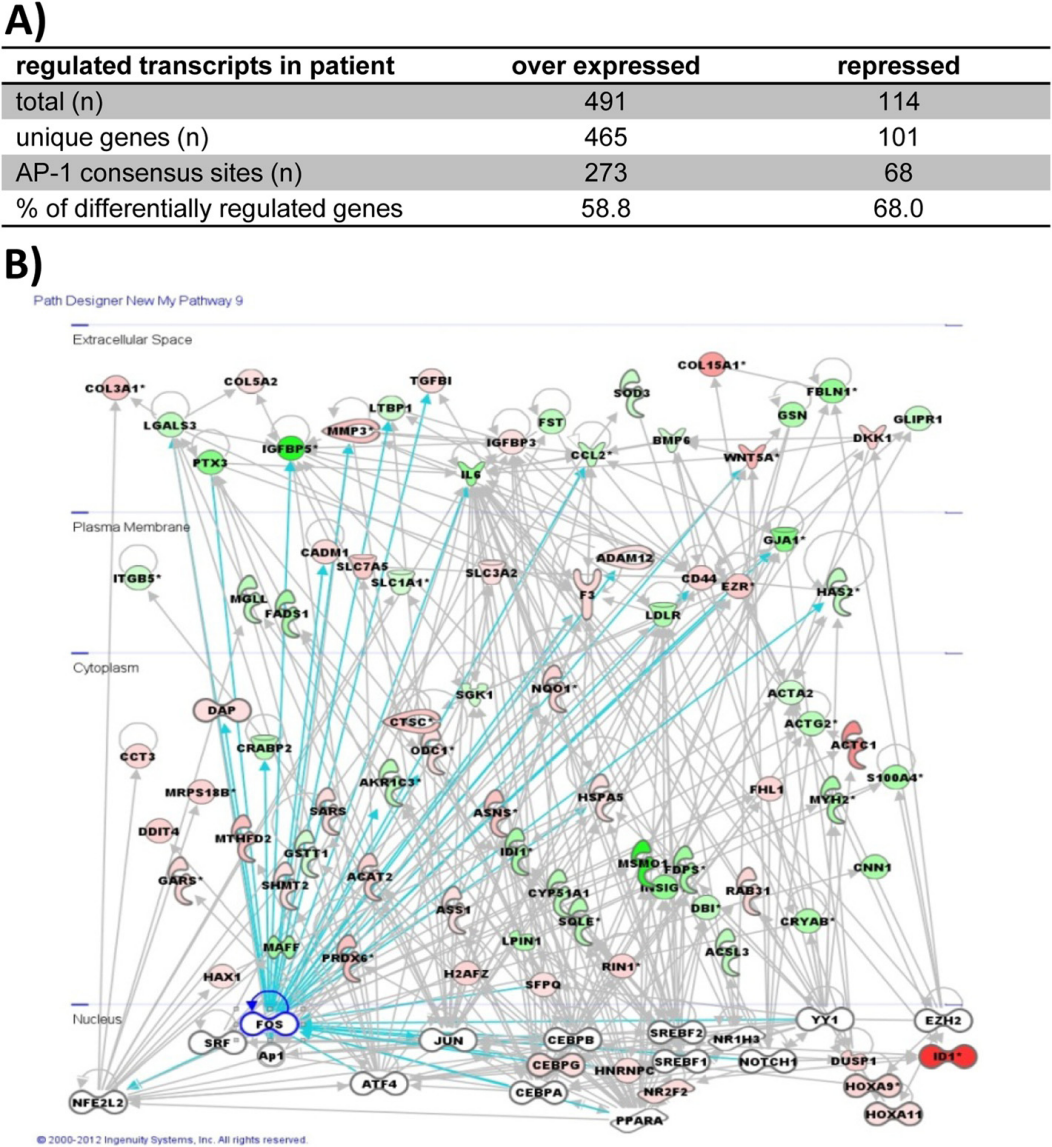
**Gene expression alterations due to diminished c-fos expression. A)** Gene expression date were analyzed for statistically significant different gene expression (1.5-fold difference; p<0.05). Resulting transcript IDs were subjected to automated annotation for conserved AP-1 transcription factor binding sites. **B)** The pathways showing altered transcriptional regulation were deduced from differential gene expression identified. Colored genes (red and green) were identified differentially regulated by microarray analysis. Genes directly regulated by c-fos are indicated (blue line). Predicted transcription factors (white) involved in observed gene regulation were deduced using Ingenuity Pathway Analysis ((IPA System (http://www.ingenuity.com).

To test if the mutation and novel protein/DNA complex observed has an impact in cellular context, we performed a gene expression survey in the patient (Additional file 1: Table S1). Functional analyses of these results revealed that multiple metabolic or transcriptional processes and a large fraction of genes involved in cell cycle control and differentiation were affected (Additional file 2: Table S2). Data base analyses suggest that most of differentially regulated transcripts bear the c-fos responsive AP-1 promoter consensus sequence (Figure 5A). Further *in silico* analyses indicate that next to c-fos and further AP-1 complex factors, differentially expressed transcripts in the patient are also regulated by transcription factors necessary in adipocyte differentiation and maturation i.e. ATF4, PPARα, SREBP-1or-2 and CEBP family members (Figure 5B).

## Discussion

### Implication of the mutation (c.–439 T→A) in the promoter of c-fos gene

Congenital lipodystrophy is characterized by the complete loss of adipose tissue. In our patient with CGL we identified a homozygous *de novo* point mutation in the c-fos promoter. Although this is an unexpected finding, increasing genomic information indicated that approximately 50–100 germ line *de novo* mutations can occur in each individual [19]. Nevertheless, the mutation identified in this study has a clear functional relevance of c-fos promoter activity. Due to the identified mutation a novel mutation-specific protein/DNA interaction is formed. As consequence basal and inducible c-fos expression is ubiquitously lowered. The proteins identified in the complex represent no classical DNA interacting proteins but DNA modifying helicases, nuclear ribonucleoproteins (hnRP), structural proteins, and heat shock proteins. It was shown that the single strand binding ATP dependent helicases are involved in telomere maintenance, double strand breaking repair and DNA unwinding as needed to transcriptional activation, especially it has long been noted that UV initiated DNA damage interferes with inducibility of c-fos expression and UV induced DNA damage interferes with adipocyte differentiation [20,21]. HnRPs localized to the nucleus are involved in transcriptional repression processes including c-fos mRNA transcription [22]. Furthermore nuclear actin or nucleolin are involved in chromatin-remodeling processes and actin can be associated to hnRNPs A/B type family as also shown here [23,24]. Such modifications in chromatin structure are required for gene expression and adipocyte differentiation as local chromatin remodeling has been described in the regulation of the PPARγ transcription during adipocyte differentiation [25,26].

All together the novel protein complex seems to act on c-fos promoter as ubiquitous transcriptional repressor. Mechanistically one can speculate that the complex alters DNA packaging, masks basal or activating promoter elements or interacts with transcription factors. The novel protein DNA complex identified might constitutively mimic a transcriptional repression process that usually is under tight regulation in cells [27].

The consequence of lowering c-fos expression is a differential gene expression pattern in our patient compared to controls. Further analyses shows that the reduced c-fos expression affects genes involved in multiple metabolic pathways, transcriptional control and a large fraction of genes involved in cell cycle control and differentiation processes most of them AP-1 dependent. Of special note are genes involved in adipocyte differentiation as Wnt5A, IL6, FGF-2, ODC-1, C/EBP, NR2F2, INHBB, SFRP1 or ITGA6. On the other hand gene functional annotation revealed that genes differentially expressed are also regulated by various adipose differentiation transcription factors as C/EBPs, PPARs or the SREBP family.

### Can the reduced c-fos promoter activity be related to CGL?

On cellular level the point mutation identified in our patient and the binding of the novel protein complex results in reduced basal as well as inducible c-fos expression but not complete loss of c-fos expression. This reduced c-fos expression influences expression on many other genes. This might be due to the fact that c-Fos doesn’t act as single transcription factor but is part of AP-1 complex. This protein complex consists of a combination of two proteins from various homologues proteins of Jun, ATF, MAF and Fos families, i.e. c-jun, JUNB, JUND, ATF2, ATF3/LRF1, B-ATF, JDP1, JDP2, c-Maf, MafB, MafA, MafG/F/K, Nrl, c-fos, Fra-1, Fra-2, FOSL or FosB. As consequence depending on dimer composition the transactivation activity of AP-1 complex varies from activation to repression of the target gene transactivation activity [9]. Furthermore, the occupancy of AP-1 sites by AP-1 transcription complex also influences transcription of overlapping or adjacent promoter elements. The direct competition of AP-1 to binding site CRE or ARE has been reported [27,28]. Knockout mice deficient for c-Fos revealed phenotypes with severe osteopetrosis and altered hematopoiesis. They show reduced fetal and placental weight, reduced weight gain and reduced fat mass, but to our knowledge there are no studies assessing further metabolic parameters [29-31]. However there is a reciprocal interaction between bone and energy metabolism [32]. Osteoblasts and adipocytes originate from a common mesenchymal progenitor and specific differentiation *via* BMPs and WNT pathways determine the cell fate to bone or adipose specific precursor cells [33]. This speculation is supported by mice overexpressing Fra-1 which develop lipodystrophy due to reduced adipocyte differentiation *via* C/EBPa inhibition and transcriptional repression [34]. Interestingly, patients with congenital lipodystrophy show increased bone age and density, enlarged epiphyses, sclerotic skeletons and alterations in dentition [35]. Furthermore the promoter activation of the immediately early gene c-fos is involved in various signaling cascades. One of those is IGF-1 signaling, that shares the signaling cascade and activation mechanisms of c-fos promoter as insulin [11]. As the role of IGF-1 and the GH/IGF-1 axis in various syndromes with growth restriction is well established [36] one can speculate that the growth alterations or skin and hair variations observed in the patient are most likely a consequence of interference with IGF-1 signaling.

## Conclusion

**Figure 6:**
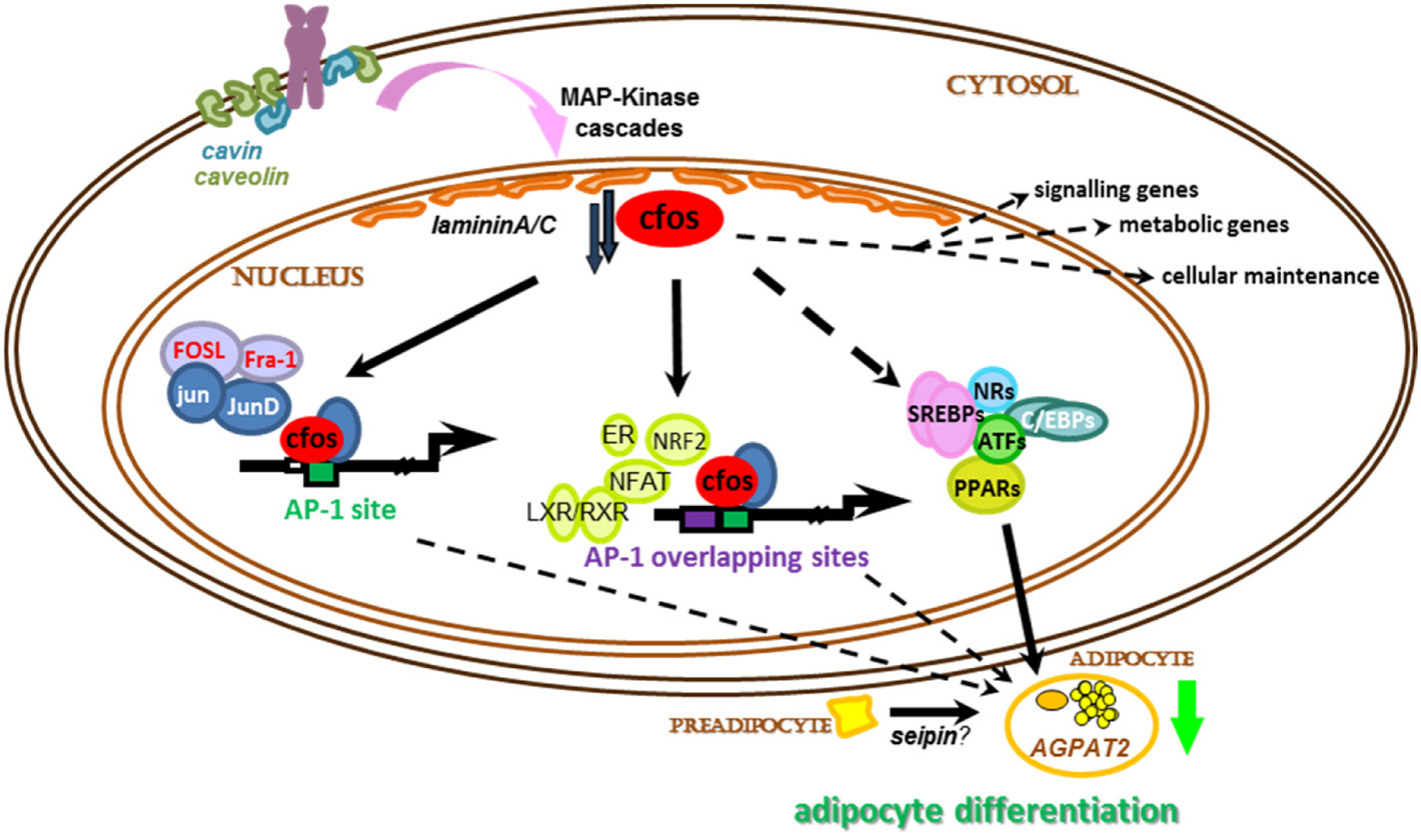
**Can deminished c-fos transactivation be one cause of adipose tissue malformation?** Postulated model how the identyfied c-fos promoter mutation affects signalling by cFos and AP-1 and interferes with adipocte differentiation. The BSCL genes (*italic;* seipin, AGPAT2, lamininA/C, caveolin, cavin) are included at the levels of functional interaction in c-fos signalling.

In conclusion we describe the identification of a *de novo* point mutation in the promoter of the immediately early transcription factor c-fos gene which might be associated with a CGL outcome. We show alteration of the transcription pattern in cells due to reduced c-fos expression in our patient. Furthermore we suggest a hypothetical model how reduced c-fos expression potentially interferes with target genes also necessary for differentiation or maturation of preadipocytes (Figure 6). Our findings provide evidence for the addition of c-Fos to the list of genes which might cause congenital lipodystrophy.

## Competing interests

The authors declare that they have no competing interests.

## Authors’ contributions

BK and JK were responsible for experimental design, interpretation, writing and editing of the manuscript. BK and JK further performed sequence analyses, gene expression analyses and *in silico* analyses. HA, SJ and MS. researched the *in vitro* data, SL, SH and UN performed experiments related to protein identifications. JH, WM and MMH provided control collectives and screened them for the mutation. ES was the referring physician of the patient; DM-W was the principal investigator and contributed to experimental design, interpretation of data, review and editing of the manuscript. All authors read and approved the final manuscript.

## Supplementary Material

Additional file 1: Table S1 Summary of differentially regulated transcripts. Equal amounts of total RNA of patient and 6 individual controls were used for expression analyses with Hu95A Arrays (Affymetrix). Expression data analyses utilizing standard algorithmus was performed with Genespring 12.0 to identify genes with statistic significant expression (p< 0.05) and a minimum 1.5- fold difference. For consensus site prediction web based tools as http://david.abcc.ncifcrf.gov/ were used. (FC: fold change to controls; p= significance of expression; min; max: minimum and maximum fold change observed in comparison to controls. Click here for file

Additional file 2: Table S2 GO annotation of regulated transcripts. Transcripts are listed in identical order as in Additional file 1: Table S1. Functional information is given as available. For consensus site prediction web based tools as http://david.abcc.ncifcrf.gov/ were used. Click here for file
