# Supplementary material for: A mutation in the c-Fos gene associated with congenital generalized lipodystrophy
Source: Orphanet J Rare Dis. 2013 Aug 7;8:119. doi: 10.1186/1750-1172-8-119 (PMC3750569; doi:10.1186/1750-1172-8-119)
Supplement: Additional file 1: Table S1 — Summary of differentially regulated transcripts. Equal amounts of total RNA of patient and 6 individual controls were used for expression analyses with Hu95A Arrays (Affymetrix). Expression data analyses utilizing standard algorithmus was performed with Genespring 12.0 to identify genes with statistic significant expression (p< 0.05) and a minimum 1.5- fold difference. For consensus site prediction web based tools as http://david.abcc.ncifcrf.gov/ were used. (FC: fold change to controls; p= significance of expression; min; max: minimum and maximum fold change observed in comparison to controls. [file 1750-1172-8-119-S1.docx]

**Supplement Table 1: Summary of differentially regulated transcripts**

Equal amounts of total RNA of patient and 6 individual controls were used for expression analyses with Hu95A Arrays (Affymetrix). Expression data analyses utilizing standard algorythms was performed with Genespring 12.0 toidentify genes with statistic significant expression (p< 0.05) and a minimum 1.5- fold difference. For consensus site prediction web based tools as http://david.abcc.ncifcrf.gov/ were used. (FC: fold change to controls; p= significance of expression; min; max: minimum and maxium fold change observed in comparison to controls.

**Upregulated genes in the patient containing an AP-1 consensus sequence**

| **Probe Set ID** | **RefSeq Transcript ID** | **Gene Symbol** | **Gene Title** | **FC (abs)** | **p** | **max** | **min** |
| --- | --- | --- | --- | --- | --- | --- | --- |
| 31862_at | NM_003392 | WNT5A | wingless-type MMTV integration site family, member 5A | 7.726 | 8.98E-03 | 9.982 | 5.980 |
| 38650_at | NM_000599 | IGFBP5 | insulin-like growth factor binding protein 5 | 6.568 | 2.25E-02 | 6.708 | 6.431 |
| 32186_at | NM_003486 | SLC7A5 | solute carrier family 7 (cationic amino acid transporter, y+ system), member 5 | 6.353 | 8.95E-03 | 7.975 | 5.061 |
| 1396_at | NM_000599 | IGFBP5 | insulin-like growth factor binding protein 5 | 5.423 | 1.58E-02 | 6.176 | 4.762 |
| 34778_at | NM_001135057; NM_130830 | LRRC15 | leucine rich repeat containing 15 | 5.042 | 1.14E-02 | 6.174 | 4.117 |
| 36543_at | NM_001178096; NM_001993 | F3 | coagulation factor III (thromboplastin, tissue factor) | 4.884 | 1.32E-02 | 6.271 | 3.803 |
| 38299_at | NM_000600 | IL6 | interleukin 6 (interferon, beta 2) | 4.874 | 2.71E-03 | 5.053 | 4.701 |
| 659_g_at | NM_003247 | THBS2 | thrombospondin 2 | 4.693 | 1.68E-02 | 5.264 | 4.183 |
| 581_at | NM_002291 | LAMB1 | laminin, beta 1 | 4.654 | 2.00E-02 | 6.275 | 3.453 |
| 38427_at | NM_001855 | COL15A1 | collagen, type XV, alpha 1 | 4.619 | 2.11E-03 | 7.367 | 3.700 |
| 1669_at | NM_003392 | WNT5A | wingless-type MMTV integration site family, member 5A | 4.610 | 3.08E-02 | 6.612 | 3.215 |
| 39827_at | NM_019058 | DDIT4 | DNA-damage-inducible transcript 4 | 4.338 | 9.77E-03 | 5.191 | 3.624 |
| 33127_at | NM_002318 | LOXL2 | lysyl oxidase-like 2 | 3.910 | 3.61E-02 | 5.688 | 2.688 |
| 2086_s_at | NM_006293 | TYRO3 | TYRO3 protein tyrosine kinase | 3.530 | 1.51E-02 | 4.362 | 2.857 |
| 33369_at | NM_001017369; NM_006745 | SC4MOL | sterol-C4-methyl oxidase-like | 3.489 | 1.78E-02 | 4.407 | 2.762 |
| 658_at | NM_003247 | THBS2 | thrombospondin 2 | 3.323 | 3.28E-02 | 5.212 | 2.184 |
| 34335_at | NM_004093 | EFNB2 | ephrin-B2 | 3.309 | 1.08E-02 | 5.311 | 1.050 |
| 36658_at | NM_014762 | DHCR24 | 24-dehydrocholesterol reductase | 3.216 | 3.10E-02 | 3.423 | 3.022 |
| 39127_f_at | NM_001193397; NM_021131; NM_178000; NM_178001; NM_178003 | PPP2R4 | protein phosphatase 2A activator, regulatory subunit 4 | 3.196 | 1.67E-02 | 3.960 | 2.580 |
| 133_at | NM_001114173; NM_001814; NM_148170 | CTSC | cathepsin C | 2.948 | 3.99E-05 | 3.358 | 2.768 |
| 35829_at | NM_001098517; NM_014333 | CADM1 | cell adhesion molecule 1 | 2.944 | 1.52E-02 | 3.471 | 2.496 |
| 37459_at | NM_001850; NM_020351 | COL8A1 | collagen, type VIII, alpha 1 | 2.898 | 2.46E-02 | 3.677 | 2.285 |
| 41778_at | NM_001145144; NM_001145145; NM_005628 | SLC1A5 | solute carrier family 1 (neutral amino acid transporter), member 5 | 2.849 | 1.14E-02 | 2.853 | 2.845 |
| 34849_at | NM_006513; NR_034072; NR_034073 | SARS | seryl-tRNA synthetase | 2.760 | 2.54E-02 | 3.389 | 2.248 |
| 38098_at | NM_145693 | LPIN1 | lipin 1 | 2.739 | 1.11E-02 | 2.990 | 2.509 |
| 40408_at | NM_001014437; NM_001194997; NM_001751; NM_139273; NR_036542 | CARS | cysteinyl-tRNA synthetase | 2.733 | 3.40E-03 | 2.955 | 2.527 |
| 38077_at | NM_004369; NM_057164; NM_057165; NM_057166; NM_057167 | COL6A3 | collagen, type VI, alpha 3 | 2.690 | 4.26E-03 | 2.846 | 2.543 |
| 32168_s_at | NM_004414; NM_203417; NM_203418 | RCAN1 | regulator of calcineurin 1 | 2.689 | 2.99E-02 | 3.362 | 2.150 |
| 37188_at | NM_001018073; NM_004563 | PCK2 | phosphoenolpyruvate carboxykinase 2 (mitochondrial) | 2.687 | 3.85E-02 | 3.190 | 2.264 |
| 36614_at | NM_005347 | HSPA5 | heat shock 70kDa protein 5 (glucose-regulated protein, 78kDa) | 2.687 | 2.36E-02 | 3.112 | 2.321 |
| 34853_at | NM_013231 | FLRT2 | fibronectin leucine rich transmembrane protein 2 | 2.680 | 6.28E-03 | 2.994 | 2.399 |
| 36937_s_at | NM_020992 | PDLIM1 | PDZ and LIM domain 1 | 2.665 | 1.41E-02 | 3.146 | 2.257 |
| 32488_at | NM_000090 | COL3A1 | collagen, type III, alpha 1 | 2.648 | 1.15E-02 | 3.787 | 1.998 |
| 36199_at | NM_004394 | DAP | death-associated protein | 2.646 | 4.37E-02 | 3.426 | 2.043 |
| 38474_at | NM_000071; NM_001178008; NM_001178009 | CBS | cystathionine-beta-synthase | 2.630 | 1.29E-02 | 3.037 | 2.278 |
| 36671_at | NM_001178075; NM_001178076; NM_001178077; NM_001673; NM_133436; NM_183356 | ASNS | asparagine synthetase (glutamine-hydrolyzing) | 2.620 | 3.29E-02 | 4.540 | 1.418 |
| 437_at | NM_002422 | MMP3 | matrix metallopeptidase 3 (stromelysin 1, progelatinase) | 2.619 | 4.47E-02 | 3.252 | 1.959 |
| 35246_at | NM_006293 | TYRO3 | TYRO3 protein tyrosine kinase | 2.612 | 7.59E-03 | 2.935 | 2.324 |
| 1828_s_at | NM_002006 | FGF2 | fibroblast growth factor 2 (basic) | 2.576 | 8.88E-03 | 3.502 | 2.039 |
| 32221_at | NM_014046 | MRPS18B | mitochondrial ribosomal protein S18B | 2.558 | 5.90E-03 | 2.774 | 2.360 |
| 38042_at | NM_002737 | PRKCA | synaptophysin-like 1 | 2.481 | 3.12E-03 | 2.969 | 1.064 |
| 222_at | NM_000127 | EXT1 | exostosin 1 | 2.469 | 6.45E-03 | 2.643 | 2.307 |
| 39382_at | NM_001130067; NM_015271 | TRIM2 | tripartite motif-containing 2 | 2.452 | 7.38E-03 | 3.417 | 1.225 |
| 33325_at | NM_001006932; NM_021135 | RPS6KA2 | ribosomal protein S6 kinase, 90kDa, polypeptide 2 | 2.441 | 1.78E-02 | 2.853 | 2.089 |
| 40613_at | NM_030939 | C6orf62 | chromosome 6 open reading frame 62 | 2.426 | 4.40E-02 | 3.180 | 1.851 |
| 37325_at | NM_001135821; NM_001135822; NM_002004 | FDPS | farnesyl diphosphate synthase | 2.426 | 2.52E-03 | 2.554 | 2.304 |
| 39782_at | NM_001190263; NM_001190265; NM_006333; NM_173177 | C1D | C1D nuclear receptor corepressor | 2.406 | 2.76E-02 | 2.904 | 1.994 |
| 719_g_at | NM_002775 | HTRA1 | HtrA serine peptidase 1 | 2.404 | 2.41E-03 | 2.549 | 2.266 |
| 39086_g_at | NM_003143 | SSBP1 | single-stranded DNA binding protein 1 | 2.401 | 4.88E-02 | 3.107 | 1.856 |
| 2036_s_at | NM_000610; NM_001001389; NM_001001390; NM_001001391; NM_001001392 | CD44 | CD44 molecule (Indian blood group) | 2.398 | 1.41E-02 | 5.160 | 1.180 |
| 37766_s_at | NM_002805 | PSMC5 | proteasome (prosome, macropain) 26S subunit, ATPase, 5 | 2.396 | 1.15E-02 | 2.491 | 2.305 |
| 32164_at | NM_000127 | EXT1 | exostosin 1 | 2.394 | 8.84E-03 | 2.616 | 2.191 |
| 40189_at | NM_001122821; NM_003011 | SET | SET nuclear oncogene | 2.384 | 4.55E-02 | 2.982 | 1.905 |
| 36009_at | NM_015696 | GPX7 | glutathione peroxidase 7 | 2.376 | 2.28E-03 | 3.190 | 1.342 |
| 38066_at | NM_000903; NM_001025433; NM_001025434 | NQO1 | NAD(P)H dehydrogenase, quinone 1 | 2.354 | 5.85E-03 | 2.543 | 2.178 |
| 297_g_at | NM_001069 | TUBB2A | tubulin, beta 2A | 2.351 | 4.60E-02 | 3.043 | 1.817 |
| 32171_at | NM_001969; NM_183004 | EIF5 | eukaryotic translation initiation factor 5 | 2.338 | 2.77E-02 | 2.828 | 1.933 |
| 36537_at | NM_001130955; NM_015318 | ARHGEF18 | Rho/Rac guanine nucleotide exchange factor (GEF) 18 | 2.335 | 3.99E-02 | 2.672 | 2.040 |
| 32546_at | NM_000143 | FH | fumarate hydratase | 2.326 | 4.77E-02 | 2.836 | 1.907 |
| 39759_at | NM_006775; NM_206853; NM_206854; NM_206855 | QKI | quaking homolog, KH domain RNA binding (mouse) | 2.320 | 9.89E-03 | 3.428 | 1.292 |
| 1521_at | NM_000269; NM_198175 | NME1 | non-metastatic cells 1, protein (NM23A) expressed in | 2.283 | 2.92E-04 | 2.329 | 2.238 |
| 38797_at | NM_001128431; NM_001135153; NM_001135154; NM_015359 | SLC39A14 | solute carrier family 39 (zinc transporter), member 14 | 2.268 | 3.24E-02 | 2.803 | 1.835 |
| 38815_at | NM_001190996; NM_006409 | ARPC1A | actin related protein 2/3 complex, subunit 1A, 41kDa | 2.261 | 1.99E-02 | 2.578 | 1.983 |
| 36472_at | NM_004688 | NMI | N-myc (and STAT) interactor | 2.255 | 2.89E-02 | 2.749 | 1.850 |
| 41133_at | NM_005754; NM_198395 | G3BP1 | GTPase activating protein (SH3 domain) binding protein 1 | 2.241 | 3.19E-03 | 4.859 | 1.378 |
| 33899_at | NM_000696 | ALDH9A1 | aldehyde dehydrogenase 9 family, member A1 | 2.238 | 2.23E-02 | 2.636 | 1.901 |
| 34198_at | NM_006264; NM_080683; NM_080684; NM_080685 | PTPN13 | protein tyrosine phosphatase, non-receptor type 13 (APO-1/CD95 (Fas)-associated phosphatase) | 2.233 | 4.91E-02 | 2.788 | 1.789 |
| 39363_at | NM_014453; NM_198426 | CHMP2A | chromatin modifying protein 2A | 2.223 | 8.36E-03 | 2.269 | 2.177 |
| 1388_g_at | NM_000376; NM_001017535 | VDR | vitamin D (1,25- dihydroxyvitamin D3) receptor | 2.222 | 3.36E-02 | 2.699 | 1.829 |
| 40099_at | NM_001162383; NM_001162384; NM_004723 | ARHGEF2 | Rho/Rac guanine nucleotide exchange factor (GEF) 2 | 2.213 | 2.94E-02 | 2.629 | 1.863 |
| 37671_at | NM_001105206; NM_001105207; NM_001105208; NM_001105209; NM_002290 | LAMA4 | laminin, alpha 4 | 2.212 | 4.54E-02 | 2.353 | 2.080 |
| 36203_at | NM_002539 | ODC1 | ornithine decarboxylase 1 | 2.193 | 2.99E-02 | 2.231 | 2.156 |
| 32363_at | NM_003956 | CH25H | cholesterol 25-hydroxylase | 2.192 | 3.45E-02 | 2.557 | 2.076 |
| 40541_at | NM_000050; NM_054012 | ASS1 | argininosuccinate synthase 1 | 2.189 | 2.05E-02 | 2.571 | 1.863 |
| 36308_at | NM_003412 | ZIC1 | Zic family member 1 (odd-paired homolog, Drosophila) | 2.172 | 1.95E-02 | 3.008 | 1.635 |
| 34376_at | NM_007066; NM_181804; NM_181805 | PKIG | protein kinase (cAMP-dependent, catalytic) inhibitor gamma | 2.165 | 1.16E-02 | 2.437 | 1.924 |
| 32669_at | NM_014011; NM_144949 | SOCS5 | suppressor of cytokine signaling 5 | 2.154 | 1.81E-02 | 3.728 | 1.116 |
| 32165_at | NM_001031684; NM_001195446 | SRSF7 | serine/arginine-rich splicing factor 7 | 2.148 | 2.86E-02 | 2.398 | 1.924 |
| 39532_at | NM_004292 | RIN1 | Ras and Rab interactor 1 | 2.147 | 2.77E-02 | 2.153 | 2.140 |
| 32542_at | NM_001159699; NM_001159700; NM_001159701; NM_001159702; NM_001159703; NM_001159704; NM_001167819; NM_001449; NR_027621 | FHL1 | four and a half LIM domains 1 | 2.142 | 1.09E-02 | 2.770 | 1.595 |
| 35324_at |  | --- | --- | 2.136 | 2.07E-02 | 4.059 | 3.066 |
| 1488_at | NM_001135648; NM_002844 | PTPRK | protein tyrosine phosphatase, receptor type, K | 2.135 | 3.73E-02 | 2.603 | 1.751 |
| 41439_at | NM_001130158; NM_001161819; NM_012223 | MYO1B | myosin IB | 2.129 | 1.68E-02 | 2.519 | 1.000 |
| 36976_at | NM_001797 | CDH11 | cadherin 11, type 2, OB-cadherin (osteoblast) | 2.127 | 4.97E-03 | 3.574 | 1.231 |
| 41256_at | NM_001130053; NM_001130055; NM_001130056; NM_001130057; NM_001195203; NM_001960; NM_032378 | EEF1D | eukaryotic translation elongation factor 1 delta (guanine nucleotide exchange protein) | 2.107 | 1.82E-03 | 2.195 | 2.022 |
| 33424_at | NM_002950 | RPN1 | ribophorin I | 2.076 | 1.35E-02 | 2.317 | 1.860 |
| 36572_r_at | NM_015161 | ARL6IP1 | ADP-ribosylation factor-like 6 interacting protein 1 | 2.072 | 3.97E-02 | 2.248 | 1.909 |
| 39710_at | NM_001142474; NM_001142475; NM_001142476; NM_001142477; NM_001142478; NM_001142479; NM_001142480; NM_001142481; NM_001142482; NM_001142483; NM_004772 | C5orf13 | chromosome 5 open reading frame 13 | 2.064 | 4.17E-03 | 2.708 | 1.127 |
| 34752_at | NM_133494 | NEK7 | NIMA (never in mitosis gene a)-related kinase 7 | 2.057 | 1.34E-02 | 2.522 | 1.097 |
| 36178_at | NM_001166356; NM_001166357; NM_001166358; NM_001166359; NM_005412; NR_029415; NR_029416; NR_029417 | SHMT2 | serine hydroxymethyltransferase 2 (mitochondrial) | 2.056 | 1.90E-02 | 2.368 | 1.784 |
| 32102_at | NM_014363 | SACS | spastic ataxia of Charlevoix-Saguenay (sacsin) | 2.044 | 3.41E-02 | 2.198 | 1.901 |
| 32695_at | NM_001163280; NM_014500 | HTATSF1 | HIV-1 Tat specific factor 1 | 2.031 | 1.36E-02 | 2.283 | 1.807 |
| 283_at | NM_003365 | UQCRC1 | ubiquinol-cytochrome c reductase core protein I | 2.027 | 8.51E-03 | 2.212 | 1.858 |
| 33294_at | NM_015004; NR_023353 | EXOSC7 | exosome component 7 | 2.027 | 2.69E-02 | 2.372 | 1.732 |
| 943_at | NM_001001890; NM_001122607; NM_001754 | RUNX1 | runt-related transcription factor 1 | 2.025 | 8.26E-03 | 2.152 | 1.906 |
| 39031_at | NM_001864 | COX7A1 | cytochrome c oxidase subunit VIIa polypeptide 1 (muscle) | 2.024 | 4.63E-02 | 2.059 | 1.989 |
| 1452_at | NM_006769 | LMO4 | LIM domain only 4 | 2.007 | 8.47E-03 | 2.139 | 1.884 |
| 38102_at | NM_152726 | EFHA1 | RAD21 homolog (S, pombe) | 2.007 | 2.49E-02 | 3.169 | 0.999 |
| 1778_g_at | NM_004292 | RIN1 | Ras and Rab interactor 1 | 1.996 | 5.94E-03 | 2.082 | 1.913 |
| 38483_at | NM_001143775; NM_015343 | CTDNEP1 | CTD nuclear envelope phosphatase 1 | 1.985 | 1.66E-02 | 2.252 | 1.750 |
| 32076_at | NM_005822 | RCAN2 | regulator of calcineurin 2 | 1.984 | 8.15E-04 | 2.029 | 1.939 |
| 33368_at | NM_015878; NM_148174 | AZIN1 | antizyme inhibitor 1 | 1.976 | 8.78E-03 | 1.981 | 1.092 |
| 40827_at | NM_002161; NM_013417 | IARS | isoleucyl-tRNA synthetase | 1.973 | 7.12E-03 | 2.361 | 1.583 |
| 39219_at | NM_001806 | CEBPG | CCAAT/enhancer binding protein (C/EBP), gamma | 1.972 | 6.09E-03 | 2.563 | 0.997 |
| 32849_at | NM_006306 | SMC1A | structural maintenance of chromosomes 1A | 1.970 | 4.89E-02 | 3.431 | 0.699 |
| 32781_f_at | NM_001723; NM_015548 | DST | dystonin | 1.966 | 3.49E-02 | 2.920 | 0.869 |
| 32551_at | NM_001039348; NM_001039349; NM_004105 | EFEMP1 | EGF-containing fibulin-like extracellular matrix protein 1 | 1.965 | 1.40E-02 | 2.194 | 1.183 |
| 41421_at | NM_001171166; NM_001171167; NM_001171168; NM_015099 | CAMTA2 | calmodulin binding transcription activator 2 | 1.959 | 6.19E-03 | 2.417 | 2.037 |
| 35396_at | NM_005328 | HAS2 | hyaluronan synthase 2 | 1.952 | 1.63E-03 | 2.678 | 2.479 |
| 33821_at | NM_021814 | ELOVL5 | ELOVL family member 5, elongation of long chain fatty acids (FEN1/Elo2, SUR4/Elo3-like, yeast) | 1.930 | 1.04E-02 | 2.681 | 0.833 |
| 831_at | NM_004398 | DDX10 | DEAD (Asp-Glu-Ala-Asp) box polypeptide 10 | 1.925 | 3.97E-03 | 1.957 | 1.893 |
| 40024_at | NM_003149 | STAC | SH3 and cysteine rich domain | 1.923 | 1.06E-02 | 1.821 | 1.107 |
| 718_at | NM_002775 | HTRA1 | HtrA serine peptidase 1 | 1.885 | 1.61E-02 | 2.038 | 1.744 |
| 31510_s_at | NM_005324 | H3F3B | H3 histone, family 3B (H3,3B) | 1.883 | 4.75E-02 | 2.306 | 1.538 |
| 37611_at | NM_002546 | TNFRSF11B | complement component 1, q subcomponent binding protein | 1.881 | 5.69E-03 | 2.296 | 1.054 |
| 40506_s_at | NM_001135653; NM_001135654; NM_003819 | PABPC4 | poly(A) binding protein, cytoplasmic 4 (inducible form) | 1.877 | 2.60E-02 | 2.766 | 1.080 |
| 34297_at | NM_005302 | GPR37 | G protein-coupled receptor 37 (endothelin receptor type B-like) | 1.873 | 1.46E-02 | 2.010 | 1.745 |
| 39878_at | NM_020403; NM_203487 | PCDH9 | protocadherin 9 | 1.860 | 6.90E-04 | 2.038 | 1.713 |
| 36960_at | NM_004427; NM_198040 | PHC2 | polyhomeotic homolog 2 (Drosophila) | 1.860 | 4.21E-02 | 2.102 | 1.646 |
| 32786_at | NM_002229 | JUNB | jun B proto-oncogene | 1.859 | 4.43E-02 | 2.193 | 1.576 |
| 37147_at | NM_002975 | CLEC11A | C-type lectin domain family 11, member A | 1.858 | 4.73E-02 | 2.173 | 1.589 |
| 36098_at | NM_001078166; NM_006924; NR_034041 | SRSF1 | serine/arginine-rich splicing factor 1 | 1.857 | 3.60E-02 | 2.162 | 1.595 |
| 38277_at | NM_001142353; NM_001142354; NM_021132 | PPP3CB | protein phosphatase 3, catalytic subunit, beta isozyme | 1.855 | 1.82E-02 | 2.925 | 0.937 |
| 33666_at | NM_001077442; NM_001077443; NM_004500; NM_031314 | HNRNPC | heterogeneous nuclear ribonucleoprotein C (C1/C2) | 1.853 | 1.64E-02 | 2.137 | 1.019 |
| 33203_s_at | NM_004472 | FOXD1 | forkhead box D1 | 1.850 | 7.69E-03 | 1.997 | 1.713 |
| 723_s_at | NM_001077442; NM_001077443; NM_004500; NM_031314 | HNRNPC | heterogeneous nuclear ribonucleoprotein C (C1/C2) | 1.849 | 4.12E-03 | 2.666 | 1.274 |
| 41385_at | NM_012307 | EPB41L3 | erythrocyte membrane protein band 4,1-like 3 | 1.847 | 8.51E-03 | 2.288 | 1.108 |
| 34336_at | NM_001130089; NM_005548 | KARS | lysyl-tRNA synthetase | 1.845 | 4.63E-02 | 2.230 | 1.527 |
| 2087_s_at | NM_001797 | CDH11 | cadherin 11, type 2, OB-cadherin (osteoblast) | 1.845 | 6.22E-03 | 2.519 | 1.132 |
| 39342_at | NM_004990 | MARS | methionyl-tRNA synthetase | 1.842 | 3.89E-02 | 2.195 | 1.546 |
| 37762_at | NM_001423 | EMP1 | epithelial membrane protein 1 | 1.837 | 8.91E-03 | 2.066 | 1.440 |
| 37336_at | NM_014607 | UBXN4 | UBX domain protein 4 | 1.833 | 1.47E-02 | 3.156 | 0.942 |
| 34396_at | NM_001164603; NM_015338 | ASXL1 | additional sex combs like 1 (Drosophila) | 1.825 | 1.64E-02 | 2.026 | 1.645 |
| 38354_at | NM_005194 | CEBPB | CCAAT/enhancer binding protein (C/EBP), beta | 1.817 | 3.92E-02 | 1.992 | 1.658 |
| 39945_at | NM_004460 | FAP | fibroblast activation protein, alpha | 1.800 | 2.07E-02 | 2.675 | 1.012 |
| 1314_at | NM_001191037; NM_002807; NR_034059 | PSMD1 | proteasome (prosome, macropain) 26S subunit, non-ATPase, 1 | 1.792 | 8.93E-03 | 1.910 | 1.681 |
| 38804_at | NM_001316 | CSE1L | CSE1 chromosome segregation 1-like (yeast) | 1.790 | 1.12E-02 | 1.825 | 1.756 |
| 31863_at | NM_015056 | RRP1B | ribosomal RNA processing 1 homolog B (S, cerevisiae) | 1.784 | 3.24E-02 | 1.875 | 1.698 |
| 40364_at | NM_001859 | SLC31A1 | solute carrier family 31 (copper transporters), member 1 | 1.783 | 1.61E-02 | 1.900 | 1.673 |
| 377_g_at | NM_006379 | SEMA3C | Rab geranylgeranyltransferase, beta subunit | 1.781 | 2.72E-02 | 1.635 | 1.129 |
| 34850_at | NM_006357; NM_182678 | UBE2E3 | ubiquitin-conjugating enzyme E2E 3 (UBC4/5 homolog, yeast) | 1.778 | 8.29E-03 | 2.237 | 1.226 |
| 35836_at | NM_006600 | NUDC | nuclear distribution gene C homolog (A, nidulans) | 1.764 | 3.03E-02 | 1.943 | 1.601 |
| 31801_at | NM_007114 | TMF1 | TATA element modulatory factor 1 | 1.764 | 5.40E-03 | 1.835 | 1.695 |
| 36131_at | NM_001288 | CLIC1 | chloride intracellular channel 1 | 1.763 | 2.74E-02 | 1.950 | 1.595 |
| 851_s_at | NM_005544 | IRS1 | insulin receptor substrate 1 | 1.763 | 2.11E-02 | 2.993 | 0.848 |
| 35729_at | NM_015194 | MYO1D | myosin ID | 1.759 | 3.11E-02 | 1.986 | 1.557 |
| 37406_at | NM_001143826; NM_001143827; NM_014268; NR_026570 | MAPRE2 | microtubule-associated protein, RP/EB family, member 2 | 1.757 | 4.77E-04 | 1.788 | 1.727 |
| 36926_at | NM_002748 | MAPK6 | mitogen-activated protein kinase 6 | 1.756 | 4.11E-02 | 1.815 | 1.699 |
| 2054_g_at | NM_001792 | CDH2 | cadherin 2, type 1, N-cadherin (neuronal) | 1.750 | 4.06E-03 | 2.148 | 1.175 |
| 37736_at | NM_005389 | PCMT1 | mesoderm specific transcript homolog (mouse) | 1.750 | 4.42E-02 | 2.637 | 0.786 |
| 37755_at | NM_014962; NM_181443 | BTBD3 | MyoD family inhibitor domain containing | 1.746 | 2.38E-02 | 1.772 | 1.018 |
| 38753_at | NM_007235 | XPOT | exportin, tRNA (nuclear export receptor for tRNAs) | 1.745 | 1.05E-02 | 1.944 | 1.309 |
| 41049_at | NM_005544 | IRS1 | insulin receptor substrate 1 | 1.741 | 1.80E-02 | 2.262 | 0.963 |
| 38818_at | NM_006415; NM_178324 | SPTLC1 | serine palmitoyltransferase, long chain base subunit 1 | 1.741 | 1.89E-02 | 2.588 | 0.895 |
| 36600_at | NM_006263; NM_176783 | PSME1 | proteasome (prosome, macropain) activator subunit 1 (PA28 alpha) | 1.739 | 4.16E-02 | 1.992 | 1.519 |
| 41776_at | NM_004045 | ATOX1 | ATX1 antioxidant protein 1 homolog (yeast) | 1.736 | 2.42E-02 | 1.823 | 1.654 |
| 688_at | NM_002802 | PSMC1 | proteasome (prosome, macropain) 26S subunit, ATPase, 1 | 1.734 | 1.56E-02 | 1.912 | 1.573 |
| 40556_at | NM_002901 | RCN1 | reticulocalbin 1, EF-hand calcium binding domain | 1.725 | 1.74E-03 | 1.928 | 1.198 |
| 36610_at | NM_015361 | R3HDM1 | R3H domain containing 1 | 1.725 | 1.45E-03 | 2.010 | 1.205 |
| 32112_s_at | NM_001624 | AIM1 | absent in melanoma 1 | 1.724 | 1.12E-02 | 3.134 | 1.136 |
| 34773_at | NM_004607 | TBCA | tubulin folding cofactor A | 1.719 | 3.84E-02 | 2.194 | 0.857 |
| 34823_at | NM_001935 | DPP4 | dipeptidyl-peptidase 4 | 1.717 | 1.69E-02 | 2.153 | 1.067 |
| 37966_at | NM_001003828; NM_013327 | PARVB | parvin, beta | 1.717 | 2.25E-02 | 1.765 | 1.670 |
| 39028_at | NM_002271 | IPO5 | importin 5 | 1.715 | 4.03E-02 | 1.569 | 1.040 |
| 41488_at | NM_001128301; NM_001128302; NM_020424 | LYRM1 | LYR motif containing 1 | 1.712 | 4.21E-02 | 3.145 | 1.863 |
| 33881_at | NM_004457; NM_203372 | ACSL3 | acyl-CoA synthetase long-chain family member 3 | 1.712 | 2.15E-02 | 1.839 | 1.593 |
| 35239_at | NM_000117 | EMD | emerin | 1.711 | 7.16E-03 | 1.822 | 1.607 |
| 41656_at | NM_004808 | NMT2 | N-myristoyltransferase 2 | 1.710 | 3.18E-02 | 2.116 | 1.521 |
| 37445_at | NM_014254 | TMEM5 | transmembrane protein 5 | 1.707 | 2.97E-02 | 2.361 | 0.972 |
| 34349_at | NM_007214 | SEC63 | SEC63 homolog (S, cerevisiae) | 1.706 | 1.73E-02 | 2.326 | 0.969 |
| 39756_g_at | NM_001079539; NM_005080 | XBP1 | X-box binding protein 1 | 1.705 | 4.84E-02 | 1.929 | 1.434 |
| 39389_at | NM_001769 | CD9 | CD9 molecule | 1.697 | 1.66E-02 | 2.087 | 1.929 |
| 37668_at | NM_001212 | C1QBP | neutral sphingomyelinase (N-SMase) activation associated factor | 1.697 | 3.09E-02 | 1.803 | 0.973 |
| 31897_at | NM_001042459; NM_014890; NM_182909 | FILIP1L | filamin A interacting protein 1-like | 1.693 | 4.86E-02 | 1.762 | 1.627 |
| 39744_at | NM_001193416; NM_001193417; NM_001356 | DDX3X | DEAD (Asp-Glu-Ala-Asp) box polypeptide 3, X-linked | 1.690 | 4.26E-02 | 2.649 | 1.042 |
| 34787_at | NM_001101662; NM_002525 | NRD1 | nardilysin (N-arginine dibasic convertase) | 1.689 | 3.98E-02 | 1.852 | 1.541 |
| 36963_at | NM_002631 | PGD | phosphogluconate dehydrogenase | 1.686 | 2.94E-03 | 1.755 | 1.620 |
| 571_at | NM_004537; NM_139207 | NAP1L1 | nucleosome assembly protein 1-like 1 | 1.686 | 3.27E-02 | 2.676 | 0.901 |
| 40832_s_at | NM_015602 | TOR1AIP1 | torsin A interacting protein 1 | 1.686 | 2.61E-02 | 1.592 | 1.090 |
| 39073_at | NM_000269; NM_198175 | NME1 | non-metastatic cells 1, protein (NM23A) expressed in | 1.683 | 4.03E-02 | 1.872 | 1.513 |
| 1250_at | NM_001081640; NM_006904 | PRKDC | protein kinase, DNA-activated, catalytic polypeptide | 1.679 | 1.62E-02 | 2.308 | 0.967 |
| 39800_s_at | NM_001018837; NM_006118 | HAX1 | HCLS1 associated protein X-1 | 1.678 | 8.60E-03 | 1.962 | 0.914 |
| 32408_s_at | NM_001013631; NM_001077442; NM_001077443; NM_001136561; NM_001146181; NM_004500; NM_031314 | HNRNPC; HNRNPCL1; LOC440563; LOC649330 | heterogeneous nuclear ribonucleoprotein C (C1/C2); heterogeneous nuclear ribonucleoprotein C-like 1; heterogeneous nuclear ribonucleoprotein C-like; heterogeneous nuclear ribonucleoprotein C-like | 1.676 | 3.12E-03 | 1.760 | 1.218 |
| 35298_at | NM_003753 | EIF3D | eukaryotic translation initiation factor 3, subunit D | 1.674 | 4.07E-02 | 1.857 | 1.508 |
| 36532_at | NM_001178088; NM_003898 | SYNJ2 | synaptojanin 2 | 1.667 | 3.02E-02 | 2.112 | 1.458 |
| 36474_at | NM_015323 | KIAA0776 | KIAA0776 | 1.664 | 3.43E-02 | 1.828 | 1.514 |
| 39397_at | NM_001145155; NM_001145156; NM_001145157; NM_021005 | NR2F2 | nuclear receptor subfamily 2, group F, member 2 | 1.662 | 2.80E-02 | 1.767 | 1.493 |
| 37334_at | NM_006805 | HNRNPA0 | heterogeneous nuclear ribonucleoprotein A0 | 1.659 | 5.81E-03 | 2.239 | 0.966 |
| 38527_at | NM_001145408; NM_001145409; NM_001145410; NM_007363 | NONO | non-POU domain containing, octamer-binding | 1.658 | 3.39E-02 | 1.874 | 1.466 |
| 41268_g_at | NM_014972 | TCF25 | transcription factor 25 (basic helix-loop-helix) | 1.653 | 9.65E-04 | 1.676 | 1.631 |
| 35785_at | NM_031412 | GABARAPL1 | GABA(A) receptor-associated protein like 1 | 1.653 | 2.80E-02 | 1.967 | 0.950 |
| 33247_at | NM_005805 | PSMD14 | proteasome (prosome, macropain) 26S subunit, non-ATPase, 14 | 1.652 | 4.72E-02 | 2.491 | 0.752 |
| 37142_at | NM_001145453; NM_005264; NM_145793 | GFRA1 | GDNF family receptor alpha 1 | 1.649 | 5.11E-03 | 1.977 | 1.167 |
| 39597_at | NM_014945 | ABLIM3 | actin binding LIM protein family, member 3 | 1.648 | 2.41E-02 | 1.770 | 1.534 |
| 38977_at | NM_003680 | YARS | tyrosyl-tRNA synthetase | 1.647 | 2.71E-02 | 2.013 | 0.788 |
| 32319_at | NM_003326 | TNFSF4 | tumor necrosis factor (ligand) superfamily, member 4 | 1.645 | 2.90E-02 | 1.956 | 1.559 |
| 41724_at | NM_001139441; NM_001139457; NM_005745; NR_024450 | BCAP31 | B-cell receptor-associated protein 31 | 1.644 | 2.50E-02 | 1.806 | 1.497 |
| 40875_s_at | NM_003089 | SNRNP70 | small nuclear ribonucleoprotein 70kDa (U1) | 1.643 | 1.42E-02 | 1.702 | 1.585 |
| 38012_at | NM_001999 | FBN2 | fibrillin 2 | 1.643 | 4.00E-02 | 2.047 | 1.353 |
| 41131_f_at | NM_001032393; NM_019597 | HNRNPH2 | heterogeneous nuclear ribonucleoprotein H2 (H') | 1.642 | 1.73E-02 | 1.735 | 1.553 |
| 37739_at | NM_003146 | SSRP1 | structure specific recognition protein 1 | 1.638 | 2.97E-02 | 1.725 | 1.555 |
| 40842_at | NM_004596 | SNRPA | small nuclear ribonucleoprotein polypeptide A | 1.636 | 1.75E-02 | 1.726 | 1.551 |
| 39347_at | NM_004069; NM_021575 | AP2S1 | adaptor-related protein complex 2, sigma 1 subunit | 1.635 | 4.23E-03 | 1.706 | 1.568 |
| 40910_at | NM_006135 | CAPZA1 | capping protein (actin filament) muscle Z-line, alpha 1 | 1.634 | 4.34E-02 | 1.864 | 1.433 |
| 36849_at | NM_004815 | ARHGAP29 | Rho GTPase activating protein 29 | 1.634 | 4.64E-03 | 1.917 | 1.492 |
| 36550_at | NM_018993 | RIN2 | Ras and Rab interactor 2 | 1.633 | 4.63E-02 | 1.650 | 1.617 |
| 36492_at | NM_002813 | PSMD9 | proteasome (prosome, macropain) 26S subunit, non-ATPase, 9 | 1.633 | 4.50E-02 | 1.898 | 1.404 |
| 956_at | NM_006088 | TUBB2C | tubulin, beta 2C | 1.631 | 5.45E-03 | 1.655 | 1.608 |
| 39029_at | NM_003910 | BUD31 | BUD31 homolog (S, cerevisiae) | 1.626 | 4.24E-02 | 1.735 | 1.524 |
| 39079_at | NM_004450 | ERH | enhancer of rudimentary homolog (Drosophila) | 1.626 | 7.69E-03 | 1.661 | 1.592 |
| 39056_at | NM_001079524; NM_001079525; NM_006452 | PAICS | phosphoribosylaminoimidazole carboxylase, phosphoribosylaminoimidazole succinocarboxamide synthetase | 1.625 | 1.30E-02 | 2.353 | 1.085 |
| 1629_s_at | NM_006264; NM_080683; NM_080684; NM_080685 | PTPN13 | protein tyrosine phosphatase, non-receptor type 13 (APO-1/CD95 (Fas)-associated phosphatase) | 1.623 | 1.38E-02 | 2.023 | 1.261 |
| 38364_at | NM_007005 | TLE4 | transducin-like enhancer of split 4 (E(sp1) homolog, Drosophila) | 1.622 | 1.81E-02 | 2.048 | 0.982 |
| 41419_at | NM_016315 | GULP1 | GULP, engulfment adaptor PTB domain containing 1 | 1.619 | 3.57E-03 | 1.798 | 1.327 |
| 37025_at | NM_001136472; NM_001136473; NM_004862; NR_024320 | LITAF | lipopolysaccharide-induced TNF factor | 1.617 | 4.89E-02 | 2.177 | 0.902 |
| 38007_at | NM_000268; NM_016418; NM_181825; NM_181828; NM_181829; NM_181830; NM_181831; NM_181832; NM_181833 | NF2 | neurofibromin 2 (merlin) | 1.617 | 6.64E-04 | 1.635 | 1.599 |
| 39420_at | NM_001195053; NM_001195054; NM_001195055; NM_001195056; NM_001195057; NM_004083 | DDIT3 | DNA-damage-inducible transcript 3 | 1.616 | 2.10E-02 | 1.664 | 1.569 |
| 41833_at | NM_006694 | JTB | jumping translocation breakpoint | 1.613 | 4.31E-02 | 2.772 | 0.830 |
| 36579_at | NM_004788 | UBE4A | ubiquitination factor E4A (UFD2 homolog, yeast) | 1.610 | 1.71E-02 | 2.249 | 0.855 |
| 39039_s_at | NM_016021 | UBE2J1 | ubiquitin-conjugating enzyme E2, J1 (UBC6 homolog, yeast) | 1.608 | 8.64E-03 | 1.970 | 1.034 |
| 41161_at | NM_001141969; NM_001141970; NM_001350; NR_024517 | DAXX | death-domain associated protein | 1.604 | 6.46E-04 | 1.615 | 1.594 |
| 32789_at | NM_001042540; NM_007362 | NCBP2 | nuclear cap binding protein subunit 2, 20kDa | 1.603 | 2.06E-02 | 1.672 | 1.537 |
| 38614_s_at | NM_181672; NM_181673 | OGT | O-linked N-acetylglucosamine (GlcNAc) transferase (UDP-N-acetylglucosamine:polypeptide-N-acetylglucosaminyl transferase) | 1.602 | 1.38E-02 | 1.870 | 1.387 |
| 34202_at | NM_015480 | PVRL3 | poliovirus receptor-related 3 | 1.600 | 2.55E-02 | 2.040 | 1.457 |
| 167_at | NM_001969; NM_183004 | EIF5 | eukaryotic translation initiation factor 5 | 1.599 | 3.74E-02 | 2.147 | 1.214 |
| 32305_at | NM_000089 | COL1A2 | collagen, type I, alpha 2 | 1.599 | 2.74E-02 | 2.274 | 1.310 |
| 40856_at | NM_002615 | SERPINF1 | serpin peptidase inhibitor, clade F (alpha-2 antiplasmin, pigment epithelium derived factor), member 1 | 1.597 | 1.38E-02 | 1.896 | 1.342 |
| 37842_at | NM_001166345; NM_001166346; NM_199072 | MDFIC | protein kinase C, alpha | 1.597 | 1.91E-02 | 1.752 | 1.124 |
| 36857_at | NM_002853; NR_026591 | RAD1 | RAD1 homolog (S, pombe) | 1.594 | 1.34E-02 | 2.699 | 1.171 |
| 40824_at | NM_015024 | XPO7 | exportin 7 | 1.594 | 4.75E-02 | 1.849 | 1.373 |
| 36617_at | NM_002165; NM_181353 | ID1 | inhibitor of DNA binding 1, dominant negative helix-loop-helix protein | 1.592 | 7.35E-03 | 1.674 | 1.514 |
| 34745_at | NM_014247 | RAPGEF2 | Rap guanine nucleotide exchange factor (GEF) 2 | 1.589 | 2.11E-02 | 1.674 | 0.982 |
| 37569_at | NM_013232 | PDCD6 | tumor necrosis factor receptor superfamily, member 11b | 1.589 | 4.29E-02 | 2.490 | 0.834 |
| 41407_at | NM_002904 | RDBP | RD RNA binding protein | 1.588 | 4.53E-02 | 1.720 | 1.465 |
| 40051_at | NM_012288 | TRAM2 | translocation associated membrane protein 2 | 1.588 | 4.50E-02 | 3.200 | 1.150 |
| 1385_at | NM_000358 | TGFBI | transforming growth factor, beta-induced, 68kDa | 1.587 | 4.86E-02 | 2.966 | 1.070 |
| 1310_at | NM_002794 | PSMB2 | proteasome (prosome, macropain) subunit, beta type, 2 | 1.584 | 4.10E-02 | 1.627 | 1.543 |
| 33877_s_at | NM_178812 | MTDH | metadherin | 1.583 | 4.73E-02 | 1.770 | 1.346 |
| 36633_at | NM_007202 | AKAP10 | A kinase (PRKA) anchor protein 10 | 1.583 | 3.50E-02 | 2.647 | 1.105 |
| 39665_at | NM_000824; NM_001166060; NM_001166061 | GLRB | glycine receptor, beta | 1.582 | 1.89E-02 | 1.610 | 1.088 |
| 38420_at | NM_000393 | COL5A2 | collagen, type V, alpha 2 | 1.575 | 7.15E-03 | 2.982 | 1.144 |
| 39139_at | NM_014300 | SEC11A | SEC11 homolog A (S, cerevisiae) | 1.575 | 2.22E-02 | 2.117 | 0.874 |
| 38770_at | NM_003474; NM_021641 | ADAM12 | ADAM metallopeptidase domain 12 | 1.574 | 4.96E-03 | 2.615 | 1.172 |
| 891_at | NM_003403 | YY1 | YY1 transcription factor | 1.573 | 4.67E-02 | 2.490 | 0.824 |
| 34880_at | NM_024321 | RBM42 | RNA binding motif protein 42 | 1.573 | 1.39E-02 | 1.597 | 1.550 |
| 39686_g_at | NM_001167621; NM_013236 | ATXN10 | ataxin 10 | 1.571 | 2.08E-02 | 1.723 | 0.814 |
| 34330_at | NM_004718 | COX7A2L | cytochrome c oxidase subunit VIIa polypeptide 2 like | 1.569 | 4.05E-02 | 1.816 | 0.867 |
| 37391_at | NM_001912; NM_145918 | CTSL1 | cathepsin L1 | 1.568 | 1.12E-02 | 1.573 | 1.562 |
| 31866_at | NM_019088 | PAF1 | Paf1, RNA polymerase II associated factor, homolog (S, cerevisiae) | 1.567 | 6.41E-03 | 1.597 | 1.538 |
| 32117_at | NM_012138 | AATF | apoptosis antagonizing transcription factor | 1.566 | 2.52E-02 | 1.734 | 1.415 |
| 34677_f_at | NM_004505; NM_032582; NR_003190; NR_003554 | LOC162632; LOC220594; USP32; USP6 | ubiquitin specific peptidase 6 (Tre-2 oncogene) pseudogene; ubiquitin specific peptidase 6 (Tre-2 oncogene) pseudogene; ubiquitin specific peptidase 32; ubiquitin specific peptidase 6 (Tre-2 oncogene) | 1.564 | 3.43E-02 | 2.590 | 0.923 |
| 38257_at | NM_002496 | NDUFS8 | solute carrier family 1 (neuronal/epithelial high affinity glutamate transporter, system Xag), member 1 | 1.564 | 1.54E-02 | 2.008 | 0.885 |
| 39795_at | NM_001025205; NM_004068 | AP2M1 | adaptor-related protein complex 2, mu 1 subunit | 1.561 | 4.97E-03 | 1.630 | 1.495 |
| 491_at | NM_002841 | PTPRG | protein tyrosine phosphatase, receptor type, G | 1.558 | 1.98E-02 | 1.890 | 1.348 |
| 41193_at | NM_001946; NM_022652 | DUSP6 | dual specificity phosphatase 6 | 1.558 | 4.59E-03 | 2.186 | 1.000 |
| 1444_at | NM_002813 | PSMD9 | proteasome (prosome, macropain) 26S subunit, non-ATPase, 9 | 1.553 | 2.49E-03 | 1.596 | 1.511 |
| 31950_at | NM_002568 | PABPC1 | poly(A) binding protein, cytoplasmic 1 | 1.552 | 3.01E-02 | 1.714 | 1.147 |
| 41126_at | NM_001193493; NM_003038 | SLC1A4 | solute carrier family 1 (glutamate/neutral amino acid transporter), member 4 | 1.551 | 4.23E-02 | 1.699 | 1.322 |
| 41333_at | NM_012287 | ACAP2 | ArfGAP with coiled-coil, ankyrin repeat and PH domains 2 | 1.550 | 2.14E-02 | 1.934 | 1.123 |
| 41690_at | NM_032199 | ARID5B | AT rich interactive domain 5B (MRF1-like) | 1.550 | 1.37E-02 | 1.867 | 1.035 |
| 39005_s_at | NM_015565 | LTN1 | listerin E3 ubiquitin protein ligase 1 | 1.545 | 2.49E-02 | 2.094 | 1.019 |
| 37347_at | NM_001826; NR_024163 | CKS1B | CDC28 protein kinase regulatory subunit 1B | 1.545 | 1.82E-02 | 1.680 | 1.421 |
| 33348_at | NM_003205; NM_207036; NM_207037; NM_207038; NM_207040 | TCF12 | transcription factor 12 | 1.544 | 1.70E-02 | 1.763 | 1.020 |
| 33362_at | NM_006449 | CDC42EP3 | CDC42 effector protein (Rho GTPase binding) 3 | 1.544 | 4.74E-02 | 1.823 | 0.833 |
| 1295_at | NM_001145138; NM_021975 | RELA | v-rel reticuloendotheliosis viral oncogene homolog A (avian) | 1.544 | 4.13E-02 | 1.547 | 1.541 |
| 37650_at | NM_001145125; NM_013446 | MKRN1 | makorin ring finger protein 1 | 1.542 | 4.40E-02 | 1.721 | 1.381 |
| 39792_at | NM_001102397; NM_001102398; NM_001102399; NM_005826 | HNRNPR | heterogeneous nuclear ribonucleoprotein R | 1.542 | 1.40E-02 | 1.628 | 1.459 |
| 36500_at | NM_001129765; NM_015922 | NSDHL | NAD(P) dependent steroid dehydrogenase-like | 1.541 | 4.75E-02 | 1.770 | 1.342 |
| 37685_at | NM_001008660; NM_007166 | PICALM | phosphatidylinositol binding clathrin assembly protein | 1.534 | 2.74E-02 | 1.674 | 1.405 |
| 1140_at | NM_002208 | ITGAE | integrin, alpha E (antigen CD103, human mucosal lymphocyte antigen 1; alpha polypeptide) | 1.532 | 4.59E-02 | 1.681 | 1.396 |
| 41342_at | NM_002882 | RANBP1 | RAN binding protein 1 | 1.531 | 1.63E-02 | 1.731 | 0.953 |
| 39839_at | NM_001145426; NM_003651 | CSDA | cold shock domain protein A | 1.529 | 1.99E-02 | 1.807 | 0.947 |
| 41547_at | NM_001007793; NM_004725 | BUB3 | budding uninhibited by benzimidazoles 3 homolog (yeast) | 1.529 | 4.93E-02 | 1.910 | 1.355 |
| 39755_at | NM_001079539; NM_005080 | XBP1 | X-box binding protein 1 | 1.525 | 1.44E-02 | 1.550 | 0.981 |
| 36014_at | NM_001032394; NM_001032395; NM_020455; NM_198569 | GPR126 | G protein-coupled receptor 126 | 1.524 | 5.28E-03 | 1.672 | 1.053 |
| 34783_s_at | NM_001007793; NM_004725 | BUB3 | budding uninhibited by benzimidazoles 3 homolog (yeast) | 1.524 | 2.86E-02 | 1.687 | 1.377 |
| 288_s_at | NM_002296; NM_194442 | LBR | lamin B receptor | 1.522 | 1.24E-02 | 1.800 | 0.893 |
| 40859_at | NM_001160103; NM_001160104; NM_024824; NM_207660; NM_207661; NM_207662 | ZC3H14 | zinc finger CCCH-type containing 14 | 1.521 | 3.57E-03 | 2.078 | 1.182 |
| 32134_at | NM_015641; NM_152829 | TES | testis derived transcript (3 LIM domains) | 1.520 | 1.16E-02 | 2.136 | 0.917 |
| 38985_at | NM_001128208; NM_015344 | LEPROTL1 | leptin receptor overlapping transcript-like 1 | 1.519 | 1.13E-02 | 1.547 | 1.490 |
| 35779_at | NM_007259 | VPS45 | vacuolar protein sorting 45 homolog (S, cerevisiae) | 1.517 | 2.32E-02 | 1.521 | 1.514 |
| 1695_at | NM_006156 | NEDD8 | neural precursor cell expressed, developmentally down-regulated 8 | 1.516 | 1.89E-02 | 1.642 | 1.400 |
| 37679_at | NM_001007245; NM_001197079; NM_001197080; NM_001550 | IFRD1 | sema domain, immunoglobulin domain (Ig), short basic domain, secreted, (semaphorin) 3C | 1.516 | 1.86E-02 | 1.996 | 1.113 |
| 36013_at | NM_006345 | SLC30A9 | solute carrier family 30 (zinc transporter), member 9 | 1.511 | 2.83E-02 | 1.663 | 1.373 |
| 36972_at | NM_006815 | TMED2 | transmembrane emp24 domain trafficking protein 2 | 1.511 | 2.77E-03 | 1.727 | 1.055 |
| 37046_at | NM_002790 | PSMA5 | proteasome (prosome, macropain) subunit, alpha type, 5 | 1.510 | 1.18E-02 | 1.547 | 1.084 |
| 40576_f_at | NM_031372; NR_003249 | HNRPDL | heterogeneous nuclear ribonucleoprotein D-like | 1.510 | 2.82E-02 | 1.577 | 1.446 |
| 33458_r_at | NM_001743 | CALM2 | calmodulin 2 (phosphorylase kinase, delta) | 1.507 | 1.40E-03 | 1.503 | 1.248 |
| 34433_at | NM_001197260; NM_001381 | DOK1 | docking protein 1, 62kDa (downstream of tyrosine kinase 1) | 1.503 | 1.70E-02 | 1.572 | 1.437 |
| 38121_at | NM_004184; NM_173701; NM_213645; NM_213646 | WARS | tryptophanyl-tRNA synthetase | 1.502 | 1.83E-03 | 1.520 | 1.483 |

**Upregulated genes in the patient without AP-1 consensus sequence**

| **Probe Set ID** | **RefSeq Transcript ID** | **Gene Symbol** | **Gene Title** | **FC (abs)** | **p** | **max** | **min** |
| --- | --- | --- | --- | --- | --- | --- | --- |
| 1451_s_at | NM_001135934; NM_001135935; NM_001135936; NM_006475 | POSTN | periostin, osteoblast specific factor | 8.425 | 4.58E-02 | 16.156 | 1.793 |
| 40698_at | NM_005127 | CLEC2B | C-type lectin domain family 2, member B | 7.935 | 5.51E-03 | 9.701 | 6.490 |
| 40074_at | NM_006636; NR_027405 | MTHFD2 | methylenetetrahydrofolate dehydrogenase (NADP+ dependent) 2, methenyltetrahydrofolate cyclohydrolase | 6.881 | 2.69E-02 | 10.779 | 4.393 |
| 36861_at | NM_015419 | MXRA5 | matrix-remodelling associated 5 | 4.877 | 2.13E-03 | 5.409 | 4.397 |
| 39337_at | NM_002106 | H2AFZ | H2A histone family, member Z | 3.869 | 3.63E-02 | 5.371 | 2.787 |
| 36582_g_at | NM_002047 | GARS | glycyl-tRNA synthetase | 3.584 | 1.83E-02 | 4.563 | 2.815 |
| 40916_at | NM_001080425; NM_001127688 | BEX4 | brain expressed, X-linked 4 | 3.431 | 2.74E-04 | 3.509 | 3.355 |
| 40161_at | NM_000095 | COMP | cartilage oligomeric matrix protein | 3.370 | 1.89E-02 | 3.480 | 3.264 |
| 39354_at | NM_004905 | PRDX6 | peroxiredoxin 6 | 3.216 | 8.50E-04 | 3.823 | 2.838 |
| 34390_at | NM_001017973; NM_001017974; NM_001142598; NM_001142599; NM_004199 | P4HA2 | prolyl 4-hydroxylase, alpha polypeptide II | 2.959 | 1.84E-02 | 3.512 | 2.493 |
| 36581_at | NM_002047 | GARS | glycyl-tRNA synthetase | 2.916 | 9.46E-03 | 3.378 | 2.517 |
| 1450_g_at | NM_001102667; NM_001102668; NM_002789 | PSMA4 | proteasome (prosome, macropain) subunit, alpha type, 4 | 2.827 | 1.89E-02 | 3.469 | 2.304 |
| 35814_at | NM_006360 | EIF3M | eukaryotic translation initiation factor 3, subunit M | 2.790 | 4.15E-02 | 3.761 | 2.069 |
| 40813_at | NM_006933 | SLC5A3 | solute carrier family 5 (sodium/myo-inositol cotransporter), member 3 | 2.728 | 1.10E-02 | 3.108 | 2.395 |
| 35956_s_at | NM_002780; NM_002783; NM_213633 | PSG4; PSG7 | pregnancy specific beta-1-glycoprotein 4; pregnancy specific beta-1-glycoprotein 7 (gene/pseudogene) | 2.704 | 4.33E-02 | 3.590 | 1.501 |
| 37050_r_at | NM_006809 | TOMM34 | translocase of outer mitochondrial membrane 34 | 2.681 | 1.74E-04 | 2.728 | 2.634 |
| 32825_at | NM_001536; NM_198318; NR_033397 | PRMT1 | protein arginine methyltransferase 1 | 2.662 | 4.94E-03 | 2.935 | 2.414 |
| 40103_at | NM_001111077; NM_003379 | EZR | ezrin | 2.611 | 2.30E-02 | 2.688 | 2.537 |
| 35367_at | NM_001177388; NM_002306; NR_003225 | LGALS3 | lectin, galactoside-binding, soluble, 3 | 2.576 | 2.62E-02 | 2.210 | 2.117 |
| 38479_at | NM_006401 | ANP32B | acidic (leucine-rich) nuclear phosphoprotein 32 family, member B | 2.568 | 9.84E-03 | 2.691 | 2.450 |
| 41237_at | NM_002116 | HLA-A | major histocompatibility complex, class I, A | 2.551 | 1.33E-02 | 2.569 | 2.533 |
| 39733_at | NM_001010989; NM_001010990; NM_014685 | HERPUD1 | homocysteine-inducible, endoplasmic reticulum stress-inducible, ubiquitin-like domain member 1 | 2.546 | 3.45E-02 | 3.276 | 1.979 |
| 38679_g_at | NM_003094 | SNRPE | small nuclear ribonucleoprotein polypeptide E | 2.515 | 4.36E-02 | 3.332 | 1.898 |
| 38038_at | NM_002345 | LUM | lumican | 2.501 | 4.65E-02 | 3.300 | 1.895 |
| 37749_at | NM_002402; NM_177524; NM_177525 | MEST | BTB (POZ) domain containing 3 | 2.476 | 3.11E-02 | 2.329 | 1.052 |
| 41523_at | NM_006834 | RAB32 | RAB32, member RAS oncogene family | 2.445 | 4.48E-02 | 3.140 | 1.903 |
| 36171_at | NM_006713 | SUB1 | SUB1 homolog (S, cerevisiae) | 2.392 | 4.23E-02 | 3.084 | 1.856 |
| 40162_s_at | NM_000095 | COMP | cartilage oligomeric matrix protein | 2.373 | 2.76E-02 | 2.894 | 1.947 |
| 40774_at | NM_001008800; NM_005998; NR_036564; NR_036565 | CCT3 | chaperonin containing TCP1, subunit 3 (gamma) | 2.369 | 2.40E-02 | 2.861 | 1.961 |
| 195_s_at | NM_001225; NM_033306 | CASP4 | caspase 4, apoptosis-related cysteine peptidase | 2.365 | 7.80E-03 | 2.601 | 2.150 |
| 34368_at | NM_001527; NR_033441 | HDAC2 | histone deacetylase 2 | 2.323 | 4.75E-02 | 3.000 | 1.799 |
| 39169_at | NM_001012456; NM_014302 | SEC61G | Sec61 gamma subunit | 2.297 | 1.36E-02 | 2.635 | 2.003 |
| 36851_g_at | NM_006765; NM_178234 | TUSC3 | tumor suppressor candidate 3 | 2.273 | 3.92E-02 | 2.520 | 2.051 |
| 37692_at | NM_001079862; NM_001079863; NM_001178017; NM_001178041; NM_001178042; NM_001178043; NM_020548 | DBI | diazepam binding inhibitor (GABA receptor modulator, acyl-CoA binding protein) | 2.270 | 1.18E-02 | 2.356 | 2.187 |
| 36088_at | NM_003720; NM_203433 | PSMG1 | proteasome (prosome, macropain) assembly chaperone 1 | 2.248 | 3.46E-02 | 2.654 | 1.904 |
| 37459_at | NM_001850; NM_020351 | COL8A1 | programmed cell death 6 | 2.243 | 1.28E-02 | 4.333 | 1.064 |
| 32510_at | NM_003689 | AKR7A2 | aldo-keto reductase family 7, member A2 (aflatoxin aldehyde reductase) | 2.240 | 3.58E-02 | 2.772 | 1.811 |
| 36159_s_at | NM_000311; NM_001080121; NM_001080122; NM_001080123; NM_183079 | PRNP | prion protein | 2.239 | 4.25E-03 | 2.887 | 1.137 |
| 41403_at | NM_003095 | SNRPF | small nuclear ribonucleoprotein polypeptide F | 2.224 | 2.56E-02 | 2.671 | 1.853 |
| 36985_at | NM_004508 | IDI1 | isopentenyl-diphosphate delta isomerase 1 | 2.177 | 1.36E-02 | 2.302 | 2.059 |
| 37720_at | NM_002156; NM_199440 | HSPD1 | heat shock 60kDa protein 1 (chaperonin) | 2.163 | 3.57E-02 | 2.669 | 1.752 |
| 41250_at | NM_006303 | AIMP2 | aminoacyl tRNA synthetase complex-interacting multifunctional protein 2 | 2.136 | 1.28E-02 | 2.368 | 1.927 |
| 33113_at | NM_001168388; NM_001168389; NM_006079 | CITED2 | Cbp/p300-interacting transactivator, with Glu/Asp-rich carboxy-terminal domain, 2 | 2.136 | 1.22E-02 | 3.960 | 1.285 |
| 1073_at | NM_006756; NM_201437 | TCEA1 | transcription elongation factor A (SII), 1 | 2.135 | 1.50E-02 | 2.625 | 1.740 |
| 36172_s_at | NM_001077523; NM_003938 | AP3D1 | adaptor-related protein complex 3, delta 1 subunit | 2.131 | 4.05E-02 | 2.432 | 1.867 |
| 41824_at | NM_016001 | UTP18 | UTP18, small subunit (SSU) processome component, homolog (yeast) | 2.116 | 4.58E-02 | 2.232 | 2.007 |
| 38029_at | NM_001012661; NM_001012662; NM_001012663; NM_001012664; NM_001013251; NM_002394 | SLC3A2 | solute carrier family 3 (activators of dibasic and neutral amino acid transport), member 2 | 2.101 | 1.10E-02 | 2.183 | 2.022 |
| 36654_s_at | NM_002137; NM_031243 | HNRNPA2B1 | heterogeneous nuclear ribonucleoprotein A2/B1 | 2.079 | 2.24E-02 | 4.373 | 1.181 |
| 39173_at | NM_001436 | FBL | fibrillarin | 2.078 | 7.59E-03 | 2.162 | 1.998 |
| 37640_at | NM_000194 | HPRT1 | hypoxanthine phosphoribosyltransferase 1 | 2.078 | 2.84E-02 | 2.320 | 1.860 |
| 39722_at | NM_001010858 | RNF187 | ring finger protein 187 | 2.075 | 1.82E-03 | 2.113 | 2.038 |
| 1945_at | NM_031966 | CCNB1 | cyclin B1 | 2.074 | 4.81E-02 | 2.492 | 1.727 |
| 31492_at | NM_013234 | EIF3K | eukaryotic translation initiation factor 3, subunit K | 2.072 | 2.61E-02 | 2.381 | 1.803 |
| 36975_at | NM_016127 | TMEM66 | transmembrane protein 66 | 2.069 | 2.77E-02 | 3.898 | 0.773 |
| 35792_at | NM_001003794; NM_007283 | MGLL | monoglyceride lipase | 2.067 | 1.59E-02 | 2.167 | 1.973 |
| 39092_at | NM_012412; NM_138635; NM_201436; NM_201516; NM_201517 | H2AFV | H2A histone family, member V | 2.041 | 2.77E-02 | 3.811 | 0.879 |
| 41765_at | NM_007209 | RPL35 | ribosomal protein L35 | 2.033 | 4.64E-02 | 2.243 | 1.842 |
| 38368_at | NM_001025248; NM_001025249; NM_001948 | DUT | deoxyuridine triphosphatase | 2.024 | 1.08E-02 | 2.240 | 1.829 |
| 39748_at | NM_003045 | SLC7A1 | solute carrier family 7 (cationic amino acid transporter, y+ system), member 1 | 2.018 | 1.56E-02 | 2.407 | 0.754 |
| 39357_at | NM_006331 | EMG1 | EMG1 nucleolar protein homolog (S, cerevisiae) | 2.017 | 1.50E-02 | 2.060 | 1.975 |
| 38916_at | NM_001177465; NM_001177466; NM_005491 | MAMLD1 | mastermind-like domain containing 1 | 2.013 | 2.26E-02 | 2.330 | 1.739 |
| 32853_at | NM_014820 | TOMM70A | translocase of outer mitochondrial membrane 70 homolog A (S, cerevisiae) | 2.004 | 2.81E-02 | 2.358 | 1.704 |
| 40839_at | NM_007106 | UBL3 | ubiquitin-like 3 | 1.998 | 1.72E-02 | 2.077 | 0.914 |
| 38744_at | NM_006304 | SHFM1 | split hand/foot malformation (ectrodactyly) type 1 | 1.975 | 1.24E-02 | 2.089 | 1.868 |
| 36517_at | NM_001025203; NM_001025204; NM_006758 | U2AF1 | U2 small nuclear RNA auxiliary factor 1 | 1.938 | 3.21E-02 | 2.054 | 1.828 |
| 1447_at | NM_002793 | PSMB1 | proteasome (prosome, macropain) subunit, beta type, 1 | 1.935 | 2.82E-02 | 2.166 | 1.730 |
| 1287_at | NM_001618 | PARP1 | poly (ADP-ribose) polymerase 1 | 1.930 | 1.20E-03 | 1.993 | 1.869 |
| 35983_at | NM_024100 | WDR18 | WD repeat domain 18 | 1.905 | 3.05E-03 | 1.935 | 1.874 |
| 41242_at | NM_003115 | UAP1 | UDP-N-acteylglucosamine pyrophosphorylase 1 | 1.892 | 9.96E-03 | 2.186 | 1.052 |
| 35916_s_at | NM_194247 | HNRNPA3 | heterogeneous nuclear ribonucleoprotein A3 | 1.875 | 3.20E-02 | 2.085 | 1.685 |
| 36685_at | NM_001033059; NM_001634 | AMD1 | adenosylmethionine decarboxylase 1 | 1.869 | 2.91E-03 | 1.884 | 1.854 |
| 37717_at | NM_005968; NM_031203 | HNRNPM | heterogeneous nuclear ribonucleoprotein M | 1.867 | 1.88E-02 | 2.098 | 1.661 |
| 38404_at | NM_004613; NM_198951 | TGM2 | transglutaminase 2 (C polypeptide, protein-glutamine-gamma-glutamyltransferase) | 1.866 | 2.85E-02 | 2.065 | 1.686 |
| 34736_at | NM_031966 | CCNB1 | cyclin B1 | 1.864 | 7.52E-03 | 1.963 | 1.769 |
| 39070_at | NM_003088 | FSCN1 | fascin homolog 1, actin-bundling protein (Strongylocentrotus purpuratus) | 1.861 | 1.05E-02 | 1.978 | 1.751 |
| 40607_at | NM_001197293; NM_001386 | DPYSL2 | dihydropyrimidinase-like 2 | 1.845 | 4.39E-02 | 2.615 | 1.399 |
| 32563_at | NM_001679 | ATP1B3 | ATPase, Na+/K+ transporting, beta 3 polypeptide | 1.844 | 2.45E-02 | 2.033 | 1.673 |
| 40546_s_at | NM_001185012; NM_002488; NR_033697 | NDUFA2 | NADH dehydrogenase (ubiquinone) 1 alpha subcomplex, 2, 8kDa | 1.843 | 2.98E-02 | 2.146 | 1.582 |
| 38708_at | NM_006325 | RAN | RAN, member RAS oncogene family | 1.842 | 2.90E-02 | 3.374 | 0.912 |
| 38275_at | NM_033416 | IMP4 | IMP4, U3 small nucleolar ribonucleoprotein, homolog (yeast) | 1.838 | 1.46E-02 | 1.995 | 1.693 |
| 706_at | NM_000176; NM_001018074; NM_001018075; NM_001018076; NM_001018077; NM_001020825; NM_001024094 | NR3C1 | nuclear receptor subfamily 3, group C, member 1 (glucocorticoid receptor) | 1.834 | 6.01E-03 | 1.600 | 1.291 |
| 306_s_at | NM_004965 | HMGN1 | high-mobility group nucleosome binding domain 1 | 1.832 | 4.58E-03 | 2.356 | 1.036 |
| 40417_at | NM_012073 | CCT5 | chaperonin containing TCP1, subunit 5 (epsilon) | 1.817 | 2.55E-02 | 2.316 | 0.770 |
| 41146_at | NM_001618 | PARP1 | poly (ADP-ribose) polymerase 1 | 1.816 | 1.49E-02 | 1.826 | 1.807 |
| 39088_at | NM_032635 | TMEM147 | transmembrane protein 147 | 1.814 | 4.11E-02 | 2.043 | 1.610 |
| 37673_at | NM_001144772; NM_003580 | NSMAF | BMP and activin membrane-bound inhibitor homolog (Xenopus laevis) | 1.814 | 7.59E-03 | 2.368 | 1.051 |
| 35300_at | NM_004446 | EPRS | glutamyl-prolyl-tRNA synthetase | 1.811 | 2.20E-02 | 2.287 | 0.929 |
| 33819_at | NM_001174097; NM_002300 | LDHB | lactate dehydrogenase B | 1.807 | 3.40E-02 | 2.108 | 1.549 |
| 36616_at | NM_001136264; NM_001136266; NM_001136267; NM_001136268; NM_001136269; NM_014764 | DAZAP2 | DAZ associated protein 2 | 1.804 | 4.21E-02 | 2.146 | 1.516 |
| 33865_at | NM_001161482; NM_006624; NM_212479 | ZMYND11 | zinc finger, MYND domain containing 11 | 1.802 | 5.53E-03 | 2.616 | 1.185 |
| 31824_at | NM_002395 | ME1 | malic enzyme 1, NADP(+)-dependent, cytosolic | 1.800 | 3.01E-02 | 2.532 | 0.866 |
| 41749_at | NM_004649; NM_198155 | C21orf33 | chromosome 21 open reading frame 33 | 1.799 | 3.24E-02 | 2.033 | 1.593 |
| 35153_at | NM_002485 | NBN | nibrin | 1.791 | 1.81E-02 | 2.794 | 0.883 |
| 1848_at | NM_001010935; NM_002884 | RAP1A | RAP1A, member of RAS oncogene family | 1.781 | 8.02E-03 | 2.241 | 1.190 |
| 37389_at | NM_001142705; NM_014267 | C11orf58 | chromosome 11 open reading frame 58 | 1.780 | 4.95E-03 | 2.226 | 1.291 |
| 32051_at | NM_001007027; NM_024079 | ALG8 | asparagine-linked glycosylation 8, alpha-1,3-glucosyltransferase homolog (S, cerevisiae) | 1.775 | 4.69E-02 | 1.848 | 1.704 |
| 32434_at | NM_002356 | MARCKS | myristoylated alanine-rich protein kinase C substrate | 1.768 | 1.70E-02 | 2.193 | 1.401 |
| 41510_s_at | NM_004134 | HSPA9 | heat shock 70kDa protein 9 (mortalin) | 1.767 | 4.40E-02 | 1.892 | 1.649 |
| 35714_at | NM_003681 | PDXK | pyridoxal (pyridoxine, vitamin B6) kinase | 1.764 | 4.07E-02 | 1.950 | 1.596 |
| 37462_i_at | NM_007165 | SF3A2 | splicing factor 3a, subunit 2, 66kDa | 1.759 | 6.88E-03 | 1.877 | 1.649 |
| 41185_f_at | NM_001005849; NM_006937 | SUMO2 | SMT3 suppressor of mif two 3 homolog 2 (S, cerevisiae) | 1.748 | 4.78E-02 | 2.048 | 1.492 |
| 38605_at | NM_004545 | NDUFB1 | NADH dehydrogenase (ubiquinone) 1 beta subcomplex, 1, 7kDa | 1.747 | 4.58E-02 | 2.020 | 1.511 |
| 229_at | NM_005760 | CEBPZ | CCAAT/enhancer binding protein (C/EBP), zeta | 1.746 | 2.65E-02 | 1.988 | 1.533 |
| 37726_at | NM_007208 | MRPL3 | protein-L-isoaspartate (D-aspartate) O-methyltransferase | 1.745 | 1.81E-02 | 1.948 | 0.980 |
| 38720_at | NM_001009570; NM_001166284; NM_001166285; NM_006429; NR_029402; NR_029403 | CCT7 | chaperonin containing TCP1, subunit 7 (eta) | 1.742 | 3.62E-02 | 2.021 | 1.501 |
| 38676_at | NM_006948 | HSPA13 | heat shock protein 70kDa family, member 13 | 1.735 | 1.83E-02 | 2.038 | 0.907 |
| 38589_i_at | NM_001099285; NM_002823 | PTMA | prothymosin, alpha | 1.734 | 1.46E-02 | 2.263 | 0.910 |
| 39801_at | NM_001084 | PLOD3 | procollagen-lysine, 2-oxoglutarate 5-dioxygenase 3 | 1.733 | 1.56E-02 | 1.745 | 1.721 |
| 38687_at | NM_015391; NR_024400; NR_024401 | ANAPC13 | anaphase promoting complex subunit 13 | 1.731 | 1.54E-02 | 2.328 | 0.926 |
| 38974_at | NM_001123377; NM_007262 | PARK7 | Parkinson disease (autosomal recessive, early onset) 7 | 1.731 | 1.29E-02 | 1.891 | 1.584 |
| 34199_at | NM_021982 | SEC24A | SEC24 family, member A (S, cerevisiae) | 1.730 | 3.51E-02 | 1.996 | 1.499 |
| 1789_at | NM_006837 | COPS5 | COP9 constitutive photomorphogenic homolog subunit 5 (Arabidopsis) | 1.719 | 4.32E-02 | 2.230 | 0.763 |
| 34862_at | NM_016002 | SCCPDH | saccharopine dehydrogenase (putative) | 1.719 | 2.83E-02 | 1.925 | 1.534 |
| 40211_at | NM_002136; NM_031157 | HNRNPA1 | heterogeneous nuclear ribonucleoprotein A1 | 1.716 | 8.47E-03 | 2.683 | 1.105 |
| 35818_at | NM_018947 | CYCS | cytochrome c, somatic | 1.714 | 4.83E-02 | 2.589 | 0.846 |
| 38837_at | NM_021156 | TMX4 | thioredoxin-related transmembrane protein 4 | 1.707 | 3.27E-02 | 1.713 | 1.004 |
| 1313_at | NM_002799 | PSMB7 | proteasome (prosome, macropain) subunit, beta type, 7 | 1.703 | 4.15E-02 | 1.994 | 1.455 |
| 1052_s_at | NM_005195 | CEBPD | CCAAT/enhancer binding protein (C/EBP), delta | 1.687 | 4.55E-02 | 1.901 | 1.498 |
| 38114_at | NM_006265 | RAD21 | syndecan 1 | 1.686 | 1.35E-02 | 2.353 | 1.151 |
| 36624_at | NM_000884 | IMPDH2 | IMP (inosine 5'-monophosphate) dehydrogenase 2 | 1.678 | 4.38E-02 | 1.888 | 1.492 |
| 339_at | NM_001233; NM_198212 | CAV2 | caveolin 2 | 1.676 | 4.02E-02 | 3.089 | 0.986 |
| 36090_at | NM_012453 | TBL2 | transducin (beta)-like 2 | 1.673 | 4.46E-02 | 1.841 | 1.520 |
| 39003_at | NM_004339 | PTTG1IP | pituitary tumor-transforming 1 interacting protein | 1.672 | 3.20E-02 | 1.910 | 1.464 |
| 37311_at | NM_006755 | TALDO1 | transaldolase 1 | 1.672 | 4.90E-03 | 1.719 | 1.627 |
| 34825_at | NM_016614 | TDP2 | tyrosyl-DNA phosphodiesterase 2 | 1.671 | 2.47E-02 | 1.831 | 1.524 |
| 39731_at | NM_001164803; NM_002139; NR_028476; NR_028477 | RBMX | RNA binding motif protein, X-linked | 1.670 | 3.26E-02 | 2.382 | 0.952 |
| 36185_at | NM_001605 | AARS | alanyl-tRNA synthetase | 1.655 | 2.66E-02 | 1.778 | 1.540 |
| 40108_at | NM_014670 | BZW1 | basic leucine zipper and W2 domains 1 | 1.651 | 4.47E-02 | 2.680 | 0.818 |
| 32119_at |  | --- | --- | 1.646 | 1.96E-02 | 1.739 | 1.559 |
| 39328_at | NM_000859; NM_001130996 | HMGCR | 3-hydroxy-3-methylglutaryl-CoA reductase | 1.642 | 3.01E-02 | 1.827 | 1.476 |
| 40125_at | NM_001024649; NM_001746 | CANX | calnexin | 1.642 | 3.73E-03 | 1.953 | 1.091 |
| 140_s_at | NM_004593 | TRA2B | transformer 2 beta homolog (Drosophila) | 1.641 | 1.22E-02 | 2.290 | 0.922 |
| 35804_at | NM_001105214; NM_004674 | ASH2L | ash2 (absent, small, or homeotic)-like (Drosophila) | 1.636 | 2.76E-02 | 1.837 | 1.456 |
| 1884_s_at | NM_002592; NM_182649 | PCNA | proliferating cell nuclear antigen | 1.634 | 4.86E-02 | 2.592 | 0.757 |
| 38075_at | NM_006754; NM_182715 | SYPL1 | EF-hand domain family, member A1 | 1.633 | 3.06E-02 | 2.859 | 0.787 |
| 36511_at | NM_014016 | SACM1L | SAC1 suppressor of actin mutations 1-like (yeast) | 1.631 | 4.27E-02 | 2.615 | 0.985 |
| 36821_at | NM_001010924 | FAM171A1 | family with sequence similarity 171, member A1 | 1.630 | 1.96E-02 | 2.349 | 0.785 |
| 1011_s_at | NM_006761; NR_024058 | YWHAE | tyrosine 3-monooxygenase/tryptophan 5-monooxygenase activation protein, epsilon polypeptide | 1.621 | 1.10E-02 | 1.715 | 1.531 |
| 35172_at | NM_001008566; NM_003595 | TPST2 | tyrosylprotein sulfotransferase 2 | 1.620 | 2.00E-02 | 1.786 | 1.470 |
| 35738_at | NM_006353 | HMGN4 | high mobility group nucleosomal binding domain 4 | 1.618 | 1.65E-02 | 2.238 | 1.087 |
| 38443_at | NM_002834 | PTPN11 | protein tyrosine phosphatase, non-receptor type 11 | 1.616 | 2.40E-02 | 1.791 | 0.966 |
| 38356_at | NM_006350; NM_013409 | FST | follistatin | 1.616 | 7.60E-03 | 1.697 | 1.538 |
| 34887_at | NM_002906 | RDX | radixin | 1.611 | 2.55E-02 | 2.000 | 1.129 |
| 34367_at | NM_006623 | PHGDH | phosphoglycerate dehydrogenase | 1.610 | 1.09E-03 | 1.790 | 1.755 |
| 38060_at | NM_001184979; NM_004552 | NDUFS5 | NADH dehydrogenase (ubiquinone) Fe-S protein 5, 15kDa (NADH-coenzyme Q reductase) | 1.607 | 3.25E-02 | 1.675 | 1.541 |
| 40068_at | NM_003164; XM_003120714 | LOC100510546; STX5 | syntaxin-5-like; syntaxin 5 | 1.606 | 1.82E-02 | 1.636 | 1.577 |
| 38485_at | NM_001184986; NM_001184987; NM_001184988; NM_001184989; NM_001184990; NM_001184991; NM_002494 | NDUFC1 | NADH dehydrogenase (ubiquinone) 1, subcomplex unknown, 1, 6kDa | 1.605 | 3.39E-02 | 2.130 | 0.787 |
| 32548_at | NM_006601 | PTGES3 | prostaglandin E synthase 3 (cytosolic) | 1.604 | 4.47E-02 | 2.113 | 1.260 |
| 37964_at | NM_006315 | PCGF3 | polycomb group ring finger 3 | 1.602 | 1.32E-02 | 1.731 | 1.483 |
| 37809_at | NM_018951; NM_152739; NM_153715 | HOXA10; HOXA9 | homeobox A10; homeobox A9 | 1.601 | 4.99E-02 | 1.903 | 1.148 |
| 949_s_at | NM_002806 | PSMC6 | proteasome (prosome, macropain) 26S subunit, ATPase, 6 | 1.597 | 1.49E-02 | 1.598 | 1.597 |
| 34819_at | NM_001142401; NM_001142402; NM_001142403; NM_001142404; NM_006016 | CD164 | CD164 molecule, sialomucin | 1.593 | 1.80E-02 | 1.994 | 1.019 |
| 32194_at | NM_005760 | CEBPZ | CCAAT/enhancer binding protein (C/EBP), zeta | 1.593 | 1.68E-02 | 1.679 | 1.511 |
| 38681_at | NM_001568 | EIF3E | eukaryotic translation initiation factor 3, subunit E | 1.591 | 6.94E-03 | 1.828 | 0.998 |
| 36631_at | NM_006793; NM_014098 | PRDX3 | peroxiredoxin 3 | 1.590 | 2.87E-02 | 2.646 | 0.935 |
| 41504_s_at | NM_001031804; NM_005360 | MAF | v-maf musculoaponeurotic fibrosarcoma oncogene homolog (avian) | 1.589 | 7.31E-04 | 1.590 | 1.589 |
| 32153_s_at | NM_018955 | UBB | ubiquitin B | 1.588 | 3.49E-02 | 1.797 | 1.403 |
| 39719_at | NM_001110354; NM_007155; NM_012230; NM_152992 | POMZP3; ZP3 | POM121 and ZP3 fusion; zona pellucida glycoprotein 3 (sperm receptor) | 1.587 | 3.94E-02 | 1.964 | 0.720 |
| 33351_at | NM_005875 | EIF1B | eukaryotic translation initiation factor 1B | 1.584 | 1.27E-02 | 1.665 | 0.994 |
| 41235_at | NM_001675; NM_182810 | ATF4 | activating transcription factor 4 (tax-responsive enhancer element B67) | 1.581 | 2.16E-02 | 1.946 | 1.011 |
| 36187_at | NM_002939; NM_203383; NM_203384; NM_203385; NM_203386; NM_203387; NM_203388; NM_203389 | RNH1 | ribonuclease/angiogenin inhibitor 1 | 1.579 | 4.78E-02 | 1.741 | 1.433 |
| 40112_at | NM_006899; NM_174855; NM_174856 | IDH3B | isocitrate dehydrogenase 3 (NAD+) beta | 1.579 | 1.27E-02 | 1.699 | 1.467 |
| 39517_at | NM_020199 | C5orf15 | chromosome 5 open reading frame 15 | 1.578 | 2.52E-02 | 1.590 | 1.565 |
| 37703_at | NM_004582 | RABGGTB | mitochondrial ribosomal protein L3 | 1.575 | 1.02E-02 | 1.844 | 1.186 |
| 37296_at | NM_001177 | ARL1 | ADP-ribosylation factor-like 1 | 1.574 | 1.20E-02 | 1.994 | 1.128 |
| 38820_at | NM_004261; NM_203341 | Sep 15 | 15 kDa selenoprotein | 1.573 | 7.62E-03 | 1.655 | 1.222 |
| 36636_at | NM_000274; NM_001171814 | OAT | ornithine aminotransferase | 1.572 | 4.24E-02 | 1.989 | 0.980 |
| 40421_at | NM_006221 | PIN1 | peptidylprolyl cis/trans isomerase, NIMA-interacting 1 | 1.571 | 3.53E-02 | 1.770 | 1.395 |
| 837_s_at | NM_002395 | ME1 | malic enzyme 1, NADP(+)-dependent, cytosolic | 1.571 | 3.33E-02 | 3.116 | 0.892 |
| 37035_at | NM_014445 | SERP1 | stress-associated endoplasmic reticulum protein 1 | 1.569 | 2.34E-02 | 1.940 | 1.007 |
| 39025_at | NM_001008393; NM_019059 | C4orf46; TOMM7 | chromosome 4 open reading frame 46; translocase of outer mitochondrial membrane 7 homolog (yeast) | 1.566 | 2.55E-02 | 1.614 | 1.520 |
| 39727_at | NM_003584 | DUSP11 | dual specificity phosphatase 11 (RNA/RNP complex 1-interacting) | 1.566 | 3.92E-02 | 2.566 | 0.992 |
| 38814_at | NM_004888 | ATP6V1G1 | ATPase, H+ transporting, lysosomal 13kDa, V1 subunit G1 | 1.564 | 2.19E-02 | 1.624 | 1.506 |
| 36913_at | NM_006527 | SLBP | stem-loop binding protein | 1.564 | 1.48E-02 | 1.820 | 1.065 |
| 33150_at | NM_020368 | UTP3 | UTP3, small subunit (SSU) processome component, homolog (S, cerevisiae) | 1.562 | 1.92E-02 | 1.633 | 1.494 |
| 33456_at | NM_012325 | MAPRE1 | microtubule-associated protein, RP/EB family, member 1 | 1.562 | 3.32E-02 | 2.473 | 0.793 |
| 32564_at | NM_006808 | SEC61B | Sec61 beta subunit | 1.560 | 4.47E-02 | 1.756 | 1.386 |
| 40522_at | NM_001033044; NM_001033056; NM_002065 | GLUL | glutamate-ammonia ligase | 1.558 | 3.27E-02 | 1.796 | 0.733 |
| 34814_at | NM_005499 | UBA2 | ubiquitin-like modifier activating enzyme 2 | 1.558 | 2.77E-02 | 2.265 | 0.949 |
| 32914_f_at | NM_001080543; NM_021231 | C19orf29 | chromosome 19 open reading frame 29 | 1.556 | 2.00E-02 | 1.572 | 1.540 |
| 38828_s_at | NM_003685 | KHSRP | KH-type splicing regulatory protein | 1.555 | 2.15E-02 | 1.649 | 1.467 |
| 34830_at | NM_030796 | VOPP1 | vesicular, overexpressed in cancer, prosurvival protein 1 | 1.551 | 2.63E-02 | 1.716 | 1.402 |
| 1102_s_at | NM_000176; NM_001018074; NM_001018075; NM_001018076; NM_001018077; NM_001020825; NM_001024094 | NR3C1 | nuclear receptor subfamily 3, group C, member 1 (glucocorticoid receptor) | 1.541 | 4.44E-02 | 1.734 | 1.427 |
| 38545_at | NM_002193 | INHBB | inhibin, beta B | 1.533 | 4.67E-02 | 1.721 | 1.366 |
| 38288_at | NM_003068 | SNAI2 | snail homolog 2 (Drosophila) | 1.532 | 3.91E-02 | 1.962 | 0.969 |
| 36102_at | NM_001135694; NM_005662 | VDAC3 | voltage-dependent anion channel 3 | 1.532 | 2.95E-02 | 1.683 | 1.395 |
| 36687_at | NM_001866 | COX7B | cytochrome c oxidase subunit VIIb | 1.531 | 4.00E-02 | 1.719 | 1.364 |
| 34340_at | NM_030579 | CYB5B | cytochrome b5 type B (outer mitochondrial membrane) | 1.531 | 2.10E-02 | 1.620 | 1.447 |
| 41335_at | NM_001042549; NM_015471 | NSL1 | NSL1, MIND kinetochore complex component, homolog (S, cerevisiae) | 1.529 | 4.84E-02 | 2.247 | 0.759 |
| 40638_at | NM_005066 | SFPQ | splicing factor proline/glutamine-rich | 1.528 | 3.02E-02 | 2.203 | 0.826 |
| 36515_at | NM_001128227; NM_001190383; NM_001190384; NM_001190388; NM_005476 | GNE | glucosamine (UDP-N-acetyl)-2-epimerase/N-acetylmannosamine kinase | 1.528 | 4.55E-02 | 1.744 | 1.339 |
| 39416_at | NM_014604 | TAX1BP3 | Tax1 (human T-cell leukemia virus type I) binding protein 3 | 1.526 | 1.03E-02 | 1.611 | 1.445 |
| 37337_at | NM_003096 | SNRPG | small nuclear ribonucleoprotein polypeptide G | 1.525 | 3.51E-02 | 1.862 | 0.807 |
| 36189_at | NM_004515 | ILF2 | interleukin enhancer binding factor 2, 45kDa | 1.518 | 5.78E-03 | 1.652 | 0.942 |
| 34299_at | NM_014323; NM_032050; NM_032051; NM_032052 | PATZ1 | POZ (BTB) and AT hook containing zinc finger 1 | 1.515 | 9.13E-03 | 1.560 | 1.470 |
| 39118_at | NM_001539 | DNAJA1 | DnaJ (Hsp40) homolog, subfamily A, member 1 | 1.514 | 4.82E-02 | 2.095 | 0.918 |
| 41448_at | NM_018951; NM_153715 | HOXA10 | homeobox A10 | 1.512 | 4.78E-04 | 1.615 | 1.409 |
| 38473_at | NM_152295 | TARS | threonyl-tRNA synthetase | 1.509 | 1.64E-02 | 1.816 | 0.985 |
| 40822_at | NM_004555; NM_173163; NM_173164; NM_173165 | NFATC3 | nuclear factor of activated T-cells, cytoplasmic, calcineurin-dependent 3 | 1.507 | 1.06E-02 | 1.599 | 1.420 |
| 33422_at | NM_001136232; NM_183352; NR_024272; NR_024273 | SEC13 | SEC13 homolog (S, cerevisiae) | 1.502 | 4.77E-02 | 1.698 | 1.328 |
| 36690_at | NM_000176; NM_001018074; NM_001018075; NM_001018076; NM_001018077; NM_001020825; NM_001024094 | NR3C1 | nuclear receptor subfamily 3, group C, member 1 (glucocorticoid receptor) | 1.502 | 1.72E-02 | 1.582 | 1.170 |

**Downregulated genes in the patient with AP-1 consensus sequence**

| **Probe Set ID** | **RefSeq Transcript ID** | **Gene Symbol** | **Gene Title** | **FC (abs)** | **p** | **min** | **max** |
| --- | --- | --- | --- | --- | --- | --- | --- |
| 33442_at | NM_015225 | PRUNE2 | prune homolog 2 (Drosophila) | 4.945 | 3.46E-02 | 3.312 | 7.384 |
| 32531_at | NM_000165 | GJA1 | gap junction protein, alpha 1, 43kDa | 3,905 | 1,91E-02 | 2.081 | 7.285 |
| 36892_at | NM_001144996; NM_001144997; NM_002206 | ITGA7 | integrin, alpha 7 | 3,555 | 6,36E-03 | 3.251 | 3.888 |
| 40422_at | NM_000597 | IGFBP2 | insulin-like growth factor binding protein 2, 36kDa | 3,2 | 1,89E-02 | 2.955 | 3.466 |
| 38026_at | NM_001996; NM_006485; NM_006486; NM_006487 | FBLN1 | fibulin 1 | 3,129 | 4,47E-02 | 2.992 | 3.273 |
| 115_at | NM_003246 | THBS1 | thrombospondin 1 | 2,938 | 1,61E-02 | 2.636 | 3.275 |
| 38750_at | NM_000435 | NOTCH3 | notch 3 | 2,884 | 9,85E-03 | 2.674 | 3.109 |
| 38508_s_at | NM_019105; NM_032470; NR_001284 | TNXA; TNXB | tenascin XA pseudogene; tenascin XB | 2,881 | 3,75E-02 | 2.292 | 3.622 |
| 32242_at | NM_001885 | CRYAB | crystallin, alpha B | 2,685 | 3,09E-02 | 1.795 | 4.044 |
| 36513_at | NM_003480 | MFAP5 | microfibrillar associated protein 5 | 2,611 | 2,01E-02 | 1.979 | 3.881 |
| 1741_s_at | NM_000597 | IGFBP2 | insulin-like growth factor binding protein 2, 36kDa | 2,567 | 2,89E-02 | 2.211 | 2.981 |
| 39101_at | NM_001100112; NM_017534 | MYH2 | myosin, heavy chain 2, skeletal muscle, adult | 2,498 | 4,02E-02 | 1.962 | 3.182 |
| 35289_at | NM_012197 | RABGAP1 | RAB GTPase activating protein 1 | 2,446 | 2,30E-02 | 2.061 | 2.903 |
| 32243_g_at | NM_001885 | CRYAB | crystallin, alpha B | 2,342 | 4,76E-02 | 1.635 | 3.570 |
| 37996_s_at | NM_001081560; NM_001081562; NM_001081563; NM_004409 | DMPK | dystrophia myotonica-protein kinase | 2,303 | 3,67E-02 | 1.953 | 2.714 |
| 36497_at | NM_138420 | AHNAK2 | AHNAK nucleoprotein 2 | 2,243 | 2,69E-02 | 1.693 | 3.245 |
| 39729_at | NM_005809; NM_181738 | PRDX2 | peroxiredoxin 2 | 2,235 | 6,73E-04 | 1.861 | 2.433 |
| 33764_at | NM_005458 | GABBR2 | gamma-aminobutyric acid (GABA) B receptor, 2 | 2,235 | 3,46E-02 | 1.473 | 2.726 |
| 38087_s_at | NM_002961; NM_019554 | S100A4 | S100 calcium binding protein A4 | 2,227 | 2,14E-02 | 1.551 | 2.756 |
| 35352_at | NM_014862 | ARNT2 | aryl-hydrocarbon receptor nuclear translocator 2 | 2,163 | 4,57E-04 | 1.879 | 2.428 |
| 38800_at | NM_007029 | STMN2 | stathmin-like 2 | 2,107 | 5,29E-03 | 2.107 | 2.107 |
| 40069_at | NM_003174; NM_021738 | SVIL | supervillin | 2,067 | 1,37E-02 | 2.064 | 2.070 |
| 1597_at | NM_000820; NM_001143945; NM_001143946 | GAS6 | growth arrest-specific 6 | 1,962 | 4,35E-02 | 1.445 | 2.782 |
| 1466_s_at | NM_002009 | FGF7 | fibroblast growth factor 7 | 1,957 | 1,29E-02 | 1.624 | 2.310 |
| 36136_at | NM_001076787; NM_006034 | TP53I11 | tumor protein p53 inducible protein 11 | 1,956 | 2,66E-02 | 1.674 | 2.287 |
| 38268_at | NM_004170 | SLC1A1 | solute carrier family 1 (neuronal/epithelial high affinity glutamate transporter, system Xag), member 1 | 1,95 | 3,76E-02 | 1.629 | 2.335 |
| 32612_at | NM_000177; NM_001127662; NM_001127663; NM_001127664; NM_001127665; NM_001127666; NM_001127667; NM_198252 | GSN | gelsolin | 1,898 | 2,47E-05 | 1.802 | 2.035 |
| 1005_at | NM_004417 | DUSP1 | dual specificity phosphatase 1 | 1,895 | 4,28E-02 | 1.566 | 2.293 |
| 692_s_at | NM_003102 | SOD3 | superoxide dismutase 3, extracellular | 1,873 | 2,18E-02 | 1.252 | 1.894 |
| 33410_at | NM_000210; NM_001079818 | ITGA6 | integrin, alpha 6 | 1,822 | 7,09E-03 | 1.768 | 1.878 |
| 33802_at | NM_002133 | HMOX1 | heme oxygenase (decycling) 1 | 1,774 | 1,14E-02 | 1.465 | 2.020 |
| 39754_at | NM_002213 | ITGB5 | integrin, beta 5 | 1,768 | 4,86E-02 | 1.510 | 2.197 |
| 39395_at | NM_006288 | THY1 | Thy-1 cell surface antigen | 1,75 | 1,19E-03 | 1.550 | 2.011 |
| 36070_at | NM_018689 | KIAA1199 | KIAA1199 | 1,75 | 4,31E-02 | 1.338 | 2.073 |
| 973_at | NM_001143676; NM_001143677; NM_001143678; NM_005627 | SGK1 | serum/glucocorticoid regulated kinase 1 | 1,744 | 3,98E-02 | 1.152 | 1.995 |
| 37765_at | NM_012134 | LMOD1 | leiomodin 1 (smooth muscle) | 1,728 | 5,73E-03 | 1.483 | 2.045 |
| 32667_at | NM_000495; NM_033380 | COL4A5 | collagen, type IV, alpha 5 | 1,724 | 2,04E-02 | 1.690 | 1.759 |
| 35717_at | NM_007168 | ABCA8 | ATP-binding cassette, sub-family A (ABC1), member 8 | 1,721 | 1,30E-02 | 1.696 | 1.747 |
| 2058_s_at | NM_002213 | ITGB5 | integrin, beta 5 | 1,717 | 3,49E-02 | 1.376 | 2.058 |
| 40455_at | NM_015036 | ENDOD1 | endonuclease domain containing 1 | 1,707 | 1,95E-02 | 1.605 | 1.814 |
| 39930_at | NM_004445 | EPHB6 | EPH receptor B6 | 1,705 | 1,42E-02 | 2.493 | 0.982 |
| 38267_at | NM_004170 | SLC1A1 | solute carrier family 1 (neuronal/epithelial high affinity glutamate transporter, system Xag), member 1 | 1,702 | 1,80E-02 | 1.551 | 1.868 |
| 672_at | NM_000602; NM_001165413 | SERPINE1 | serpin peptidase inhibitor, clade E (nexin, plasminogen activator inhibitor type 1), member 1 | 1,688 | 3,42E-02 | 3.351 | 1.126 |
| 40193_at | NM_001975 | ENO2 | enolase 2 (gamma, neuronal) | 1,686 | 3,38E-03 | 1.453 | 1.976 |
| 34836_at | NM_004583; NM_201434 | RAB5C | RAB5C, member RAS oncogene family | 1,685 | 2,82E-02 | 1.327 | 2.336 |
| 37621_at | NM_001190981; NM_002184; NM_175767 | IL6ST | interleukin 6 signal transducer (gp130, oncostatin M receptor) | 1,684 | 9,74E-03 | 1.638 | 1.732 |
| 36496_at | NM_014214 | IMPA2 | inositol(myo)-1(or 4)-monophosphatase 2 | 1,683 | 4,98E-03 | 1.363 | 1.727 |
| 36454_at | NM_001218; NM_206925 | CA12 | carbonic anhydrase XII | 1,645 | 1,83E-02 | 1.461 | 2.065 |
| 1495_at | NM_000627; NM_001166264; NM_001166265; NM_001166266; NM_206943 | LTBP1 | latent transforming growth factor beta binding protein 1 | 1,641 | 3,95E-02 | 1.342 | 2.013 |
| 1220_g_at | NM_002199 | IRF2 | interferon regulatory factor 2 | 1,635 | 4,50E-02 | 1.432 | 1.866 |
| 39593_at | NM_006682 | FGL2 | fibrinogen-like 2 | 1,633 | 1,60E-02 | 1.586 | 1.682 |
| 1530_g_at | NM_023037 | FRY | furry homolog (Drosophila) | 1,63 | 1,45E-02 | 1.524 | 1.744 |
| 34265_at | NM_001144757; NM_003020 | SCG5 | secretogranin V (7B2 protein) | 1,627 | 1,45E-02 | 1.438 | 2.001 |
| 37542_at | NM_005779 | LHFPL2 | lipoma HMGIC fusion partner-like 2 | 1,615 | 8,35E-03 | 1.463 | 1.821 |
| 33878_at | NM_025202; NR_027663 | EFHD1 | EF-hand domain family, member D1 | 1,604 | 2,72E-02 | 1.457 | 1.766 |
| 33088_at | NM_002238; NM_172362 | KCNH1 | potassium voltage-gated channel, subfamily H (eag-related), member 1 | 1,59 | 3,75E-02 | 1.417 | 1.785 |
| 31983_at | NM_001165924; NM_004098 | EMX2 | empty spiracles homeobox 2 | 1,588 | 1,79E-02 | 1.565 | 1.611 |
| 32313_at | NM_003289; NM_213674 | TPM2 | tropomyosin 2 (beta) | 1,58 | 2,34E-02 | 1.396 | 1.932 |
| 39616_at | NM_001126044; NM_198714; NM_198715; NM_198716; NM_198717; NM_198718; NM_198719; NR_028292; NR_028293; NR_028294 | PTGER3 | prostaglandin E receptor 3 (subtype EP3) | 1,578 | 2,48E-02 | 1.525 | 1.634 |
| 38127_at | NM_001006946; NM_002997 | SDC1 | NADH dehydrogenase (ubiquinone) Fe-S protein 8, 23kDa (NADH-coenzyme Q reductase) | 1,577 | 9,85E-03 | 2.221 | 1.201 |
| 36644_at | NM_001039490; NM_004357; NM_139029; NM_139030 | CD151 | CD151 molecule (Raph blood group) | 1,57 | 4,11E-02 | 1.138 | 1.835 |
| 422_s_at | NM_002382; NM_145112; NM_145113; NM_145114; NM_145116; NM_197957 | MAX | MYC associated factor X | 1,564 | 3,81E-02 | 1.466 | 1.669 |
| 32314_g_at | NM_003289; NM_213674 | TPM2 | tropomyosin 2 (beta) | 1,562 | 4,88E-02 | 1.355 | 1.800 |
| 36042_at | NM_001007097; NM_001018064; NM_001018065; NM_001018066; NM_006180 | NTRK2 | neurotrophic tyrosine kinase, receptor, type 2 | 1,556 | 3,76E-03 | 1.553 | 1.560 |
| 32740_at | NM_014904 | RAB11FIP2 | RAB11 family interacting protein 2 (class I) | 1,554 | 1,91E-02 | 2.304 | 1.205 |
| 36728_at | NM_000678 | ADRA1D | adrenergic, alpha-1D-, receptor | 1,546 | 4,19E-02 | 1.446 | 1.653 |
| 34866_at | NM_002410; NR_024275 | LOC151162; MGAT5 | hypothetical LOC151162; mannosyl (alpha-1,6-)-glycoprotein beta-1,6-N-acetyl-glucosaminyltransferase | 1,542 | 3,46E-02 | 1.268 | 2.177 |
| 37641_at | NM_006417 | IFI44 | interferon-induced protein 44 | 1,527 | 2,76E-02 | 1.383 | 1.687 |
| 39422_at | NM_001002810; NM_001002811; NM_001002812; NM_001195260; NM_001195261; NM_001198832; NM_001198834; NM_014644; NM_022359 | PDE4DIP | phosphodiesterase 4D interacting protein | 1,527 | 7,72E-03 | 1.387 | 1.615 |
| 31856_at | NM_001128922; NM_005512 | LRRC32 | leucine rich repeat containing 32 | 1,515 | 2,77E-03 | 2.311 | 1.181 |
| 39058_at | NM_001092; NM_001159746; NM_021962 | ABR | active BCR-related gene | 1,514 | 3,69E-03 | 1.384 | 1.754 |
| 885_g_at | NM_002204; NM_005501 | ITGA3 | integrin, alpha 3 (antigen CD49C, alpha 3 subunit of VLA-3 receptor) | 1,512 | 8,33E-04 | 1.625 | 1.228 |
| 35871_s_at | NM_001098484; NM_001134742; NM_003759 | SLC4A4 | solute carrier family 4, sodium bicarbonate cotransporter, member 4 | 1,505 | 4,83E-02 | 1.445 | 1.568 |

**Downregulated genes in the patient without AP-1 consensus sequence**

| **Probe Set ID** | **RefSeq Transcript ID** | **Gene Symbol** | **Gene Title** | **FC (abs)** | **p** | **min** | **max** |
| --- | --- | --- | --- | --- | --- | --- | --- |
| 39206_s_at | NM_001135; NM_013227 | ACAN | aggrecan | 22.093 | 3.79E-04 | 20.289 | 24.057 |
| 38965_at | NM_001135; NM_013227 | ACAN | aggrecan | 9.388 | 4.03E-03 | 8.189 | 10.762 |
| 39207_r_at | NM_001135; NM_013227 | ACAN | aggrecan | 5.361 | 3.20E-03 | 5.209 | 5.517 |
| 38965_at | NM_001135; NM_013227 | ACAN | aggrecan | 4.912 | 4.51E-03 | 10.762 | 1.400 |
| 37892_at | NM_001190709; NM_001854; NM_080629; NM_080630 | COL11A1 | collagen, type XI, alpha 1 | 3,959 | 2,48E-02 | 3.165 | 4.952 |
| 35303_at | NM_005542; NM_198336; NM_198337 | INSIG1 | insulin induced gene 1 | 3,527 | 1,35E-02 | 1.544 | 2.294 |
| 34203_at | NM_001299 | CNN1 | calponin 1, basic, smooth muscle | 3,033 | 2,10E-03 | 2.394 | 3.624 |
| 34403_at | NM_001114614; NM_005928 | MFGE8 | milk fat globule-EGF factor 8 protein | 2,947 | 1,93E-02 | 2.893 | 3.003 |
| 37399_at | NM_003739 | AKR1C3 | aldo-keto reductase family 1, member C3 (3-alpha hydroxysteroid dehydrogenase, type II) | 2,549 | 4,17E-02 | 1.555 | 4.482 |
| 32814_at | NM_001548 | IFIT1 | interferon-induced protein with tetratricopeptide repeats 1 | 2,498 | 1,13E-02 | 2.272 | 2.746 |
| 32521_at | NM_003012 | SFRP1 | secreted frizzled-related protein 1 | 2,478 | 4,88E-02 | 1.694 | 4.995 |
| 915_at | NM_001548 | IFIT1 | interferon-induced protein with tetratricopeptide repeats 1 | 2,461 | 2,88E-03 | 2.322 | 2.609 |
| 37014_at | NM_001144925; NM_001178046; NM_002462 | MX1 | myxovirus (influenza virus) resistance 1, interferon-inducible protein p78 (mouse) | 2,149 | 1,39E-02 | 1.889 | 2.443 |
| 37512_at | NM_003725 | HSD17B6 | hydroxysteroid (17-beta) dehydrogenase 6 homolog (mouse) | 2,114 | 2,20E-02 | 1.823 | 2.453 |
| 875_g_at | NM_002982 | CCL2 | chemokine (C-C motif) ligand 2 | 2,013 | 2,96E-02 | 1.401 | 2.576 |
| 37399_at | NM_003739 | AKR1C3 | aldo-keto reductase family 1, member C3 (3-alpha hydroxysteroid dehydrogenase, type II) | 1,978 | 1,25E-02 | 4.323 | 1.612 |
| 434_at | NM_005318 | H1F0 | H1 histone family, member 0 | 1,95 | 2,35E-02 | 1.706 | 2.230 |
| 38446_at | NR_001564 | XIST | X (inactive)-specific transcript (non-protein coding) | 1,899 | 4,12E-02 | 1.623 | 2.221 |
| 353_at | NM_012399 | PITPNB | phosphatidylinositol transfer protein, beta | 1,882 | 4,64E-02 | 1.727 | 2.266 |
| 1197_at | NM_001615 | ACTG2 | actin, gamma 2, smooth muscle, enteric | 1,823 | 1,34E-02 | 1.351 | 1.748 |
| 2062_at | NM_001553 | IGFBP7 | insulin-like growth factor binding protein 7 | 1,797 | 3,76E-02 | 3.307 | 0.909 |
| 31557_at | NM_021109 | TMSB4X | thymosin beta 4, X-linked | 1,712 | 7,98E-05 | 1.620 | 1.815 |
| 1221_at | NM_002868 | RAB5B | RAB5B, member RAS oncogene family | 1,699 | 3,72E-03 | 1.629 | 1.772 |
| 39207_r_at | NM_001135; NM_013227 | ACAN | aggrecan | 1,687 | 4,27E-02 | 1.368 | 2.097 |
| 32675_at | NM_004334 | BST1 | bone marrow stromal cell antigen 1 | 1,676 | 4,58E-02 | 1.338 | 2.131 |
| 34069_s_at | NM_001007559; NM_003147; NM_005637; NM_175698 | SS18; SSX2 | synovial sarcoma translocation, chromosome 18; synovial sarcoma, X breakpoint 2 | 1,668 | 9,54E-03 | 1.556 | 1.789 |
| 34797_at | NM_003711; NM_176895 | PPAP2A | phosphatidic acid phosphatase type 2A | 1,659 | 4,41E-02 | 1.330 | 2.118 |
| 37678_at | NM_012342 | BAMBI | interferon-related developmental regulator 1 | 1,63 | 4,63E-03 | 2.676 | 1.263 |
| 39695_at | NM_000574; NM_001114752 | CD55 | CD55 molecule, decay accelerating factor for complement (Cromer blood group) | 1,616 | 1,50E-02 | 1.436 | 2.046 |
| 920_at | NM_005385 | NKTR | natural killer-tumor recognition sequence | 1,613 | 2,76E-02 | 1.438 | 1.808 |
| 38503_at | NM_000692 | ALDH1B1 | aldehyde dehydrogenase 1 family, member B1 | 1,586 | 1,56E-02 | 1.518 | 1.657 |
| 39145_at | NM_006097; NM_181526 | MYL9 | myosin, light chain 9, regulatory | 1,562 | 5,67E-04 | 1.511 | 1.598 |
| 35961_at |  | --- | --- | 1,558 | 1,80E-02 | 1.432 | 1.694 |
| 37512_at | NM_003725 | HSD17B6 | hydroxysteroid (17-beta) dehydrogenase 6 homolog (mouse) | 1,556 | 3,11E-02 | 2.453 | 0.774 |
| 39170_at | NM_000611; NM_001127223; NM_001127225; NM_001127226; NM_001127227; NM_203329; NM_203330; NM_203331 | CD59 | CD59 molecule, complement regulatory protein | 1,545 | 3,94E-03 | 1.462 | 1.729 |
| 34375_at | NM_002982 | CCL2 | chemokine (C-C motif) ligand 2 | 1,544 | 1,04E-02 | 2.410 | 1.039 |
| 34512_at | NM_000683 | ADRA2C | adrenergic, alpha-2C-, receptor | 1,521 | 4,70E-02 | 1.360 | 1.702 |
| 34012_at | NM_021013 | KRT34 | keratin 34 | 1,511 | 3,47E-02 | 1.427 | 1.600 |
| 531_at | NM_006851 | GLIPR1 | GLI pathogenesis-related 1 | 1,51 | 3,85E-02 | 1.873 | 1.068 |
| 35674_at | NM_007365 | PADI2 | peptidyl arginine deiminase, type II | 1,507 | 3,19E-02 | 1.372 | 1.656 |
| 40776_at | NM_001927 | DES | desmin | 1,5 | 4,39E-02 | 1.325 | 1.699 |
