# Supplementary material for: A mutation in the c-Fos gene associated with congenital generalized lipodystrophy
Source: Orphanet J Rare Dis. 2013 Aug 7;8:119. doi: 10.1186/1750-1172-8-119 (PMC3750569; doi:10.1186/1750-1172-8-119)
Supplement: Additional file 2: Table S2 — GO annotation of regulated transcripts. Transcripts are listed in identical order as in Additional file 1: Table S1. Functional information is given as available. For consensus site prediction web based tools as http://david.abcc.ncifcrf.gov/ were used. [file 1750-1172-8-119-S2.docx]

**Supplement Table 2: GO annotation of regulated transcripts**

Transcripts are listed in identical order as in Supplement table 2. Functional information is given as available. For consensus site prediction web based tools as http://david.abcc.ncifcrf.gov/ were used.

**Upregulated genes in the patient containing an AP-1 consensus sequence**

| **Probe Set ID** | **Gene Symbol** | **Gene Ontology Biological Process** |
| --- | --- | --- |
| 31862_at | WNT5A | 0000187: activation of MAPK activity; 0001667: ameboidal cell migration; 0001736: establishment of planar polarity; 0001756: somitogenesis; 0001837: epithelial to mesenchymal transition; 0001843: neural tube closure; 0001934: positive regulation of protein phosphorylation; 0001938: positive regulation of endothelial cell proliferation; 0001947: heart looping; 0002009: morphogenesis of an epithelium; 0002053: positive regulation of mesenchymal cell proliferation; 0002088: lens development in camera-type eye; 0002741: positive regulation of cytokine secretion involved in immune response; 0003323: pancreatic B cell development; 0003344: pericardium morphogenesis; 0003401: axis elongation; 0003408: optic cup formation involved in camera-type eye development; 0006468: protein phosphorylation; 0007223: Wnt receptor signaling pathway, calcium modulating pathway; 0007254: JNK cascade; 0007257: activation of JUN kinase activity; 0007275: multicellular organismal development; 0007442: hindgut morphogenesis; 0007494: midgut development; 0008284: positive regulation of cell proliferation; 0008584: male gonad development; 0009790: embryo development; 0009948: anterior/posterior axis specification; 0009952: anterior/posterior pattern formation; 0010551: regulation of gene-specific transcription from RNA polymerase II promoter; 0010595: positive regulation of endothelial cell migration; 0010800: positive regulation of peptidyl-threonine phosphorylation; 0010820: positive regulation of T cell chemotaxis; 0016055: Wnt receptor signaling pathway; 0016477: cell migration; 0021915: neural tube development; 0030154: cell differentiation; 0030182: neuron differentiation; 0030216: keratinocyte differentiation; 0030324: lung development; 0030326: embryonic limb morphogenesis; 0030514: negative regulation of BMP signaling pathway; 0030825: positive regulation of cGMP metabolic process; 0032148: activation of protein kinase B activity; 0032582: negative regulation of gene-specific transcription; 0033138: positive regulation of peptidyl-serine phosphorylation; 0034613: cellular protein localization; 0034613: cellular protein localization; 0035108: limb morphogenesis; 0035121: tail morphogenesis; 0040037: negative regulation of fibroblast growth factor receptor signaling pathway; 0042060: wound healing; 0042472: inner ear morphogenesis; 0042733: embryonic digit morphogenesis; 0043066: negative regulation of apoptosis; 0043507: positive regulation of JUN kinase activity; 0045080: positive regulation of chemokine biosynthetic process; 0045599: negative regulation of fat cell differentiation; 0045732: positive regulation of protein catabolic process; 0045766: positive regulation of angiogenesis; 0045778: positive regulation of ossification; 0045836: positive regulation of meiosis; 0045941: positive regulation of transcription; 0046330: positive regulation of JNK cascade; 0048146: positive regulation of fibroblast proliferation; 0048546: digestive tract morphogenesis; 0048706: embryonic skeletal system development; 0048806: genitalia development; 0048843: negative regulation of axon extension involved in axon guidance; 0048850: hypophysis morphogenesis; 0050679: positive regulation of epithelial cell proliferation; 0050680: negative regulation of epithelial cell proliferation; 0050919: negative chemotaxis; 0051092: positive regulation of NF-kappaB transcription factor activity; 0051216: cartilage development; 0060021: palate development; 0060026: convergent extension; 0060029: convergent extension involved in organogenesis; 0060065: uterus development; 0060067: cervix development; 0060068: vagina development; 0060070: canonical Wnt receptor signaling pathway 0060071: Wnt receptor signaling pathway, planar cell polarity pathway; 0060157: urinary bladder development; 0060324: face development; 0060340: positive regulation of type I interferon-mediated signaling pathway; 0060541: respiratory system development 0060599: lateral sprouting involved in mammary gland duct morphogenesis; 0060606: tube closure; 0060638: mesenchymal-epithelial cell signaling; 0060686: negative regulation of prostatic bud formation; 0060744: mammary gland branching involved in the larche 0060750: epithelial cell proliferation involved in mammary gland duct elongation; 0060760: positive regulation of response to cytokine stimulus; 0060762: regulation of branching involved in mammary gland duct morphogenesis; 0060907: positive regulation of macrophage cytokine production; 0061036: positive regulation of cartilage development; 0070245: positive regulation of thymocyte apoptosis; 0071222: cellular response to lipopolysaccharide; 0071277: cellular response to calcium ion; 0071300: cellular response to retinoic acid; 0071346: cellular response to interferon-gamma; 0071425: hemopoietic stem cell proliferation; 0071542: dopaminergic neuron differentiation; 0071560: cellular response to transforming growth factor beta stimulus; 0072201: negative regulation of mesenchymal cell proliferation; 0090009: primitive streak formation; 0090037: positive regulation of protein kinase C signaling; 0090090: negative regulation of canonical Wnt receptor signaling pathway; 0090103: cochlea morphogenesis |
| 38650_at | IGFBP5 | 0001558: regulation of cell growth; 0001649: osteoblast differentiation; 0007165: signal transduction; 0014912: negative regulation of smooth muscle cell migration; 0017148: negative regulation of translation; 0030336: negative regulation of cell migration; 0043569: negative regulation of insulin-like growth factor receptor signaling pathway; 0048630: skeletal muscle tissue growth; 0048662: negative regulation of smooth muscle cell proliferation |
| 32186_at | SLC7A5 | 0006520: cellular amino acid metabolic process; 0006810: transport; 0006865: amino acid transport; 0007275: multicellular organismal development; 0007399: nervous system development; 0015804: neutral amino acid transport; 0030154: cell differentiation; 0055085: transmembrane transport |
| 1396_at | IGFBP5 | 0001558: regulation of cell growth; 0001649: osteoblast differentiation; 0007165: signal transduction; 0014912: negative regulation of smooth muscle cell migration; 0017148: negative regulation of translation; 0030336: negative regulation of cell migration; 0043569: negative regulation of insulin-like growth factor receptor signaling pathway; 0048630: skeletal muscle tissue growth; 0048662: negative regulation of smooth muscle cell proliferation |
| 34778_at | LRRC15 | 0016020: membrane; 0016021: integral to membrane |
| 36543_at | F3 | 0001938: positive regulation of endothelial cell proliferation; 0002541: activation of plasma proteins involved in acute inflammatory response; 0002543: activation of blood coagulation via clotting cascade; 0006916: anti-apoptosis; 0006919: activation of caspase activity; 0007568: aging; 0007596: blood coagulation; 0007598: blood coagulation, extrinsic pathway; 0009266: response to temperature stimulus; 0009611: response to wounding; 0009612: response to mechanical stimulus; 0010641: positive regulation of platelet-derived growth factor receptor signaling pathway; 0014911: positive regulation of smooth muscle cell migration; 0030335: positive regulation of cell migration; 0032355: response to estradiol stimulus; 0032496: response to lipopolysaccharide; 0034405: response to fluid shear stress; 0045766: positive regulation of angiogenesis; 0050927: positive regulation of positive chemotaxis; 0051897: positive regulation of protein kinase B signaling cascade; 0055098: response to low-density lipoprotein stimulus; 0070301: cellular response to hydrogen peroxide |
| 38299_at | IL6 | 0001781: neutrophil apoptosis; 0002384: hepatic immune response; 0002384: hepatic immune response; 0002446: neutrophil mediated immunity; 0002548: monocyte chemotaxis; 0002675: positive regulation of acute inflammatory response; 0002690: positive regulation of leukocyte chemotaxis; 0006469: negative regulation of protein kinase activity; 0006953: acute-phase response; 0006953: acute-phase response; 0006954: inflammatory response; 0006955: immune response; 0006959: humoral immune response; 0007568: aging; 0008284: positive regulation of cell proliferation; 0008284: positive regulation of cell proliferation; 0008285: negative regulation of cell proliferation; 0008360: regulation of cell shape; 0009408: response to heat; 0009409: response to cold; 0009611: response to wounding; 0009612: response to mechanical stimulus; 0009617: response to bacterium; 0010574: regulation of vascular endothelial growth factor production; 0010628: positive regulation of gene expression; 0010888: negative regulation of lipid storage; 0014070: response to organic cyclic substance; 0019221: cytokine-mediated signaling pathway; 0030168: platelet activation; 0031000: response to caffeine; 0031018: endocrine pancreas development; 0031175: neuron projection development; 0031667: response to nutrient levels; 0032494: response to peptidoglycan; 0032496: response to lipopolysaccharide; 0032722: positive regulation of chemokine production; 0032755: positive regulation of interleukin-6 production; 0032868: response to insulin stimulus; 0032966: negative regulation of collagen biosynthetic process; 0033138: positive regulation of peptidyl-serine phosphorylation; 0033160: positive regulation of protein import into nucleus, translocation; 0034097: response to cytokine stimulus; 0042102: positive regulation of T cell proliferation; 0042127: regulation of cell proliferation; 0042493: response to drug; 0042517: positive regulation of tyrosine phosphorylation of Stat3 protein; 0042832: defense response to protozoan; 0042981: regulation of apoptosis; 0042981: regulation of apoptosis; 0043066: negative regulation of apoptosis; 0043154: negative regulation of caspase activity; 0043193: positive regulation of gene-specific transcription; 0043200: response to amino acid stimulus; 0043410: positive regulation of MAPKKK cascade; 0043526: neuroprotection; 0045079: negative regulation of chemokine biosynthetic process; 0045079: negative regulation of chemokine biosynthetic process; 0045188: regulation of circadian sleep/wake cycle, non-REM sleep; 0045429: positive regulation of nitric oxide biosynthetic process; 0045454: cell redox homeostasis; 0045599: negative regulation of fat cell differentiation; 0045630: positive regulation of T-helper 2 cell differentiation; 0045666: positive regulation of neuron differentiation; 0045669: positive regulation of osteoblast differentiation; 0045721: negative regulation of gluconeogenesis; 0045727: positive regulation of translation; 0045740: positive regulation of DNA replication; 0045765: regulation of angiogenesis; 0045768: positive regulation of anti-apoptosis; 0045893: positive regulation of transcription, DNA-dependent; 0045944: positive regulation of transcription from RNA polymerase II promoter; 0046427: positive regulation of JAK-STAT cascade; 0046677: response to antibiotic; 0046716: muscle cell homeostasis; 0046849: bone remodeling; 0046888: negative regulation of hormone secretion; 0048635: negative regulation of muscle organ development; 0048661: positive regulation of smooth muscle cell proliferation; 0050679: positive regulation of epithelial cell proliferation; 0050710: negative regulation of cytokine secretion; 0050731: positive regulation of peptidyl-tyrosine phosphorylation; 0050829: defense response to Gram-negative bacterium; 0050830: defense response to Gram-positive bacterium; 0050871: positive regulation of B cell activation; 0051024: positive regulation of immunoglobulin secretion; 0051091: positive regulation of transcription factor activity; 0051092: positive regulation of NF-kappaB transcription factor activity; 0051384: response to glucocorticoid stimulus; 0051592: response to calcium ion; 0051602: response to electrical stimulus; 0051607: defense response to virus; 0051789: response to protein stimulus; 0051971: positive regulation of transmission of nerve impulse; 0060445: branching involved in salivary gland morphogenesis; 0060664: epithelial cell proliferation involved in salivary gland morphogenesis; 0070091: glucagon secretion; 0070102: interleukin-6-mediated signaling pathway; 0070374: positive regulation of ERK1 and ERK2 cascade |
| 659_g_at | THBS2 | 0007155: cell adhesion |
| 581_at | LAMB1 | 0007155: cell adhesion; 0007566: embryo implantation; 0030335: positive regulation of cell migration; 0031175: neuron projection development; 0031175: neuron projection development; 0034446: substrate adhesion-dependent cell spreading; 0042476: odontogenesis; 0050679: positive regulation of epithelial cell proliferation |
| 38427_at | COL15A1 | 0001525: angiogenesis; 0007155: cell adhesion; 0007165: signal transduction; 0007275: multicellular organismal development; 0030154: cell differentiation |
| 1669_at | WNT5A | 0000187: activation of MAPK activity; 0001667: ameboidal cell migration; 0001736: establishment of planar polarity; 0001756: somitogenesis; 0001837: epithelial to mesenchymal transition; 0001843: neural tube closure; 0001934: positive regulation of protein phosphorylation; 0001938: positive regulation of endothelial cell proliferation; 0001947: heart looping; 0002009: morphogenesis of an epithelium; 0002053: positive regulation of mesenchymal cell proliferation; 0002088: lens development in camera-type eye; 0002741: positive regulation of cytokine secretion involved in immune response; 0003323: pancreatic B cell development; 0003344: pericardium morphogenesis; 0003401: axis elongation; 0003408: optic cup formation involved in camera-type eye development; 0006468: protein phosphorylation; 0007223: Wnt receptor signaling pathway, calcium modulating pathway; 0007254: JNK cascade; 0007257: activation of JUN kinase activity; 0007275: multicellular organismal development; 0007442: hindgut morphogenesis; 0007494: midgut development; 0008284: positive regulation of cell proliferation; 0008584: male gonad development; 0009790: embryo development; 0009948: anterior/posterior axis specification; 0009952: anterior/posterior pattern formation; 0010551: regulation of gene-specific transcription from RNA polymerase II promoter; 0010595: positive regulation of endothelial cell migration; 0010800: positive regulation of peptidyl-threonine phosphorylation; 0010820: positive regulation of T cell chemotaxis; 0016055: Wnt receptor signaling pathway; 0016477: cell migration; 0021915: neural tube development; 0030154: cell differentiation; 0030182: neuron differentiation; 0030216: keratinocyte differentiation; 0030324: lung development; 0030326: embryonic limb morphogenesis; 0030514: negative regulation of BMP signaling pathway; 0030825: positive regulation of cGMP metabolic process; 0032148: activation of protein kinase B activity; 0032582: negative regulation of gene-specific transcription; 0033138: positive regulation of peptidyl-serine phosphorylation; 0034613: cellular protein localization; 0034613: cellular protein localization; 0035108: limb morphogenesis; 0035121: tail morphogenesis; 0040037: negative regulation of fibroblast growth factor receptor signaling pathway; 0042060: wound healing; 0042472: inner ear morphogenesis; 0042733: embryonic digit morphogenesis; 0043066: negative regulation of apoptosis; 0043507: positive regulation of JUN kinase activity; 0045080: positive regulation of chemokine biosynthetic process; 0045599: negative regulation of fat cell differentiation; 0045732: positive regulation of protein catabolic process; 0045766: positive regulation of angiogenesis; 0045778: positive regulation of ossification; 0045836: positive regulation of meiosis; 0045941: positive regulation of transcription; 0046330: positive regulation of JNK cascade; 0048146: positive regulation of fibroblast proliferation; 0048546: digestive tract morphogenesis; 0048706: embryonic skeletal system development; 0048806: genitalia development; 0048843: negative regulation of axon extension involved in axon guidance; 0048850: hypophysis morphogenesis; 0050679: positive regulation of epithelial cell proliferation; 0050680: negative regulation of epithelial cell proliferation; 0050919: negative chemotaxis; 0051092: positive regulation of NF-kappaB transcription factor activity; 0051216: cartilage development; 0060021: palate development; 0060026: convergent extension; 0060029: convergent extension involved in organogenesis; 0060065: uterus development; 0060067: cervix development; 0060068: vagina development; 0060070: canonical Wnt receptor signaling pathway 0060071: Wnt receptor signaling pathway, planar cell polarity pathway; 0060157: urinary bladder development; 0060324: face development; 0060340: positive regulation of type I interferon-mediated signaling pathway; 0060541: respiratory system development 0060599: lateral sprouting involved in mammary gland duct morphogenesis; 0060606: tube closure; 0060638: mesenchymal-epithelial cell signaling; 0060686: negative regulation of prostatic bud formation; 0060744: mammary gland branching involved in the larche 0060750: epithelial cell proliferation involved in mammary gland duct elongation; 0060760: positive regulation of response to cytokine stimulus; 0060762: regulation of branching involved in mammary gland duct morphogenesis; 0060907: positive regulation of macrophage cytokine production; 0061036: positive regulation of cartilage development; 0070245: positive regulation of thymocyte apoptosis; 0071222: cellular response to lipopolysaccharide; 0071277: cellular response to calcium ion; 0071300: cellular response to retinoic acid; 0071346: cellular response to interferon-gamma; 0071425: hemopoietic stem cell proliferation; 0071542: dopaminergic neuron differentiation; 0071560: cellular response to transforming growth factor beta stimulus; 0072201: negative regulation of mesenchymal cell proliferation; 0090009: primitive streak formation; 0090037: positive regulation of protein kinase C signaling; 0090090: negative regulation of canonical Wnt receptor signaling pathway; 0090103: cochlea morphogenesis |
| 39827_at | DDIT4 | 0001666: response to hypoxia; 0006915: apoptosis; 0009968: negative regulation of signal transduction |
| 33127_at | LOXL2 | 0006464: protein modification process; 0007155: cell adhesion; 0007568: aging; 0055114: oxidation reduction |
| 2086_s_at | TYRO3 | 0006468: protein phosphorylation; 0007155: cell adhesion; 0007165: signal transduction |
| 33369_at | SC4MOL | 0006631: fatty acid metabolic process; 0006633: fatty acid biosynthetic process; 0006694: steroid biosynthetic process; 0008202: steroid metabolic process; 0008610: lipid biosynthetic process; 0016126: sterol biosynthetic process; 0055114: oxidation reduction |
| 658_at | THBS2 | 0007155: cell adhesion |
| 34335_at | EFNB2 | 0001945: lymph vessel development; 0007267: cell-cell signaling; 0007275: multicellular organismal development; 0007399: nervous system development; 0009653: anatomical structure morphogenesis; 0009887: organ morphogenesis; 0030154: cell differentiation; 0044419: interspecies interaction between organisms |
| 36658_at | DHCR24 | 0006694: steroid biosynthetic process; 0006695: cholesterol biosynthetic process; 0006915: apoptosis; 0006916: anti-apoptosis; 0006979: response to oxidative stress; 0007050: cell cycle arrest; 0007265: Ras protein signal transduction; 0008104: protein localization; 0008203: cholesterol metabolic process; 0008285: negative regulation of cell proliferation; 0008610: lipid biosynthetic process; 0009888: tissue development; 0016044: cellular membrane organization; 0016125: sterol metabolic process; 0016126: sterol biosynthetic process; 0030539: male genitalia development; 0031639: plasminogen activation; 0042987: amyloid precursor protein catabolic process; 0043154: negative regulation of caspase activity; 0043526: neuroprotection; 0043588: skin development; 0055114: oxidation reduction |
| 39127_f_at | PPP2R4 | 0032515: negative regulation of phosphoprotein phosphatase activity; 0032516: positive regulation of phosphoprotein phosphatase activity; 0035307: positive regulation of protein dephosphorylation; 0035308: negative regulation of protein dephosphorylation; 0043065: positive regulation of apoptosis; 0043666: regulation of phosphoprotein phosphatase activity |
| 133_at | CTSC | 0006508: proteolysis; 0006955: immune response; 0007568: aging; 0010033: response to organic substance |
| 35829_at | CADM1 | 0001889: liver development; 0001913: T cell mediated cytotoxicity; 0006915: apoptosis; 0007155: cell adhesion; 0007156: homophilic cell adhesion; 0007157: heterophilic cell-cell adhesion; 0007275: multicellular organismal development; 0007283: spermatogenesis; 0008037: cell recognition; 0030154: cell differentiation; 0042271: susceptibility to natural killer cell mediated cytotoxicity; 0045954: positive regulation of natural killer cell mediated cytotoxicity; 0050715: positive regulation of cytokine secretion; 0050798: activated T cell proliferation; 0051606: detection of stimulus |
| 41778_at | SLC1A5 | 0006810: transport; 0006835: dicarboxylic acid transport; 0006865: amino acid transport; 0015804: neutral amino acid transport |
| 34849_at | SARS | 0006412: translation; 0006418: tRNA aminoacylation for protein translation; 0006434: seryl-tRNA aminoacylation; 0008033: tRNA processing |
| 38098_at | LPIN1 | 0006350: transcription; 0006629: lipid metabolic process; 0006631: fatty acid metabolic process; 0006642: triglyceride mobilization; 0009062: fatty acid catabolic process; 0019432: triglyceride biosynthetic process; 0031100: organ regeneration; 0031529: ruffle organization; 0031532: actin cytoskeleton reorganization; 0032869: cellular response to insulin stimulus; 0045449: regulation of transcription; 0045598: regulation of fat cell differentiation; 0045944: positive regulation of transcription from RNA polymerase II promoter |
| 40408_at | CARS | 0006412: translation; 0006418: tRNA aminoacylation for protein translation; 0006423: cysteinyl-tRNA aminoacylation; 0006423: cysteinyl-tRNA aminoacylation |
| 38077_at | COL6A3 | 0007155: cell adhesion; 0007517: muscle organ development |
| 32168_s_at | RCAN1 | 0007165: signal transduction; 0007417: central nervous system development; 0008015: blood circulation; 0019722: calcium-mediated signaling; 0043666: regulation of phosphoprotein phosphatase activity; 0048741: skeletal muscle fiber development |
| 37188_at | PCK2 | 0006094: gluconeogenesis; 0006107: oxaloacetate metabolic process; 0051384: response to glucocorticoid stimulus |
| 36614_at | HSPA5 | 0006916: anti-apoptosis; 0006983: ER overload response; 0006987: activation of signaling protein activity involved in unfolded protein response; 0021589: cerebellum structural organization; 0021680: cerebellar Purkinje cell layer development; 0030433: ER-associated protein catabolic process; 0030512: negative regulation of transforming growth factor beta receptor signaling pathway; 0030968: endoplasmic reticulum unfolded protein response; 0031398: positive regulation of protein ubiquitination; 0042149: cellular response to glucose starvation; 0043066: negative regulation of apoptosis; 0043154: negative regulation of caspase activity; 0051603: proteolysis involved in cellular protein catabolic process; 0060904: regulation of protein folding in endoplasmic reticulum |
| 34853_at | FLRT2 | 0007155: cell adhesion |
| 36937_s_at | PDLIM1 | 0001666: response to hypoxia; 0006979: response to oxidative stress; 0045449: regulation of transcription |
| 32488_at | COL3A1 | 0001501: skeletal system development; 0001568: blood vessel development; 0007160: cell-matrix adhesion; 0007179: transforming growth factor beta receptor signaling pathway; 0007229: integrin-mediated signaling pathway; 0007507: heart development; 0009314: response to radiation; 0018149: peptide cross-linking; 0030168: platelet activation; 0030199: collagen fibril organization; 0032964: collagen biosynthetic process; 0034097: response to cytokine stimulus; 0042060: wound healing; 0043206: fibril organization; 0043588: skin development; 0048565: digestive tract development; 0050777: negative regulation of immune response |
| 36199_at | DAP | 0006915: apoptosis; 0006919: activation of caspase activity; 0008624: induction of apoptosis by extracellular signals; 0010507: negative regulation of autophagy; 0032088: negative regulation of NF-kappaB transcription factor activity; 0034198: cellular response to amino acid starvation |
| 38474_at | CBS | 0001958: endochondral ossification; 0006535: cysteine biosynthetic process from serine; 0006563: L-serine metabolic process; 0006565: L-serine catabolic process; 0008652: cellular amino acid biosynthetic process; 0019343: cysteine biosynthetic process via cystathionine; 0019344: cysteine biosynthetic process; 0019346: transsulfuration; 0019448: L-cysteine catabolic process; 0031667: response to nutrient levels; 0043418: homocysteine catabolic process; 0043506: regulation of JUN kinase activity; 0050667: homocysteine metabolic process; 0051593: response to folic acid; 0070814: hydrogen sulfide biosynthetic process |
| 36671_at | ASNS | 0001889: liver development; 0006520: cellular amino acid metabolic process; 0006529: asparagine biosynthetic process; 0006541: glutamine metabolic process; 0008152: metabolic process; 0008652: cellular amino acid biosynthetic process; 0009416: response to light stimulus; 0009612: response to mechanical stimulus; 0009636: response to toxin; 0031427: response to methotrexate; 0031667: response to nutrient levels; 0032354: response to follicle-stimulating hormone stimulus; 0032870: cellular response to hormone stimulus; 0042149: cellular response to glucose starvation; 0043066: negative regulation of apoptosis; 0043200: response to amino acid stimulus; 0045931: positive regulation of mitotic cell cycle |
| 437_at | MMP3 | 0006508: proteolysis; 0006508: proteolysis; 0008152: metabolic process; 0030574: collagen catabolic process; 0034097: response to cytokine stimulus; 0043200: response to amino acid stimulus |
| 35246_at | TYRO3 | 0006468: protein phosphorylation; 0007155: cell adhesion; 0007165: signal transduction |
| 1828_s_at | FGF2 | 0000186: activation of MAPKK activity; 0000187: activation of MAPK activity; 0000189: nuclear translocation of MAPK; 0001525: angiogenesis; 0001658: branching involved in ureteric bud morphogenesis; 0001759: organ induction; 0001934: positive regulation of protein phosphorylation; 0002042: cell migration involved in sprouting angiogenesis; 0006355: regulation of transcription, DNA-dependent; 0006700: C21-steroid hormone biosynthetic process; 0006915: apoptosis; 0006935: chemotaxis; 0007165: signal transduction; 0007243: intracellular protein kinase cascade; 0007265: Ras protein signal transduction; 0007268: synaptic transmission; 0007275: multicellular organismal development; 0007399: nervous system development; 0008284: positive regulation of cell proliferation; 0008285: negative regulation of cell proliferation; 0008543: fibroblast growth factor receptor signaling pathway; 0009790: embryo development; 0009887: organ morphogenesis; 0010001: glial cell differentiation; 0010552: positive regulation of gene-specific transcription from RNA polymerase II promoter; 0010628: positive regulation of gene expression; 0010863: positive regulation of phospholipase C activity; 0017158: regulation of calcium ion-dependent exocytosis; 0021940: positive regulation of granule cell precursor proliferation; 0030154: cell differentiation; 0030308: negative regulation of cell growth; 0030324: lung development; 0042060: wound healing; 0042660: positive regulation of cell fate specification; 0043536: positive regulation of blood vessel endothelial cell migration; 0043537: negative regulation of blood vessel endothelial cell migration; 0045597: positive regulation of cell differentiation; 0045765: regulation of angiogenesis; 0045766: positive regulation of angiogenesis; 0045892: negative regulation of transcription, DNA-dependent; 0045941: positive regulation of transcription; 0045944: positive regulation of transcription from RNA polymerase II promoter; 0046668: regulation of retinal cell programmed cell death; 0050679: positive regulation of epithelial cell proliferation; 0051726: regulation of cell cycle; 0051781: positive regulation of cell division; 0060045: positive regulation of cardiac muscle cell proliferation; 0060128: adrenocorticotropin hormone secreting cell differentiation; 0060129: thyroid-stimulating hormone-secreting cell differentiation; 0060548: negative regulation of cell death; 0060591: chondroblast differentiation; 0060644: mammary gland epithelial cell differentiation; 0070374: positive regulation of ERK1 and ERK2 cascade |
| 32221_at | MRPS18B | 0006412: translation |
| 38042_at | PRKCA | 0000188: inactivation of MAPK activity; 0000302: response to reactive oxygen species; 0001816: cytokine production; 0001933: negative regulation of protein phosphorylation; 0001934: positive regulation of protein phosphorylation; 0002026: regulation of the force of heart contraction; 0002062: chondrocyte differentiation; 0005975: carbohydrate metabolic process; 0006006: glucose metabolic process; 0006098: pentose-phosphate shunt; 0006468: protein phosphorylation; 0006469: negative regulation of protein kinase activity; 0006629: lipid metabolic process; 0006695: cholesterol biosynthetic process; 0006740: NADPH regeneration; 0006749: glutathione metabolic process; 0006874: cellular calcium ion homeostasis; 0006937: regulation of muscle contraction; 0007568: aging; 0007611: learning or memory; 0008152: metabolic process; 0008285: negative regulation of cell proliferation; 0008624: induction of apoptosis by extracellular signals; 0008629: induction of apoptosis by intracellular signals; 0009051: pentose-phosphate shunt, oxidative branch; 0009612: response to mechanical stimulus; 0009636: response to toxin; 0010734: negative regulation of protein glutathionylation; 0014070: response to organic cyclic substance; 0017148: negative regulation of translation; 0018105: peptidyl-serine phosphorylation; 0018107: peptidyl-threonine phosphorylation; 0019322: pentose biosynthetic process; 0021955: central nervous system neuron axonogenesis; 0023034: intracellular signaling pathway; 0030593: neutrophil chemotaxis; 0032355: response to estradiol stimulus; 0034599: cellular response to oxidative stress; 0035408: histone H3-T6 phosphorylation; 0043249: erythrocyte maturation; 0043434: response to peptide hormone stimulus; 0045471: response to ethanol; 0045822: negative regulation of heart contraction; 0045921: positive regulation of exocytosis; 0046325: negative regulation of glucose import; 0046390: ribose phosphate biosynthetic process; 0046627: negative regulation of insulin receptor signaling pathway; 0046677: response to antibiotic; 0048259: regulation of receptor-mediated endocytosis; 0048661: positive regulation of smooth muscle cell proliferation; 0050729: positive regulation of inflammatory response; 0050730: regulation of peptidyl-tyrosine phosphorylation; 0050930: induction of positive chemotaxis; 0051156: glucose 6-phosphate metabolic process; 0051412: response to corticosterone stimulus; 0051965: positive regulation of synaptogenesis; 0055114: oxidation reduction; 0070555: response to interleukin-1 |
| 222_at | EXT1 | 0001501: skeletal system development; 0001503: ossification; 0006024: glycosaminoglycan biosynthetic process; 0006024: glycosaminoglycan biosynthetic process; 0007165: signal transduction; 0007369: gastrulation; 0007411: axon guidance; 0007420: brain development; 0007492: endoderm development; 0007498: mesoderm development; 0015012: heparan sulfate proteoglycan biosynthetic process; 0015014: heparan sulfate proteoglycan biosynthetic process, polysaccharide chain biosynthetic process; 0021772: olfactory bulb development; 0033692: cellular polysaccharide biosynthetic process |
| 39382_at | TRIM2 | 0005515: protein binding; 0008270: zinc ion binding; 0046872: metal ion binding |
| 33325_at | RPS6KA2 | 0000089: mitotic metaphase; 0001556: oocyte maturation; 0006468: protein phosphorylation; 0007165: signal transduction; 0007243: intracellular protein kinase cascade; 0018105: peptidyl-serine phosphorylation; 0023034: intracellular signaling pathway |
| 40613_at | C6orf62 | 0005622: intracellular |
| 37325_at | FDPS | 0006694: steroid biosynthetic process; 0006695: cholesterol biosynthetic process; 0008299: isoprenoid biosynthetic process; 0008610: lipid biosynthetic process; 0016126: sterol biosynthetic process; 0044419: interspecies interaction between organisms |
| 39782_at | C1D | 0006350: transcription; 0006364: rRNA processing; 0006915: apoptosis; 0016481: negative regulation of transcription; 0045449: regulation of transcription |
| 719_g_at | HTRA1 | 0001558: regulation of cell growth; 0006508: proteolysis; 0030512: negative regulation of transforming growth factor beta receptor signaling pathway; 0030514: negative regulation of BMP signaling pathway |
| 39086_g_at | SSBP1 | 0006260: DNA replication; 0051096: positive regulation of helicase activity; 0070584: mitochondrion morphogenesis |
| 2036_s_at | CD44 | 0001558: regulation of cell growth; 0001658: branching involved in ureteric bud morphogenesis; 0001955: blood vessel maturation; 0002246: wound healing involved in inflammatory response; 0006954: inflammatory response; 0007155: cell adhesion; 0007160: cell-matrix adhesion; 0010628: positive regulation of gene expression; 0014070: response to organic cyclic substance; 0016337: cell-cell adhesion; 0016477: cell migration; 0031175: neuron projection development; 0033031: positive regulation of neutrophil apoptosis; 0033138: positive regulation of peptidyl-serine phosphorylation; 0033189: response to vitamin A; 0034238: macrophage fusion; 0043066: negative regulation of apoptosis; 0043518: negative regulation of DNA damage response, signal transduction by p53 class mediator; 0050731: positive regulation of peptidyl-tyrosine phosphorylation; 0060442: branching involved in prostate gland morphogenesis; 0070374: positive regulation of ERK1 and ERK2 cascade |
| 37766_s_at | PSMC5 | 0006366: transcription from RNA polymerase II promoter; 0016481: negative regulation of transcription; 0030163: protein catabolic process; 0031145: anaphase-promoting complex-dependent proteasomal ubiquitin-dependent protein catabolic process; 0043069: negative regulation of programmed cell death; 0043161: proteasomal ubiquitin-dependent protein catabolic process; 0043193: positive regulation of gene-specific transcription; 0051436: negative regulation of ubiquitin-protein ligase activity involved in mitotic cell cycle; 0051437: positive regulation of ubiquitin-protein ligase activity involved in mitotic cell cycle |
| 32164_at | EXT1 | 0001501: skeletal system development; 0001503: ossification; 0006024: glycosaminoglycan biosynthetic process; 0007165: signal transduction; 0007369: gastrulation; 0007411: axon guidance; 0007420: brain development; 0007492: endoderm development; 0007498: mesoderm development 0015012: heparan sulfate proteoglycan biosynthetic process; 0015014: heparan sulfate proteoglycan biosynthetic process, polysaccharide chain biosynthetic process; 0021772: olfactory bulb development; 0033692: cellular polysaccharide biosynthetic process |
| 40189_at | SET | 0006260: DNA replication; 0006334: nucleosome assembly; 0006337: nucleosome disassembly; 0006913: nucleocytoplasmic transport; 0035067: negative regulation of histone acetylation |
| 36009_at | GPX7 | 0006979: response to oxidative stress; 0055114: oxidation reduction |
| 38066_at | NQO1 | 0006805: xenobiotic metabolic process; 0006809: nitric oxide biosynthetic process; 0006979: response to oxidative stress; 0007271: synaptic transmission, cholinergic; 0009636: response to toxin; 0043086: negative regulation of catalytic activity; 0043525: positive regulation of neuron apoptosis; 0055114: oxidation reduction |
| 297_g_at | TUBB2A | 0007017: microtubule-based process; 0007018: microtubule-based movement; 0007067: mitosis; 0030182: neuron differentiation; 0051258: protein polymerization |
| 32171_at | EIF5 | 0006412: translation; 0006413: translational initiation; 0006446: regulation of translational initiation; 0016070: RNA metabolic process |
| 36537_at | ARHGEF18 | 0006915: apoptosis; 0007264: small GTPase mediated signal transduction; 0008360: regulation of cell shape; 0008624: induction of apoptosis by extracellular signals; 0030036: actin cytoskeleton organization; 0035023: regulation of Rho protein signal transduction |
| 32546_at | FH | 0006099: tricarboxylic acid cycle; 0006106: fumarate metabolic process; 0006108: malate metabolic process; 0048873: homeostasis of number of cells within a tissue |
| 39759_at | QKI | 0001570: vasculogenesis; 0006397: mRNA processing; 0006417: regulation of translation; 0006810: transport; 0007275: multicellular organismal development; 0008366: axon ensheathment; 0008380: RNA splicing; 0030154: cell differentiation; 0042552: myelination; 0042692: muscle cell differentiation; 0042759: long-chain fatty acid biosynthetic process; 0051028: mRNA transport |
| 1521_at | NME1 | 0006183: GTP biosynthetic process; 0006228: UTP biosynthetic process; 0006241: CTP biosynthetic process; 0006350: transcription; 0006355: regulation of transcription, DNA-dependent; 0006897: endocytosis; 0007155: cell adhesion; 0007399: nervous system development; 0008285: negative regulation of cell proliferation; 0009117: nucleotide metabolic process; 0009142: nucleoside triphosphate biosynthetic process; 0030154: cell differentiation; 0042981: regulation of apoptosis; 0043066: negative regulation of apoptosis; 0043388: positive regulation of DNA binding; 0045449: regulation of transcription; 0045618: positive regulation of keratinocyte differentiation; 0045682: regulation of epidermis development; 0050679: positive regulation of epithelial cell proliferation |
| 38797_at | SLC39A14 | 0006810: transport; 0006811: ion transport; 0006826: iron ion transport; 0006829: zinc ion transport; 0030001: metal ion transport; 0055085: transmembrane transport |
| 38815_at | ARPC1A | 0030036: actin cytoskeleton organization; 0030833: regulation of actin filament polymerization |
| 36472_at | NMI | 0006366: transcription from RNA polymerase II promoter; 0006954: inflammatory response; 0007259: JAK-STAT cascade |
| 37459_at | COL8A1 | 0001525: angiogenesis; 0007155: cell adhesion; 0007525: somatic muscle development; 0010811: positive regulation of cell-substrate adhesion; 0048593: camera-type eye morphogenesis; 0050673: epithelial cell proliferation |
| 41133_at | G3BP1 | 0006810: transport; 0007265: Ras protein signal transduction |
| 33899_at | ALDH9A1 | 0001822: kidney development; 0001889: liver development; 0006081: cellular aldehyde metabolic process; 0008152: metabolic process; 0009437: carnitine metabolic process; 0042136: neurotransmitter biosynthetic process; 0042445: hormone metabolic process; 0055114: oxidation reduction; 0055114: oxidation reduction |
| 34198_at | PTPN13 | 0006470: protein dephosphorylation; 0016311: dephosphorylation |
| 39363_at | CHMP2A | 0006810: transport; 0015031: protein transport |
| 1388_g_at | VDR | 0001501: skeletal system development; 0006350: transcription; 0006355: regulation of transcription, DNA-dependent; 0006816: calcium ion transport; 0006874: cellular calcium ion homeostasis; 0007165: signal transduction; 0007275: multicellular organismal development; 0007595: lactation; 0008285: negative regulation of cell proliferation; 0008628: induction of apoptosis by hormones; 0009887: organ morphogenesis; 0010553: negative regulation of gene-specific transcription from RNA polymerase II promoter; 0010980: positive regulation of vitamin D 24-hydroxylase activity; 0016481: negative regulation of transcription; 0045449: regulation of transcription; 0046697: decidualization; 0050892: intestinal absorption; 0060058: positive regulation of apoptosis involved in mammary gland involution; 0060558: regulation of calcidiol 1-monooxygenase activity; 0060745: mammary gland branching involved in pregnancy; 0070561: vitamin D receptor signaling pathway |
| 40099_at | ARHGEF2 | 0006350: transcription; 0006357: regulation of transcription from RNA polymerase II promoter; 0014898: cardiac muscle hypertrophy in response to stress; 0045449: regulation of transcription; 0045944: positive regulation of transcription from RNA polymerase II promoter |
| 37671_at | LAMA4 | 0000132: establishment of mitotic spindle orientation; 0000902: cell morphogenesis; 0006886: intracellular protein transport; 0006915: apoptosis; 0007015: actin filament organization; 0007026: negative regulation of microtubule depolymerization; 0007049: cell cycle; 0007067: mitosis; 0008624: induction of apoptosis by extracellular signals; 0035023: regulation of Rho protein signal transduction; 0042127: regulation of cell proliferation; 0050768: negative regulation of neurogenesis; 0051092: positive regulation of NF-kappaB transcription factor activity; 0051301: cell division |
| 36203_at | ODC1 | 0001568: blood vessel development; 0007155: cell adhesion; 0030155: regulation of cell adhesion; 0030334: regulation of cell migration; 0045995: regulation of embryonic development; 0050873: brown fat cell differentiation |
| 32363_at | CH25H | 0006629: lipid metabolic process; 0006633: fatty acid biosynthetic process; 0006694: steroid biosynthetic process; 0008203: cholesterol metabolic process; 0008610: lipid biosynthetic process; 0016126: sterol biosynthetic process; 0055114: oxidation reduction |
| 40541_at | ASS1 | 0000050: urea cycle; 0000053: argininosuccinate metabolic process; 0001822: kidney development; 0001889: liver development; 0006526: arginine biosynthetic process; 0006953: acute-phase response; 0007584: response to nutrient; 0008652: cellular amino acid biosynthetic process; 0009607: response to biotic stimulus; 0010043: response to zinc ion; 0032496: response to lipopolysaccharide; 0042493: response to drug; 0043434: response to peptide hormone stimulus; 0048545: response to steroid hormone stimulus; 0051384: response to glucocorticoid stimulus |
| 36308_at | ZIC1 | 0007275: multicellular organismal development; 0007389: pattern specification process; 0007389: pattern specification process; 0007399: nervous system development; 0007417: central nervous system development; 0007420: brain development; 0007610: behavior; 0008589: regulation of smoothened signaling pathway; 0030154: cell differentiation; 0042472: inner ear morphogenesis |
| 34376_at | PKIG | 0000122: negative regulation of transcription from RNA polymerase II promoter; 0006469: negative regulation of protein kinase activity; 0007165: signal transduction; 0042308: negative regulation of protein import into nucleus |
| 32669_at | SOCS5 | 0007173: epidermal growth factor receptor signaling pathway; 0009968: negative regulation of signal transduction; 0009968: negative regulation of signal transduction; 0016049: cell growth; 0019221: cytokine-mediated signaling pathway; 0023034: intracellular signaling pathway; 0040008: regulation of growth; 0045627: positive regulation of T-helper 1 cell differentiation; 0045629: negative regulation of T-helper 2 cell differentiation |
| 32165_at | SRSF7 | 0006397: mRNA processing; 0008380: RNA splicing |
| 39532_at | RIN1 | 0006897: endocytosis; 0007165: signal transduction; 0007165: signal transduction |
| 32542_at | FHL1 | 0007275: multicellular organismal development; 0007517: muscle organ development; 0009887: organ morphogenesis; 0016049: cell growth; 0030154: cell differentiation |
| 35324_at | --- |  |
| 1488_at | PTPRK | 0006470: protein dephosphorylation; 0007155: cell adhesion; 0007165: signal transduction; 0007179: transforming growth factor beta receptor signaling pathway; 0008285: negative regulation of cell proliferation; 0010839: negative regulation of keratinocyte proliferation; 0016311: dephosphorylation; 0016477: cell migration; 0016481: negative regulation of transcription; 0030336: negative regulation of cell migration; 0031175: neuron projection development; 0034394: protein localization at cell surface; 0034614: cellular response to reactive oxygen species; 0034644: cellular response to UV; 0045786: negative regulation of cell cycle; 0048041: focal adhesion assembly |
| 41439_at | MYO1B | 0016459: myosin complex |
| 36976_at | CDH11 | 0001501: skeletal system development; 0001503: ossification; 0007155: cell adhesion; 0007156: homophilic cell adhesion; 0016337: cell-cell adhesion |
| 41256_at | EEF1D | 0006412: translation; 0006414: translational elongation; 0043123: positive regulation of I-kappaB kinase/NF-kappaB cascade |
| 33424_at | RPN1 | 0006464: protein modification process; 0006486: protein glycosylation; 0018279: protein N-linked glycosylation via asparagine |
| 36572_r_at | ARL6IP1 | 0006613: cotranslational protein targeting to membrane |
| 39710_at | C5orf13 | 0017015: regulation of transforming growth factor beta receptor signaling pathway; 0031103: axon regeneration; 0045664: regulation of neuron differentiation |
| 34752_at | NEK7 | 0006468: protein phosphorylation |
| 36178_at | SHMT2 | 0006544: glycine metabolic process; 0006545: glycine biosynthetic process; 0006563: L-serine metabolic process; 0006564: L-serine biosynthetic process; 0006730: one-carbon metabolic process; 0008284: positive regulation of cell proliferation; 0019264: glycine biosynthetic process from serine; 0051289: protein homotetramerization |
| 32102_at | SACS | 0006457: protein folding; 0090084: negative regulation of inclusion body assembly |
| 32695_at | HTATSF1 | 0006350: transcription; 0006357: regulation of transcription from RNA polymerase II promoter; 0019079: viral genome replication; 0045449: regulation of transcription |
| 283_at | UQCRC1 | 0006119: oxidative phosphorylation; 0006122: mitochondrial electron transport, ubiquinol to cytochrome c; 0006508: proteolysis; 0006810: transport; 0009060: aerobic respiration; 0014823: response to activity; 0022900: electron transport chain; 0043279: response to alkaloid; 0055114: oxidation reduction |
| 33294_at | EXOSC7 | 0006364: rRNA processing; 0006396: RNA processing; 0006401: RNA catabolic process |
| 943_at | RUNX1 | 0001501: skeletal system development; 0001701: in utero embryonic development; 0001889: liver development; 0006350: transcription; 0006355: regulation of transcription, DNA-dependent; 0007417: central nervous system development; 0030097: hemopoiesis; 0030099: myeloid cell differentiation; 0030182: neuron differentiation; 0030853: negative regulation of granulocyte differentiation; 0030854: positive regulation of granulocyte differentiation; 0032526: response to retinoic acid; 0035162: embryonic hemopoiesis; 0045449: regulation of transcription; 0045766: positive regulation of angiogenesis; 0045944: positive regulation of transcription from RNA polymerase II promoter; 0048266: behavioral response to pain; 0048666: neuron development; 0060216: definitive hemopoiesis |
| 39031_at | COX7A1 | 0006091: generation of precursor metabolites and energy |
| 1452_at | LMO4 | 0001843: neural tube closure; 0006350: transcription; 0006366: transcription from RNA polymerase II promoter; 0045449: regulation of transcription |
| 38102_at | EFHA1 | 0005739: mitochondrion |
| 1778_g_at | RIN1 | 0006897: endocytosis; 0007165: signal transduction |
| 38483_at | CTDNEP1 | 0006470: protein dephosphorylation; 0006998: nuclear envelope organization |
| 32076_at | RCAN2 | 0007417: central nervous system development; 0019722: calcium-mediated signaling |
| 33368_at | AZIN1 | 0006596: polyamine biosynthetic process |
| 40827_at | IARS | 0006412: translation; 0006418: tRNA aminoacylation for protein translation; 0006428: isoleucyl-tRNA aminoacylation |
| 39219_at | CEBPG | 0001889: liver development; 0006350: transcription; 0006355: regulation of transcription, DNA-dependent; 0006357: regulation of transcription from RNA polymerase II promoter; 0006955: immune response; 0016071: mRNA metabolic process; 0030183: B cell differentiation; 0042267: natural killer cell mediated cytotoxicity; 0043353: enucleate erythrocyte differentiation; 0043388: positive regulation of DNA binding; 0043433: negative regulation of transcription factor activity; 0045078: positive regulation of interferon-gamma biosynthetic process; 0045449: regulation of transcription; 0045739: positive regulation of DNA repair; 0051091: positive regulation of transcription factor activity |
| 32849_at | SMC1A | 0000070: mitotic sister chromatid segregation; 0000075: cell cycle checkpoint; 0006281: DNA repair; 0006974: response to DNA damage stimulus; 0007049: cell cycle; 0007052: mitotic spindle organization; 0007062: sister chromatid cohesion; 0007064: mitotic sister chromatid cohesion; 0007067: mitosis; 0007126: meiosis; 0008380: RNA splicing; 0009314: response to radiation; 0032876: negative regulation of DNA endoreduplication; 0042770: signal transduction in response to DNA damage; 0051276: chromosome organization; 0051301: cell division |
| 32781_f_at | DST | 0007050: cell cycle arrest; 0007155: cell adhesion; 0007229: integrin-mediated signaling pathway; 0045104: intermediate filament cytoskeleton organization |
| 32551_at | EFEMP1 | 0007173: epidermal growth factor receptor signaling pathway; 0007601: visual perception; 0018108: peptidyl-tyrosine phosphorylation; 0032331: negative regulation of chondrocyte differentiation; 0045449: regulation of transcription |
| 41421_at | CAMTA2 | 0006350: transcription; 0006357: regulation of transcription from RNA polymerase II promoter; 0014898: cardiac muscle hypertrophy in response to stress; 0045449: regulation of transcription; 0045944: positive regulation of transcription from RNA polymerase II promoter |
| 35396_at | HAS2 | 0005887: integral to plasma membrane; 0016020: membrane; 0016021: integral to membrane |
| 33821_at | ELOVL5 | 0006633: fatty acid biosynthetic process; 0008610: lipid biosynthetic process; 0019432: triglyceride biosynthetic process |
| 831_at | DDX10 | 0000166: nucleotide binding; 0003723: RNA binding; 0003724: RNA helicase activity; 0004386: helicase activity; 0005524: ATP binding; 0008026: ATP-dependent helicase activity; 0016787: hydrolase activity |
| 40024_at | STAC | 0007165: signal transduction; 0023034: intracellular signaling pathway; 0034605: cellular response to heat |
| 718_at | HTRA1 | 0001558: regulation of cell growth; 0006508: proteolysis; 0030512: negative regulation of transforming growth factor beta receptor signaling pathway; 0030514: negative regulation of BMP signaling pathway |
| 31510_s_at | H3F3B | 0006334: nucleosome assembly; 0007420: brain development; 0009725: response to hormone stimulus |
| 37611_at | TNFRSF11B | 0001501: skeletal system development; 0006915: apoptosis; 0007165: signal transduction; 0007584: response to nutrient; 0010035: response to inorganic substance; 0030198: extracellular matrix organization; 0032026: response to magnesium ion; 0042489: negative regulation of odontogenesis of dentine-containing tooth; 0042493: response to drug; 0043627: response to estrogen stimulus; 0045779: negative regulation of bone resorption; 0046685: response to arsenic |
| 40506_s_at | PABPC4 | 0006396: RNA processing; 0006401: RNA catabolic process; 0006412: translation; 0007596: blood coagulation |
| 34297_at | GPR37 | 0007165: signal transduction; 0007186: G-protein coupled receptor protein signaling pathway |
| 39878_at | PCDH9 | 0007155: cell adhesion; 0007156: homophilic cell adhesion; 0030900: forebrain development |
| 36960_at | PHC2 | 0007275: multicellular organismal development |
| 32786_at | JUNB | 0001570: vasculogenesis; 0001649: osteoblast differentiation; 0001701: in utero embryonic development; 0001829: trophectodermal cell differentiation; 0006350: transcription; 0006355: regulation of transcription, DNA-dependent; 0006357: regulation of transcription from RNA polymerase II promoter; 0006366: transcription from RNA polymerase II promoter; 0007565: female pregnancy; 0009416: response to light stimulus; 0009612: response to mechanical stimulus; 0009987: cellular process; 0014070: response to organic cyclic substance; 0030316: osteoclast differentiation; 0032570: response to progesterone stimulus; 0032870: cellular response to hormone stimulus; 0033687: osteoblast proliferation; 0034097: response to cytokine stimulus; 0042493: response to drug; 0043434: response to peptide hormone stimulus; 0045449: regulation of transcription; 0045597: positive regulation of cell differentiation; 0046022: positive regulation of transcription from RNA polymerase II promoter during mitosis; 0046697: decidualization; 0051412: response to corticosterone stimulus; 0051591: response to cAMP; 0051726: regulation of cell cycle; 0060136: embryonic process involved in female pregnancy; 0060716: labyrinthine layer blood vessel development |
| 37147_at | CLEC11A | 0008284: positive regulation of cell proliferation |
| 36098_at | SRSF1 | 0006376: mRNA splice site selection; 0006397: mRNA processing; 0008380: RNA splicing |
| 38277_at | PPP3CB | 0000082: G1/S transition of mitotic cell cycle; 0001915: negative regulation of T cell mediated cytotoxicity; 0001975: response to amphetamine; 0006470: protein dephosphorylation; 0006606: protein import into nucleus; 0006754: ATP biosynthetic process; 0006816: calcium ion transport; 0006950: response to stress; 0007507: heart development; 0010468: regulation of gene expression; 0016311: dephosphorylation; 0030217: T cell differentiation; 0034097: response to cytokine stimulus; 0043029: T cell homeostasis; 0046716: muscle cell homeostasis; 0048741: skeletal muscle fiber development; 0050804: regulation of synaptic transmission; 0060079: regulation of excitatory postsynaptic membrane potential |
| 33666_at | HNRNPC | 0000398: nuclear mRNA splicing, via spliceosome; 0006397: mRNA processing; 0008380: RNA splicing |
| 33203_s_at | FOXD1 | 0000122: negative regulation of transcription from RNA polymerase II promoter; 0001658: branching involved in ureteric bud morphogenesis; 0001755: neural crest cell migration; 0001822: kidney development; 0006350: transcription; 0006355: regulation of transcription, DNA-dependent; 0007389: pattern specification process; 0007422: peripheral nervous system development; 0009790: embryo development; 0010551: regulation of gene-specific transcription from RNA polymerase II promoter; 0010628: positive regulation of gene expression; 0030318: melanocyte differentiation; 0030513: positive regulation of BMP signaling pathway; 0032582: negative regulation of gene-specific transcription; 0045449: regulation of transcription; 0045944: positive regulation of transcription from RNA polymerase II promoter; 0048484: enteric nervous system development; 0048485: sympathetic nervous system development; 0048846: axon extension involved in axon guidance; 0048937: lateral line nerve glial cell development; 0050935: iridophore differentiation; 0051090: regulation of transcription factor activity; 0051216: cartilage development; 0060678: dichotomous subdivision of terminal units involved in ureteric bud branching; 0072213: metanephric capsule development; 0072267: metanephric capsule specification; 0090184: positive regulation of kidney development |
| 723_s_at | HNRNPC | 0000398: nuclear mRNA splicing, via spliceosome; 0006397: mRNA processing; 0008380: RNA splicing |
| 41385_at | EPB41L3 | 0030866: cortical actin cytoskeleton organization |
| 34336_at | KARS | 0006412: translation; 0006418: tRNA aminoacylation for protein translation; 0006430: lysyl-tRNA aminoacylation; 0008033: tRNA processing; 0015966: diadenosine tetraphosphate biosynthetic process; 0044419: interspecies interaction between organisms |
| 2087_s_at | CDH11 | 0001501: skeletal system development; 0001503: ossification; 0007155: cell adhesion; 0007156: homophilic cell adhesion; 0016337: cell-cell adhesion |
| 39342_at | MARS | 0006412: translation; 0006418: tRNA aminoacylation for protein translation; 0006431: methionyl-tRNA aminoacylation |
| 37762_at | EMP1 | 0007275: multicellular organismal development; 0008283: cell proliferation; 0008544: epidermis development; 0016049: cell growth; 0030855: epithelial cell differentiation |
| 37336_at | UBXN4 | 0006986: response to unfolded protein |
| 34396_at | ASXL1 | 0006350: transcription; 0016568: chromatin modification; 0045449: regulation of transcription |
| 38354_at | CEBPB | 0001892: embryonic placenta development; 0006350: transcription; 0006355: regulation of transcription, DNA-dependent; 0006366: transcription from RNA polymerase II promoter; 0006916: anti-apoptosis; 0006917: induction of apoptosis; 0006953: acute-phase response; 0006954: inflammatory response; 0006955: immune response; 0030154: cell differentiation; 0030182: neuron differentiation; 0033598: mammary gland epithelial cell proliferation; 0045408: regulation of interleukin-6 biosynthetic process; 0045444: fat cell differentiation; 0045449: regulation of transcription; 0045941: positive regulation of transcription; 0045944: positive regulation of transcription from RNA polymerase II promoter; 0060644: mammary gland epithelial cell differentiation |
| 39945_at | FAP | 0006508: proteolysis; 0010716: negative regulation of extracellular matrix disassembly; 0043542: endothelial cell migration |
| 1314_at | PSMD1 | 0031145: anaphase-promoting complex-dependent proteasomal ubiquitin-dependent protein catabolic process; 0042176: regulation of protein catabolic process; 0051436: negative regulation of ubiquitin-protein ligase activity involved in mitotic cell cycle; 0051437: positive regulation of ubiquitin-protein ligase activity involved in mitotic cell cycle |
| 38804_at | CSE1L | 0006810: transport; 0006886: intracellular protein transport; 0006915: apoptosis; 0008283: cell proliferation; 0015031: protein transport |
| 31863_at | RRP1B | 0006364: rRNA processing |
| 40364_at | SLC31A1 | 0006810: transport; 0006811: ion transport; 0006825: copper ion transport; 0006825: copper ion transport |
| 377_g_at | SEMA3C | 0001755: neural crest cell migration; 0001756: somitogenesis; 0001974: blood vessel remodeling; 0003151: outflow tract morphogenesis; 0003215: cardiac right ventricle morphogenesis; 0003350: pulmonary myocardium development; 0006955: immune response; 0007275: multicellular organismal development; 0007507: heart development; 0009791: post-embryonic development; 0021915: neural tube development; 0042493: response to drug; 0060174: limb bud formation; 0060666: dichotomous subdivision of terminal units involved in salivary gland branching |
| 34850_at | UBE2E3 | 0040008: regulation of growth; 0043687: post-translational protein modification; 0051246: regulation of protein metabolic process; 0070534: protein K63-linked ubiquitination; 0070936: protein K48-linked ubiquitination; 0070979: protein K11-linked ubiquitination |
| 35836_at | NUDC | 0007049: cell cycle; 0007067: mitosis; 0007097: nuclear migration; 0007275: multicellular organismal development; 0008283: cell proliferation; 0043434: response to peptide hormone stimulus; 0051301: cell division |
| 31801_at | TMF1 | 0006350: transcription; 0006355: regulation of transcription, DNA-dependent; 0006366: transcription from RNA polymerase II promoter; 0045449: regulation of transcription |
| 36131_at | CLIC1 | 0006810: transport; 0006811: ion transport; 0006821: chloride transport; 0006821: chloride transport; 0007165: signal transduction |
| 851_s_at | IRS1 | 0002053: positive regulation of mesenchymal cell proliferation; 0007165: signal transduction; 0008284: positive regulation of cell proliferation; 0008286: insulin receptor signaling pathway; 0009749: response to glucose stimulus; 0010907: positive regulation of glucose metabolic process; 0014065: phosphoinositide 3-kinase cascade; 0030335: positive regulation of cell migration; 0030879: mammary gland development; 0032000: positive regulation of fatty acid beta-oxidation; 0032868: response to insulin stimulus; 0032869: cellular response to insulin stimulus; 0042327: positive regulation of phosphorylation; 0042593: glucose homeostasis; 0043434: response to peptide hormone stimulus; 0043491: protein kinase B signaling cascade; 0043552: positive regulation of phosphoinositide 3-kinase activity; 0045725: positive regulation of glycogen biosynthetic process; 0046326: positive regulation of glucose import; 0046627: negative regulation of insulin receptor signaling pathway; 0046628: positive regulation of insulin receptor signaling pathway; 0046676: negative regulation of insulin secretion; 0048009: insulin-like growth factor receptor signaling pathway; 0051291: protein heterooligomerization; 0070094: positive regulation of glucagon secretion; 0090275: negative regulation of somatostatin secretion |
| 35729_at | MYO1D | 0005790: smooth endoplasmic reticulum; 0016459: myosin complex; 0030673: axolemma |
| 37406_at | MAPRE2 | 0007049: cell cycle; 0007067: mitosis; 0007165: signal transduction; 0008283: cell proliferation; 0051301: cell division |
| 36926_at | MAPK6 | 0006468: protein phosphorylation; 0007049: cell cycle; 0007165: signal transduction |
| 2054_g_at | CDH2 | 0007155: cell adhesion; 0007156: homophilic cell adhesion; 0007157: heterophilic cell-cell adhesion; 0007416: synapse assembly; 0016337: cell-cell adhesion; 0016339: calcium-dependent cell-cell adhesion; 0016477: cell migration; 0031641: regulation of myelination; 0032880: regulation of protein localization; 0035023: regulation of Rho protein signal transduction; 0048514: blood vessel morphogenesis; 0050770: regulation of axonogenesis; 0051291: protein heterooligomerization |
| 37736_at | PCMT1 | 0006464: protein modification process; 0006479: protein methylation; 0030091: protein repair; 0046498: S-adenosylhomocysteine metabolic process; 0046500: S-adenosylmethionine metabolic process |
| 37755_at | BTBD3 | 0005515: protein binding |
| 38753_at | XPOT | 0006409: tRNA export from nucleus; 0006810: transport; 0006886: intracellular protein transport |
| 41049_at | IRS1 | 0002053: positive regulation of mesenchymal cell proliferation; 0007165: signal transduction; 0008284: positive regulation of cell proliferation; 0008286: insulin receptor signaling pathway; 0009749: response to glucose stimulus; 0010907: positive regulation of glucose metabolic process; 0014065: phosphoinositide 3-kinase cascade; 0030335: positive regulation of cell migration; 0030879: mammary gland development; 0032000: positive regulation of fatty acid beta-oxidation; 0032868: response to insulin stimulus; 0032869: cellular response to insulin stimulus; 0042327: positive regulation of phosphorylation; 0042593: glucose homeostasis; 0043434: response to peptide hormone stimulus; 0043491: protein kinase B signaling cascade; 0043552: positive regulation of phosphoinositide 3-kinase activity; 0045725: positive regulation of glycogen biosynthetic process; 0046326: positive regulation of glucose import; 0046627: negative regulation of insulin receptor signaling pathway; 0046628: positive regulation of insulin receptor signaling pathway; 0046676: negative regulation of insulin secretion; 0048009: insulin-like growth factor receptor signaling pathway; 0051291: protein heterooligomerization; 0070094: positive regulation of glucagon secretion; 0090275: negative regulation of somatostatin secretion |
| 38818_at | SPTLC1 | 0006665: sphingolipid metabolic process; 0006686: sphingomyelin biosynthetic process; 0009058: biosynthetic process; 0030148: sphingolipid biosynthetic process; 0046511: sphinganine biosynthetic process; 0046512: sphingosine biosynthetic process; 0046513: ceramide biosynthetic process |
| 36600_at | PSME1 | 0019884: antigen processing and presentation of exogenous antigen; 0031145: anaphase-promoting complex-dependent proteasomal ubiquitin-dependent protein catabolic process; 0051436: negative regulation of ubiquitin-protein ligase activity involved in mitotic cell cycle; 0051437: positive regulation of ubiquitin-protein ligase activity involved in mitotic cell cycle |
| 41776_at | ATOX1 | 0006810: transport; 0006811: ion transport; 0006825: copper ion transport; 0006878: cellular copper ion homeostasis; 0006878: cellular copper ion homeostasis; 0006979: response to oxidative stress; 0030001: metal ion transport |
| 688_at | PSMC1 | 0030163: protein catabolic process; 0031145: anaphase-promoting complex-dependent proteasomal ubiquitin-dependent protein catabolic process; 0051436: negative regulation of ubiquitin-protein ligase activity involved in mitotic cell cycle; 0051437: positive regulation of ubiquitin-protein ligase activity involved in mitotic cell cycle |
| 40556_at | RCN1 | 0005783: endoplasmic reticulum; 0005788: endoplasmic reticulum lumen |
| 36610_at | R3HDM1 | 0003676: nucleic acid binding |
| 32112_s_at | AIM1 | 0005529: sugar binding |
| 34773_at | TBCA | 0006457: protein folding; 0007021: tubulin complex assembly; 0007023: post-chaperonin tubulin folding pathway |
| 34823_at | DPP4 | 0001666: response to hypoxia; 0002709: regulation of T cell mediated immunity; 0006508: proteolysis; 0007155: cell adhesion; 0008284: positive regulation of cell proliferation; 0010716: negative regulation of extracellular matrix disassembly; 0031295: T cell costimulation; 0033632: regulation of cell-cell adhesion mediated by integrin; 0042110: T cell activation; 0043542: endothelial cell migration; 0051234: establishment of localization |
| 37966_at | PARVB | 0007155: cell adhesion |
| 39028_at | IPO5 | 0006607: NLS-bearing substrate import into nucleus; 0006810: transport; 0006886: intracellular protein transport; 0015031: protein transport; 0044419: interspecies interaction between organisms |
| 41488_at | LYRM1 | 0005634: nucleus; 0005739: mitochondrion |
| 33881_at | ACSL3 | 0006629: lipid metabolic process; 0006631: fatty acid metabolic process; 0006633: fatty acid biosynthetic process; 0007420: brain development; 0007584: response to nutrient; 0008152: metabolic process; 0014070: response to organic cyclic substance; 0019432: triglyceride biosynthetic process |
| 35239_at | EMD | 0006936: muscle contraction; 0007517: muscle organ development; 0031468: nuclear envelope reassembly; 0035414: negative regulation of catenin protein nuclear translocation; 0046827: positive regulation of protein export from nucleus; 0048147: negative regulation of fibroblast proliferation; 0060828: regulation of canonical Wnt receptor signaling pathway; 0071363: cellular response to growth factor stimulus |
| 41656_at | NMT2 | 0006499: N-terminal protein myristoylation; 0009249: protein lipoylation |
| 37445_at | TMEM5 | 0005887: integral to plasma membrane; 0016020: membrane; 0016021: integral to membrane |
| 34349_at | SEC63 | 0006457: protein folding; 0006612: protein targeting to membrane; 0006810: transport; 0015031: protein transport |
| 39756_g_at | XBP1 | 0006350: transcription; 0006355: regulation of transcription, DNA-dependent; 0006955: immune response; 0045449: regulation of transcription |
| 39389_at | CD9 | 0006928: cellular component movement; 0007155: cell adhesion; 0007338: single fertilization; 0007342: fusion of sperm to egg plasma membrane; 0008285: negative regulation of cell proliferation; 0030168: platelet activation; 0030913: paranodal junction assembly |
| 37668_at | C1QBP | 0006955: immune response; 0044419: interspecies interaction between organisms |
| 31897_at | FILIP1L | 0005634: nucleus; 0005737: cytoplasm; 0016020: membrane; 0016459: myosin complex |
| 39744_at | DDX3X | 0044419: interspecies interaction between organisms |
| 34787_at | NRD1 | 0006508: proteolysis; 0007528: neuromuscular junction development; 0008283: cell proliferation; 0016477: cell migration; 0051044: positive regulation of membrane protein ectodomain proteolysis; 0052548: regulation of endopeptidase activity |
| 36963_at | PGD | 0006098: pentose-phosphate shunt; 0008152: metabolic process; 0009051: pentose-phosphate shunt, oxidative branch; 0019322: pentose biosynthetic process; 0055114: oxidation reduction; 0055114: oxidation reduction |
| 571_at | NAP1L1 | 0006260: DNA replication; 0006334: nucleosome assembly; 0008284: positive regulation of cell proliferation |
| 40832_s_at | TOR1AIP1 | 0005634: nucleus; 0005635: nuclear envelope; 0005637: nuclear inner membrane; 0016020: membrane; 0016021: integral to membrane |
| 39073_at | NME1 | 0006183: GTP biosynthetic process; 0006228: UTP biosynthetic process; 0006241: CTP biosynthetic process; 0006350: transcription; 0006355: regulation of transcription, DNA-dependent; 0006897: endocytosis; 0007155: cell adhesion; 0007399: nervous system development; 0008285: negative regulation of cell proliferation; 0009117: nucleotide metabolic process; 0009142: nucleoside triphosphate biosynthetic process; 0030154: cell differentiation; 0042981: regulation of apoptosis; 0043066: negative regulation of apoptosis; 0043388: positive regulation of DNA binding; 0045449: regulation of transcription; 0045618: positive regulation of keratinocyte differentiation; 0045682: regulation of epidermis development; 0050679: positive regulation of epithelial cell proliferation |
| 1250_at | PRKDC | 0000723: telomere maintenance; 0001756: somitogenesis; 0002326: B cell lineage commitment; 0002328: pro-B cell differentiation; 0002360: T cell lineage commitment; 0006281: DNA repair; 0006302: double-strand break repair; 0006303: double-strand break repair via nonhomologous end joining; 0006310: DNA recombination; 0006464: protein modification process; 0006915: apoptosis; 0006974: response to DNA damage stimulus; 0007420: brain development; 0007507: heart development; 0010332: response to gamma radiation; 0010552: positive regulation of gene-specific transcription from RNA polymerase II promoter; 0018105: peptidyl-serine phosphorylation; 0030098: lymphocyte differentiation; 0031648: protein destabilization; 0032869: cellular response to insulin stimulus; 0033077: T cell differentiation in thymus; 0033152: immunoglobulin V(D)J recombination; 0033153: T cell receptor V(D)J recombination; 0035234: germ cell programmed cell death; 0043065: positive regulation of apoptosis |
| 39800_s_at | HAX1 | 0005625: soluble fraction; 0005634: nucleus; 0005635: nuclear envelope; 0005739: mitochondrion; 0005783: endoplasmic reticulum; 0015629: actin cytoskeleton; 0016020: membrane; 0016023: cytoplasmic membrane-bounded vesicle; 0016529: sarcoplasmic reticulum; 0031410: cytoplasmic vesicle; 0031965: nuclear membrane |
| 32408_s_at | HNRNPC; HNRNPCL1:/ | 0000398: nuclear mRNA splicing, via spliceosome; 0006397: mRNA processing; 0008380: RNA splicing |
| 35298_at | EIF3D | 0001732: formation of translation initiation complex; 0006412: translation; 0006413: translational initiation |
| 36532_at | SYNJ2 | 0007420: brain development; 0016311: dephosphorylation; 0046855: inositol phosphate dephosphorylation; 0046856: phosphoinositide dephosphorylation; 0048312: intracellular distribution of mitochondria |
| 36474_at | KIAA0776 | 0031397: negative regulation of protein ubiquitination; 0032088: negative regulation of NF-kappaB transcription factor activity; 0071569: protein ufmylation |
| 39397_at | NR2F2 | 0000122: negative regulation of transcription from RNA polymerase II promoter; 0001764: neuron migration; 0001937: negative regulation of endothelial cell proliferation; 0001945: lymph vessel development; 0006350: transcription; 0006355: regulation of transcription, DNA-dependent; 0006357: regulation of transcription from RNA polymerase II promoter; 0006629: lipid metabolic process; 0007165: signal transduction; 0007519: skeletal muscle tissue development; 0009952: anterior/posterior pattern formation; 0009956: radial pattern formation; 0010596: negative regulation of endothelial cell migration; 0030900: forebrain development; 0032582: negative regulation of gene-specific transcription; 0043193: positive regulation of gene-specific transcription; 0045449: regulation of transcription; 0045736: negative regulation of cyclin-dependent protein kinase activity; 0045941: positive regulation of transcription; 0048514: blood vessel morphogenesis; 0060173: limb development; 0060849: regulation of transcription involved in lymphatic endothelial cell fate commitment |
| 37334_at | HNRNPA0 | 0006397: mRNA processing; 0008380: RNA splicing |
| 38527_at | NONO | 0006281: DNA repair; 0006310: DNA recombination; 0006350: transcription; 0006397: mRNA processing; 0006974: response to DNA damage stimulus; 0008380: RNA splicing; 0045449: regulation of transcription |
| 41268_g_at | TCF25 | 0000122: negative regulation of transcription from RNA polymerase II promoter; 0006350: transcription; 0007507: heart development; 0045449: regulation of transcription |
| 35785_at | GABARAPL1 | 0004872: receptor activity; 0005515: protein binding; 0048487: beta-tubulin binding; 0050811: GABA receptor binding |
| 33247_at | PSMD14 | 0006511: ubiquitin-dependent protein catabolic process; 0031145: anaphase-promoting complex-dependent proteasomal ubiquitin-dependent protein catabolic process; 0051436: negative regulation of ubiquitin-protein ligase activity involved in mitotic cell cycle; 0051437: positive regulation of ubiquitin-protein ligase activity involved in mitotic cell cycle; 0061136: regulation of proteasomal protein catabolic process; 0070536: protein K63-linked deubiquitination |
| 37142_at | GFRA1 | 0007166: cell surface receptor linked signaling pathway; 0007399: nervous system development |
| 39597_at | ABLIM3 | 0007010: cytoskeleton organization; 0045944: positive regulation of transcription from RNA polymerase II promoter |
| 38977_at | YARS | 0006412: translation; 0006418: tRNA aminoacylation for protein translation; 0006437: tyrosyl-tRNA aminoacylation; 0006437: tyrosyl-tRNA aminoacylation; 0006915: apoptosis |
| 32319_at | TNFSF4 | 0001816: cytokine production; 0006954: inflammatory response; 0006955: immune response; 0007165: signal transduction; 0008203: cholesterol metabolic process; 0008284: positive regulation of cell proliferation; 0032582: negative regulation of gene-specific transcription; 0042098: T cell proliferation; 0043433: negative regulation of transcription factor activity; 0050710: negative regulation of cytokine secretion; 0051024: positive regulation of immunoglobulin secretion |
| 41724_at | BCAP31 | 0006810: transport; 0006886: intracellular protein transport; 0006915: apoptosis; 0006955: immune response; 0007283: spermatogenesis; 0015031: protein transport; 0016192: vesicle-mediated transport |
| 40875_s_at | SNRNP70 | 0000398: nuclear mRNA splicing, via spliceosome; 0006397: mRNA processing; 0008380: RNA splicing; 0043484: regulation of RNA splicing |
| 38012_at | FBN2 | 0009653: anatomical structure morphogenesis; 0030326: embryonic limb morphogenesis; 0030501: positive regulation of bone mineralization; 0035108: limb morphogenesis; 0035583: negative regulation of transforming growth factor beta receptor signaling pathway by extracellular sequestering of TGFbeta; 0045669: positive regulation of osteoblast differentiation; 0060346: bone trabecula formation |
| 41131_f_at | HNRNPH2 | 0008380: RNA splicing |
| 37739_at | SSRP1 | 0006260: DNA replication; 0006281: DNA repair; 0006350: transcription; 0006974: response to DNA damage stimulus; 0045449: regulation of transcription |
| 40842_at | SNRPA | 0000398: nuclear mRNA splicing, via spliceosome; 0006397: mRNA processing; 0008380: RNA splicing |
| 39347_at | AP2S1 | 0006810: transport; 0006886: intracellular protein transport; 0015031: protein transport; 0016192: vesicle-mediated transport; 0030100: regulation of endocytosis; 0048268: clathrin coat assembly; 0050690: regulation of defense response to virus by virus |
| 40910_at | CAPZA1 | 0006461: protein complex assembly; 0006928: cellular component movement; 0030036: actin cytoskeleton organization; 0051693: actin filament capping |
| 36849_at | ARHGAP29 | 0007165: signal transduction; 0007266: Rho protein signal transduction |
| 36550_at | RIN2 | 0006897: endocytosis; 0007165: signal transduction; 0007264: small GTPase mediated signal transduction |
| 36492_at | PSMD9 | 0006511: ubiquitin-dependent protein catabolic process; 0031145: anaphase-promoting complex-dependent proteasomal ubiquitin-dependent protein catabolic process; 0032024: positive regulation of insulin secretion; 0045941: positive regulation of transcription; 0046676: negative regulation of insulin secretion; 0051436: negative regulation of ubiquitin-protein ligase activity involved in mitotic cell cycle; 0051437: positive regulation of ubiquitin-protein ligase activity involved in mitotic cell cycle; 0070682: proteasome regulatory particle assembly |
| 956_at | TUBB2C | 0006928: cellular component movement; 0007017: microtubule-based process; 0007018: microtubule-based movement; 0042267: natural killer cell mediated cytotoxicity; 0051258: protein polymerization |
| 39029_at | BUD31 | 0006357: regulation of transcription from RNA polymerase II promoter |
| 39079_at | ERH | 0006139: nucleobase, nucleoside, nucleotide and nucleic acid metabolic process; 0006213: pyrimidine nucleoside metabolic process; 0007049: cell cycle |
| 39056_at | PAICS | 0006164: purine nucleotide biosynthetic process; 0006189: 'de novo' IMP biosynthetic process; 0009113: purine base biosynthetic process; 0009168: purine ribonucleoside monophosphate biosynthetic process |
| 1629_s_at | PTPN13 | 0006470: protein dephosphorylation; 0016311: dephosphorylation |
| 38364_at | TLE4 | 0000122: negative regulation of transcription from RNA polymerase II promoter; 0006350: transcription; 0016055: Wnt receptor signaling pathway; 0016481: negative regulation of transcription; 0045449: regulation of transcription |
| 41419_at | GULP1 | 0006810: transport; 0006869: lipid transport; 0006909: phagocytosis; 0006911: phagocytosis, engulfment; 0006915: apoptosis |
| 37025_at | LITAF | 0006350: transcription; 0006357: regulation of transcription from RNA polymerase II promoter; 0006915: apoptosis; 0043123: positive regulation of I-kappaB kinase/NF-kappaB cascade; 0045449: regulation of transcription |
| 38007_at | NF2 | 0001707: mesoderm formation; 0001953: negative regulation of cell-matrix adhesion; 0006469: negative regulation of protein kinase activity; 0007398: ectoderm development; 0008156: negative regulation of DNA replication; 0008285: negative regulation of cell proliferation; 0014010: Schwann cell proliferation; 0022408: negative regulation of cell-cell adhesion; 0030036: actin cytoskeleton organization; 0030336: negative regulation of cell migration; 0035330: regulation of hippo signaling cascade; 0042127: regulation of cell proliferation; 0042475: odontogenesis of dentine-containing tooth; 0042518: negative regulation of tyrosine phosphorylation of Stat3 protein; 0042524: negative regulation of tyrosine phosphorylation of Stat5 protein; 0043409: negative regulation of MAPKKK cascade; 0045216: cell-cell junction organization; 0046426: negative regulation of JAK-STAT cascade; 0051496: positive regulation of stress fiber assembly; 0070306: lens fiber cell differentiation |
| 39420_at | DDIT3 | 0001975: response to amphetamine; 0006350: transcription; 0006355: regulation of transcription, DNA-dependent; 0006974: response to DNA damage stimulus; 0006979: response to oxidative stress; 0006983: ER overload response; 0007049: cell cycle; 0007050: cell cycle arrest; 0007568: aging; 0007584: response to nutrient; 0008219: cell death; 0030968: endoplasmic reticulum unfolded protein response; 0032792: negative regulation of CREB transcription factor activity; 0034976: response to endoplasmic reticulum stress; 0042493: response to drug; 0042542: response to hydrogen peroxide; 0042789: mRNA transcription from RNA polymerase II promoter; 0042981: regulation of apoptosis; 0043065: positive regulation of apoptosis; 0043433: negative regulation of transcription factor activity; 0043620: regulation of transcription in response to stress; 0045449: regulation of transcription; 0045454: cell redox homeostasis; 0045941: positive regulation of transcription; 0045944: positive regulation of transcription from RNA polymerase II promoter; 0048568: embryonic organ development; 2000016: negative regulation of determination of dorsal identity |
| 41833_at | JTB | 0005624: membrane fraction; 0005887: integral to plasma membrane; 0016020: membrane; 0016021: integral to membrane |
| 36579_at | UBE4A | 0000209: protein polyubiquitination; 0006511: ubiquitin-dependent protein catabolic process; 0016567: protein ubiquitination |
| 39039_s_at | UBE2J1 | 0043687: post-translational protein modification; 0051246: regulation of protein metabolic process |
| 41161_at | DAXX | 0000281: cytokinesis after mitosis; 0006350: transcription; 0006915: apoptosis; 0007257: activation of JUN kinase activity; 0008625: induction of apoptosis via death domain receptors; 0016481: negative regulation of transcription; 0030521: androgen receptor signaling pathway; 0031396: regulation of protein ubiquitination; 0044419: interspecies interaction between organisms; 0045449: regulation of transcription; 0045892: negative regulation of transcription, DNA-dependent |
| 32789_at | NCBP2 | 0000184: nuclear-transcribed mRNA catabolic process, nonsense-mediated decay; 0000184: nuclear-transcribed mRNA catabolic process, nonsense-mediated decay; 0000387: spliceosomal snRNP assembly; 0006370: mRNA capping; 0006397: mRNA processing; 0006408: snRNA export from nucleus; 0006417: regulation of translation; 0006446: regulation of translational initiation; 0006810: transport; 0008380: RNA splicing; 0031047: gene silencing by RNA; 0046833: positive regulation of RNA export from nucleus; 0051028: mRNA transport |
| 38614_s_at | OGT | 0006493: protein O-linked glycosylation; 0007165: signal transduction; 0007584: response to nutrient; 0070207: protein homotrimerization |
| 34202_at | PVRL3 | 0002089: lens morphogenesis in camera-type eye; 0007155: cell adhesion; 0007156: homophilic cell adhesion; 0009566: fertilization; 0016337: cell-cell adhesion; 0060042: retina morphogenesis in camera-type eye |
| 167_at | EIF5 | 0006412: translation; 0006413: translational initiation; 0006446: regulation of translational initiation; 0016070: RNA metabolic process |
| 32305_at | COL1A2 | 0001501: skeletal system development; 0001568: blood vessel development; 0007179: transforming growth factor beta receptor signaling pathway; 0007266: Rho protein signal transduction; 0008217: regulation of blood pressure; 0030199: collagen fibril organization; 0042476: odontogenesis; 0043589: skin morphogenesis; 0070208: protein heterotrimerization |
| 40856_at | SERPINF1 | 0001822: kidney development; 0007275: multicellular organismal development; 0007568: aging; 0007614: short-term memory; 0008283: cell proliferation; 0016525: negative regulation of angiogenesis; 0032526: response to retinoic acid; 0050728: negative regulation of inflammatory response; 0050769: positive regulation of neurogenesis; 0051384: response to glucocorticoid stimulus; 0060770: negative regulation of epithelial cell proliferation involved in prostate gland development |
| 37842_at | MDFIC | 0006350: transcription; 0007257: activation of JUN kinase activity; 0030111: regulation of Wnt receptor signaling pathway; 0044419: interspecies interaction between organisms; 0045449: regulation of transcription; 0045893: positive regulation of transcription, DNA-dependent; 0050434: positive regulation of viral transcription |
| 36857_at | RAD1 | 0000075: cell cycle checkpoint; 0000077: DNA damage checkpoint; 0006260: DNA replication; 0006281: DNA repair; 0006281: DNA repair; 0006412: translation; 0006974: response to DNA damage stimulus; 0007128: meiotic prophase I; 0042254: ribosome biogenesis |
| 40824_at | XPO7 | 0006611: protein export from nucleus; 0006810: transport; 0006886: intracellular protein transport; 0015031: protein transport; 0051028: mRNA transport; 0055085: transmembrane transport |
| 36617_at | ID1 | 0000122: negative regulation of transcription from RNA polymerase II promoter; 0001525: angiogenesis; 0001886: endothelial cell morphogenesis; 0006915: apoptosis; 0007179: transforming growth factor beta receptor signaling pathway; 0007275: multicellular organismal development; 0007507: heart development; 0010621: negative regulation of transcription by transcription factor localization; 0016481: negative regulation of transcription; 0030509: BMP signaling pathway; 0031648: protein destabilization; 0032963: collagen metabolic process; 0043408: regulation of MAPKKK cascade; 0043433: negative regulation of transcription factor activity; 0043534: blood vessel endothelial cell migration; 0045449: regulation of transcription; 0045765: regulation of angiogenesis; 0046677: response to antibiotic; 0048514: blood vessel morphogenesis; 0051789: response to protein stimulus; 0060425: lung morphogenesis; 0060426: lung vasculature development |
| 34745_at | RAPGEF2 | 0000165: MAPKKK cascade; 0007165: signal transduction; 0007264: small GTPase mediated signal transduction; 0019933: cAMP-mediated signaling; 0023034: intracellular signaling pathway; 0051056: regulation of small GTPase mediated signal transduction |
| 37569_at | PDCD6 | 0006350: transcription; 0006355: regulation of transcription, DNA-dependent; 0006915: apoptosis; 0006919: activation of caspase activity; 0007165: signal transduction; 0008624: induction of apoptosis by extracellular signals; 0045449: regulation of transcription; 0051592: response to calcium ion; 0051592: response to calcium ion |
| 41407_at | RDBP | 0006350: transcription; 0045449: regulation of transcription |
| 40051_at | TRAM2 | 0006810: transport; 0015031: protein transport; 0032964: collagen biosynthetic process; 0055085: transmembrane transport |
| 1385_at | TGFBI | 0007155: cell adhesion; 0007162: negative regulation of cell adhesion; 0007601: visual perception; 0008283: cell proliferation; 0030198: extracellular matrix organization; 0050896: response to stimulus |
| 1310_at | PSMB2 | 0010243: response to organic nitrogen; 0014070: response to organic cyclic substance; 0031145: anaphase-promoting complex-dependent proteasomal ubiquitin-dependent protein catabolic process; 0044419: interspecies interaction between organisms; 0051436: negative regulation of ubiquitin-protein ligase activity involved in mitotic cell cycle; 0051437: positive regulation of ubiquitin-protein ligase activity involved in mitotic cell cycle; 0051603: proteolysis involved in cellular protein catabolic process |
| 33877_s_at | MTDH | 0000122: negative regulation of transcription from RNA polymerase II promoter; 0051092: positive regulation of NF-kappaB transcription factor activity |
| 36633_at | AKAP10 | 0007165: signal transduction; 0008104: protein localization |
| 39665_at | GLRB | 0001964: startle response; 0006810: transport; 0006811: ion transport; 0006821: chloride transport; 0007218: neuropeptide signaling pathway; 0007268: synaptic transmission; 0007340: acrosome reaction; 0007399: nervous system development; 0007601: visual perception; 0007628: adult walking behavior; 0042391: regulation of membrane potential; 0050905: neuromuscular process; 0051291: protein heterooligomerization; 0060012: synaptic transmission, glycinergic; 0060013: righting reflex |
| 38420_at | COL5A2 | 0001501: skeletal system development; 0001503: ossification; 0007165: signal transduction; 0030199: collagen fibril; 0030199: collagen fibril organization; 0043588: skin development; 0043588: skin development; 0043588: skin development; 0048592: eye morphogenesis |
| 39139_at | SEC11A | 0006465: signal peptide processing; 0006508: proteolysis |
| 38770_at | ADAM12 | 0006508: proteolysis; 0007155: cell adhesion; 0007520: myoblast fusion |
| 891_at | YY1 | 0006350: transcription; 0006355: regulation of transcription, DNA-dependent; 0006357: regulation of transcription from RNA polymerase II promoter; 0009952: anterior/posterior pattern formation; 0010553: negative regulation of gene-specific transcription from RNA polymerase II promoter; 0045449: regulation of transcription; 0048593: camera-type eye morphogenesis |
| 34880_at | RBM42 | 0000166: nucleotide binding; 0003676: nucleic acid binding; 0003723: RNA binding |
| 39686_g_at | ATXN10 | 0007399: nervous system development; 0008219: cell death; 0031175: neuron projection development |
| 34330_at | COX7A2L | 0004129: cytochrome-c oxidase activity; 0009055: electron carrier activity |
| 37391_at | CTSL1 | 0006508: proteolysis |
| 31866_at | PAF1 | 0006350: transcription; 0010390: histone monoubiquitination; 0033523: histone H2B ubiquitination; 0045449: regulation of transcription |
| 32117_at | AATF | 0006915: apoptosis; 0006916: anti-apoptosis; 0006974: response to DNA damage stimulus; 0007155: cell adhesion; 0007346: regulation of mitotic cell cycle; 0008624: induction of apoptosis by extracellular signals; 0010671: negative regulation of oxygen and reactive oxygen species metabolic process; 0032929: negative regulation of superoxide anion generation; 0040016: embryonic cleavage; 0042254: ribosome biogenesis; 0042985: negative regulation of amyloid precursor protein biosynthetic process; 0045941: positive regulation of transcription; 0045944: positive regulation of transcription from RNA polymerase II promoter |
| 34677_f_at | LOC162632; LOC220594; USP32; USP6 | 0006464: protein modification process; 0006511: ubiquitin-dependent protein catabolic process; 0016579: protein deubiquitination; 0016579: protein deubiquitination; 0032313: regulation of Rab GTPase activity; 0060627: regulation of vesicle-mediated transport |
| 38257_at | NDUFS8 | 0006120: mitochondrial electron transport, NADH to ubiquinone; 0006810: transport; 0006979: response to oxidative stress; 0019684: photosynthesis, light reaction; 0022900: electron transport chain; 0032981: mitochondrial respiratory chain complex I assembly; 0055114: oxidation reduction |
| 39795_at | AP2M1 | 0006810: transport; 0006886: intracellular protein transport; 0015031: protein transport; 0016044: cellular membrane organization; 0016192: vesicle-mediated transport; 0050690: regulation of defense response to virus by virus |
| 491_at | PTPRG | 0006470: protein dephosphorylation; 0007169: transmembrane receptor protein tyrosine kinase signaling pathway; 0007420: brain development; 0010977: negative regulation of neuron projection development; 0016311: dephosphorylation |
| 41193_at | DUSP6 | 0000188: inactivation of MAPK activity; 0001933: negative regulation of protein phosphorylation; 0006470: protein dephosphorylation; 0014070: response to organic cyclic substance; 0016311: dephosphorylation; 0030154: cell differentiation; 0035335: peptidyl-tyrosine dephosphorylation; 0042493: response to drug; 0043065: positive regulation of apoptosis; 0051409: response to nitrosative stress; 0060420: regulation of heart growth; 0070373: negative regulation of ERK1 and ERK2 cascade; 0070848: response to growth factor stimulus |
| 1444_at | PSMD9 | 0006511: ubiquitin-dependent protein catabolic process; 0031145: anaphase-promoting complex-dependent proteasomal ubiquitin-dependent protein catabolic process; 0032024: positive regulation of insulin secretion; 0045941: positive regulation of transcription; 0046676: negative regulation of insulin secretion; 0051436: negative regulation of ubiquitin-protein ligase activity involved in mitotic cell cycle; 0051437: positive regulation of ubiquitin-protein ligase activity involved in mitotic cell cycle; 0070682: proteasome regulatory particle assembly |
| 31950_at | PABPC1 | 0006378: mRNA polyadenylation; 0006397: mRNA processing; 0008380: RNA splicing; 0048255: mRNA stabilization |
| 41126_at | SLC1A4 | 0006810: transport; 0006835: dicarboxylic acid transport; 0006865: amino acid transport; 0015808: L-alanine transport; 0015811: L-cystine transport; 0015824: proline transport; 0015825: L-serine transport; 0015826: threonine transport; 0034589: hydroxyproline transport; 0035249: synaptic transmission, glutamatergic; 0050890: cognition |
| 41333_at | ACAP2 | 0032312: regulation of ARF GTPase activity |
| 41690_at | ARID5B | 0001822: kidney development; 0006350: transcription; 0006807: nitrogen compound metabolic process; 0009791: post-embryonic development; 0010761: fibroblast migration; 0035264: multicellular organism growth; 0045449: regulation of transcription; 0045892: negative regulation of transcription, DNA-dependent; 0048008: platelet-derived growth factor receptor signaling pathway; 0048644: muscle organ morphogenesis; 0048705: skeletal system morphogenesis; 0060021: palate development; 0060325: face morphogenesis |
| 39005_s_at | LTN1 | 0005488: binding; 0005515: protein binding; 0008270: zinc ion binding; 0016874: ligase activity; 0046872: metal ion binding |
| 37347_at | CKS1B | 0000079: regulation of cyclin-dependent protein kinase activity; 0007049: cell cycle; 0008283: cell proliferation; 0051301: cell division |
| 33348_at | TCF12 | 0006350: transcription; 0006357: regulation of transcription from RNA polymerase II promoter; 0006955: immune response; 0007275: multicellular organismal development; 0007517: muscle organ development; 0045449: regulation of transcription |
| 33362_at | CDC42EP3 | 0007165: signal transduction; 0008360: regulation of cell shape |
| 1295_at | RELA | 0001889: liver development; 0001942: hair follicle development; 0006350: transcription; 0006355: regulation of transcription, DNA-dependent; 0006916: anti-apoptosis; 0006950: response to stress; 0006952: defense response; 0006954: inflammatory response; 0006968: cellular defense response; 0007568: aging; 0008284: positive regulation of cell proliferation; 0009612: response to mechanical stimulus; 0009617: response to bacterium; 0009887: organ morphogenesis; 0010033: response to organic substance; 0010035: response to inorganic substance; 0010224: response to UV-B; 0014040: positive regulation of Schwann cell differentiation; 0014070: response to organic cyclic substance; 0019221: cytokine-mediated signaling pathway; 0031293: membrane protein intracellular domain proteolysis; 0032332: positive regulation of chondrocyte differentiation; 0032495: response to muramyl dipeptide; 0032496: response to lipopolysaccharide; 0032570: response to progesterone stimulus; 0032582: negative regulation of gene-specific transcription; 0032868: response to insulin stimulus; 0033590: response to cobalamin; 0034097: response to cytokine stimulus; 0042177: negative regulation of protein catabolic process; 0042493: response to drug; 0042542: response to hydrogen peroxide; 0043123: positive regulation of I-kappaB kinase/NF-kappaB cascade; 0043200: response to amino acid stimulus; 0043278: response to morphine; 0044419: interspecies interaction between organisms; 0045084: positive regulation of interleukin-12 biosynthetic process; 0045449: regulation of transcription; 0045893: positive regulation of transcription, DNA-dependent; 0045944: positive regulation of transcription from RNA polymerase II promoter; 0046627: negative regulation of insulin receptor signaling pathway; 0051092: positive regulation of NF-kappaB transcription factor activity; 0051591: response to cAMP; 0051607: defense response to virus; 0070431: nucleotide-binding oligomerization domain containing 2 signaling pathway; 0070555: response to interleukin-1 |
| 37650_at | MKRN1 | 0003676: nucleic acid binding; 0003682: chromatin binding; 0005515: protein binding; 0008270: zinc ion binding; 0016874: ligase activity; 0046872: metal ion binding |
| 39792_at | HNRNPR | 0006397: mRNA processing; 0008380: RNA splicing |
| 36500_at | NSDHL | 0001942: hair follicle development; 0006694: steroid biosynthetic process; 0006695: cholesterol biosynthetic process; 0007224: smoothened signaling pathway; 0008152: metabolic process; 0008203: cholesterol metabolic process; 0008610: lipid biosynthetic process; 0016126: sterol biosynthetic process; 0055114: oxidation reduction; 0060716: labyrinthine layer blood vessel development |
| 37685_at | PICALM | 0006461: protein complex assembly; 0006897: endocytosis; 0006898: receptor-mediated endocytosis; 0006898: receptor-mediated endocytosis; 0007409: axonogenesis; 0016192: vesicle-mediated transport; 0016197: endosome transport; 0030097: hemopoiesis; 0030100: regulation of endocytosis; 0031623: receptor internalization; 0032880: regulation of protein localization; 0045941: positive regulation of transcription; 0048261: negative regulation of receptor-mediated endocytosis; 0048268: clathrin coat assembly; 0048813: dendrite morphogenesis |
| 1140_at | ITGAE | 0007155: cell adhesion; 0007229: integrin-mediated signaling pathway |
| 41342_at | RANBP1 | 0007051: spindle organization; 0007165: signal transduction; 0046604: positive regulation of mitotic centrosome separation; 0046907: intracellular transport |
| 39839_at | CSDA | 0000122: negative regulation of transcription from RNA polymerase II promoter; 0001701: in utero embryonic development; 0006350: transcription; 0006355: regulation of transcription, DNA-dependent; 0007283: spermatogenesis; 0008584: male gonad development; 0009409: response to cold; 0009566: fertilization; 0031100: organ regeneration; 0043066: negative regulation of apoptosis; 0045449: regulation of transcription; 0046622: positive regulation of organ growth; 0048642: negative regulation of skeletal muscle tissue development |
| 41547_at | BUB3 | 0000070: mitotic sister chromatid segregation; 0007049: cell cycle; 0007059: chromosome segregation; 0007067: mitosis; 0007126: meiosis; 0008608: attachment of spindle microtubules to kinetochore; 0031145: anaphase-promoting complex-dependent proteasomal ubiquitin-dependent protein catabolic process; 0051301: cell division; 0051436: negative regulation of ubiquitin-protein ligase activity involved in mitotic cell cycle; 0051983: regulation of chromosome segregation; 0071173: spindle assembly checkpoint |
| 39755_at | XBP1 | 0006350: transcription; 0006355: regulation of transcription, DNA-dependent; 0006955: immune response; 0045449: regulation of transcription |
| 36014_at | GPR126 | 0007165: signal transduction; 0007166: cell surface receptor linked signaling pathway; 0007186: G-protein coupled receptor protein signaling pathway; 0007218: neuropeptide signaling pathway |
| 34783_s_at | BUB3 | 0000070: mitotic sister chromatid segregation; 0007049: cell cycle; 0007059: chromosome segregation; 0007067: mitosis; 0007126: meiosis; 0008608: attachment of spindle microtubules to kinetochore; 0031145: anaphase-promoting complex-dependent proteasomal ubiquitin-dependent protein catabolic process; 0051301: cell division; 0051436: negative regulation of ubiquitin-protein ligase activity involved in mitotic cell cycle; 0051983: regulation of chromosome segregation; 0071173: spindle assembly checkpoint |
| 288_s_at | LBR | 0003676: nucleic acid binding; 0003677: DNA binding; 0004872: receptor activity; 0005515: protein binding; 0005521: lamin binding; 0050613: delta14-sterol reductase activity; 0070087: chromo shadow domain binding |
| 40859_at | ZC3H14 | 0005634: nucleus |
| 32134_at | TES | 0008270: zinc ion binding; 0046872: metal ion binding |
| 38985_at | LEPROTL1 | 0016020: membrane; 0016021: integral to membrane |
| 35779_at | VPS45 | 0006810: transport; 0006886: intracellular protein transport; 0006904: vesicle docking involved in exocytosis; 0015031: protein transport; 0016192: vesicle-mediated transport |
| 1695_at | NEDD8 | 0006357: regulation of transcription from RNA polymerase II promoter; 0006464: protein modification process; 0006508: proteolysis; 0006511: ubiquitin-dependent protein catabolic process; 0008104: protein localization; 0009653: anatomical structure morphogenesis; 0014070: response to organic cyclic substance; 0045116: protein neddylation; 0045116: protein neddylation |
| 37679_at | IFRD1 | 0007275: multicellular organismal development; 0007518: myoblast cell fate determination; 0007527: adult somatic muscle development; 0030154: cell differentiation; 0042692: muscle cell differentiation |
| 36013_at | SLC30A9 | 0006289: nucleotide-excision repair; 0006350: transcription; 0006810: transport; 0006811: ion transport; 0006812: cation transport; 0006829: zinc ion transport; 0045449: regulation of transcription; 0055085: transmembrane transport |
| 36972_at | TMED2 | 0006810: transport; 0015031: protein transport; 0016192: vesicle-mediated transport |
| 37046_at | PSMA5 | 0006511: ubiquitin-dependent protein catabolic process; 0031145: anaphase-promoting complex-dependent proteasomal ubiquitin-dependent protein catabolic process; 0051436: negative regulation of ubiquitin-protein ligase activity involved in mitotic cell cycle; 0051437: positive regulation of ubiquitin-protein ligase activity involved in mitotic cell cycle; 0051603: proteolysis involved in cellular protein catabolic process |
| 40576_f_at | HNRPDL | 0006350: transcription; 0006396: RNA processing; 0045449: regulation of transcription |
| 33458_r_at | CALM2 | 0007186: G-protein coupled receptor protein signaling pathway; 0010880: regulation of release of sequestered calcium ion into cytosol by sarcoplasmic reticulum; 0032465: regulation of cytokinesis; 0051592: response to calcium ion; 0060315: negative regulation of ryanodine-sensitive calcium-release channel activity; 0060316: positive regulation of ryanodine-sensitive calcium-release channel activity |
| 34433_at | DOK1 | 0007165: signal transduction; 0007166: cell surface receptor linked signaling pathway; 0007169: transmembrane receptor protein tyrosine kinase signaling pathway; 0007169: transmembrane receptor protein tyrosine kinase signaling pathway; 0007243: intracellular protein kinase cascade; 0007265: Ras protein signal transduction |
| 38121_at | WARS | 0001525: angiogenesis; 0006412: translation; 0006418: tRNA aminoacylation for protein translation; 0006436: tryptophanyl-tRNA aminoacylation; 0008285: negative regulation of cell proliferation; 0045765: regulation of angiogenesis |

**Upregulated genes in the patient without AP-1 consensus sequence**

| **Probe Set ID** | **Gene Symbol** | **Gene Ontology Biological Process** |
| --- | --- | --- |
| 1451_s_at | POSTN | 0001501: skeletal system development; 0007155: cell adhesion |
| 40698_at | CLEC2B | 0005887: integral to plasma membrane; 0016020: membrane; 0016021: integral to membrane |
| 40074_at | MTHFD2 | 0006730: one-carbon metabolic process; 0008152: metabolic process; 0009396: folic acid and derivative biosynthetic process; 0046653: tetrahydrofolate metabolic process; 0055114: oxidation reduction |
| 36861_at | MXRA5 | 0005576: extracellular region |
| 39337_at | H2AFZ | 0006334: nucleosome assembly |
| 36582_g_at | GARS | 0006412: translation; 0006418: tRNA aminoacylation for protein translation; 0006426: glycyl-tRNA aminoacylation; 0008219: cell death; 0015966: diadenosine tetraphosphate biosynthetic process; 0045055: regulated secretory pathway |
| 40916_at | BEX4 | 0005634: nucleus; 0005737: cytoplasm |
| 40161_at | COMP | 0001501: skeletal system development; 0003417: growth plate cartilage development; 0006915: apoptosis; 0006916: anti-apoptosis; 0007155: cell adhesion; 0009887: organ morphogenesis; 0060173: limb development |
| 39354_at | PRDX6 | 0006979: response to oxidative stress; 0009395: phospholipid catabolic process; 0016042: lipid catabolic process; 0042744: hydrogen peroxide catabolic process; 0045454: cell redox homeostasis; 0055114: oxidation reduction |
| 34390_at | P4HA2 | 0018401: peptidyl-proline hydroxylation to 4-hydroxy-L-proline; 0055114: oxidation reduction |
| 36581_at | GARS | 0006412: translation; 0006418: tRNA aminoacylation for protein translation; 0006426: glycyl-tRNA aminoacylation; 0008219: cell death; 0015966: diadenosine tetraphosphate biosynthetic process; 0045055: regulated secretory pathway |
| 37459_at | COL8A1 | 0001525: angiogenesis; 0007155: cell adhesion; 0007525: somatic muscle development; 0010811: positive regulation of cell-substrate adhesion; 0048593: camera-type eye morphogenesis; 0050673: epithelial cell proliferation |
| 1450_g_at | PSMA4 | 0006511: ubiquitin-dependent protein catabolic process; 0031145: anaphase-promoting complex-dependent proteasomal ubiquitin-dependent protein catabolic process; 0044419: interspecies interaction between organisms; 0051436: negative regulation of ubiquitin-protein ligase activity involved in mitotic cell cycle; 0051437: positive regulation of ubiquitin-protein ligase activity involved in mitotic cell cycle; 0051603: proteolysis involved in cellular protein catabolic process |
| 35814_at | EIF3M | 0006412: translation; 0006413: translational initiation |
| 40813_at | SLC5A3 | 0006020: inositol metabolic process; 0006810: transport; 0006811: ion transport; 0006814: sodium ion transport; 0007422: peripheral nervous system development; 0015798: myo-inositol transport; 0043576: regulation of respiratory gaseous exchange; 0055085: transmembrane transport |
| 35956_s_at | PSG4; PSG7 | 0006952: defense response; 0007565: female pregnancy |
| 37050_r_at | TOMM34 | 0006626: protein targeting to mitochondrion |
| 32825_at | PRMT1 | 0001701: in utero embryonic development; 0006479: protein methylation; 0007166: cell surface receptor linked signaling pathway |
| 40103_at | EZR | 0007016: cytoskeletal anchoring at plasma membrane; 0007159: leukocyte cell-cell adhesion; 0008360: regulation of cell shape; 0022614: membrane to membrane docking; 0030855: epithelial cell differentiation; 0035088: establishment or maintenance of apical/basal cell polarity; 0051017: actin filament bundle assembly |
| 35367_at | LGALS3 | 0001501: skeletal system development; 0030154: cell differentiation; 0030198: extracellular matrix organization |
| 38479_at | ANP32B | 0005634: nucleus |
| 41237_at | HLA-A | 0002474: antigen processing and presentation of peptide antigen via MHC class I; 0006955: immune response; 0019882: antigen processing and presentation; 0044419: interspecies interaction between organisms |
| 39733_at | HERPUD1 | 0006874: cellular calcium ion homeostasis; 0006950: response to stress; 0006986: response to unfolded protein; 0030968: endoplasmic reticulum unfolded protein response; 0043154: negative regulation of caspase activity |
| 38679_g_at | SNRPE | 0000245: spliceosome assembly; 0000387: spliceosomal snRNP assembly; 0006397: mRNA processing; 0008380: RNA splicing; |
| 38038_at | LUM | 0007601: visual perception; 0030199: collagen fibril organization |
| 37749_at | MEST | 0007498: mesoderm development; 0010883: regulation of lipid storage; 0032526: response to retinoic acid |
| 41523_at | RAB32 | 0007264: small GTPase mediated signal transduction; 0015031: protein transport |
| 36171_at | SUB1 | 0006350: transcription; 0006355: regulation of transcription, DNA-dependent; 0006357: regulation of transcription from RNA polymerase II promoter; 0045449: regulation of transcription |
| 40162_s_at | COMP | 0001501: skeletal system development; 0003417: growth plate cartilage development; 0006915: apoptosis; 0006916: anti-apoptosis; 0007155: cell adhesion; 0009887: organ morphogenesis; 0060173: limb development |
| 40774_at | CCT3 | 0006457: protein folding; 0044267: cellular protein metabolic process |
| 195_s_at | CASP4 | 0006508: proteolysis; 0006915: apoptosis; 0006917: induction of apoptosis; 0042981: regulation of apoptosis |
| 34368_at | HDAC2 | 0006338: chromatin remodeling; 0006344: maintenance of chromatin silencing; 0006350: transcription; 0008284: positive regulation of cell proliferation; 0010552: positive regulation of gene-specific transcription from RNA polymerase II promoter; 0010553: negative regulation of gene-specific transcription from RNA polymerase II promoter; 0010870: positive regulation of receptor biosynthetic process; 0010977: negative regulation of neuron projection development; 0016481: negative regulation of transcription; 0016568: chromatin modification; 0016575: histone deacetylation; 0021766: hippocampus development; 0030182: neuron differentiation; 0032967: positive regulation of collagen biosynthetic process; 0034605: cellular response to heat; 0042220: response to cocaine; 0042493: response to drug; 0043433: negative regulation of transcription factor activity; 0045347: negative regulation of MHC class II biosynthetic process; 0045449: regulation of transcription; 0045786: negative regulation of cell cycle; 0045862: positive regulation of proteolysis |
| 39169_at | SEC61G | 0006605: protein targeting; 0006810: transport; 0006886: intracellular protein transport; 0015031: protein transport; 0045047: protein targeting to ER; 0055085: transmembrane transport |
| 36851_g_at | TUSC3 | 0018279: protein N-linked glycosylation via asparagine; 0045454: cell redox homeostasis |
| 37692_at | DBI | 0006810: transport |
| 36088_at | PSMG1 | 0043248: proteasome assembly |
| 32510_at | AKR7A2 | 0005975: carbohydrate metabolic process; 0006081: cellular aldehyde metabolic process; 0055114: oxidation reduction |
| 36159_s_at | PRNP | 0006878: cellular copper ion homeostasis; 0006916: anti-apoptosis; 0006979: response to oxidative stress; 0006979: response to oxidative stress; 0007049: cell cycle; 0007050: cell cycle arrest; 0008152: metabolic process; 0043066: negative regulation of apoptosis; 0046686: response to cadmium ion; 0046688: response to copper ion; 0051260: protein homooligomerization |
| 41403_at | SNRPF | 0000387: spliceosomal snRNP assembly; 0006397: mRNA processing; 0008380: RNA splicing |
| 36985_at | IDI1 | 0006694: steroid biosynthetic process; 0006695: cholesterol biosynthetic process; 0008299: isoprenoid biosynthetic process; 0008610: lipid biosynthetic process; 0016117: carotenoid biosynthetic process; 0016126: sterol biosynthetic process |
| 37720_at | HSPD1 | 0002368: B cell cytokine production; 0002755: MyD88-dependent toll-like receptor signaling pathway; 0002842: positive regulation of T cell mediated immune response to tumor cell; 0006457: protein folding; 0006458: 'de novo' protein folding; 0006919: activation of caspase activity; 0006950: response to stress; 0006986: response to unfolded protein; 0008219: cell death; 0032727: positive regulation of interferon-alpha production; 0032727: positive regulation of interferon-alpha production; 0032729: positive regulation of interferon-gamma production; 0032733: positive regulation of interleukin-10 production; 0032735: positive regulation of interleukin-12 production; 0032755: positive regulation of interleukin-6 production; 0042026: protein refolding; 0042100: B cell proliferation; 0042110: T cell activation; 0042113: B cell activation; 0043032: positive regulation of macrophage activation; 0043065: positive regulation of apoptosis; 0043066: negative regulation of apoptosis; 0044267: cellular protein metabolic process; 0044419: interspecies interaction between organisms; 0048291: isotype switching to IgG isotypes; 0050821: protein stabilization; 0050870: positive regulation of T cell activation; 0051131: chaperone-mediated protein complex assembly; 0051604: protein maturation |
| 41250_at | AIMP2 | 0006412: translation; 0006915: apoptosis; 0007275: multicellular organismal development; 0030154: cell differentiation |
| 33113_at | CITED2 | 0000122: negative regulation of transcription from RNA polymerase II promoter; 0001666: response to hypoxia; 0001889: liver development; 0006350: transcription; 0006916: anti-apoptosis; 0006950: response to stress; 0007368: determination of left/right symmetry; 0007507: heart development; 0008283: cell proliferation; 0010552: positive regulation of gene-specific transcription from RNA polymerase II promoter; 0010628: positive regulation of gene expression; 0010629: negative regulation of gene expression; 0016481: negative regulation of transcription; 0022409: positive regulation of cell-cell adhesion; 0030336: negative regulation of cell migration; 0030511: positive regulation of transforming growth factor beta receptor signaling pathway; 0034405: response to fluid shear stress; 0045449: regulation of transcription; 0045787: positive regulation of cell cycle;:/ 0045893: positive regulation of transcription, DNA-dependent; 0045941: positive regulation of transcription |
| 1073_at | TCEA1 | 0006350: transcription; 0006354: RNA elongation; 0006355: regulation of transcription, DNA-dependent; 0006357: regulation of transcription from RNA polymerase II promoter; 0006366: transcription from RNA polymerase II promoter; 0030218: erythrocyte differentiation; 0045449: regulation of transcription; 0045893: positive regulation of transcription, DNA-dependent; 0045944: positive regulation of transcription from RNA polymerase II promoter |
| 36172_s_at | AP3D1 | 0006726: eye pigment biosynthetic process; 0006810: transport; 0006886: intracellular protein transport; 0015031: protein transport; 0016192: vesicle-mediated transport; 0019882: antigen processing and presentation; 0048007: antigen processing and presentation, exogenous lipid antigen via MHC class Ib; 0051138: positive regulation of NK T cell differentiation; 0061088: regulation of sequestering of zinc ion |
| 41824_at | UTP18 | 0006364: rRNA processing |
| 38029_at | SLC3A2 | 0005975: carbohydrate metabolic process; 0006810: transport; 0006816: calcium ion transport; 0006865: amino acid transport; 0015804: neutral amino acid transport; 0015827: tryptophan transport; 0016049: cell growth; 0060356: leucine import |
| 36654_s_at | HNRNPA2B1 | 0000398: nuclear mRNA splicing, via spliceosome; 0006397: mRNA processing; 0008380: RNA splicing |
| 39173_at | FBL | 0006364: rRNA processing; 0007165: signal transduction; 0008033: tRNA processing; 0008285: negative regulation of cell proliferation; 0016055: Wnt receptor signaling pathway; 0016074: snoRNA metabolic process; 0033211: adiponectin-mediated signaling pathway; 0043623: cellular protein complex assembly; 0051101: regulation of DNA binding |
| 37640_at | HPRT1 | 0001975: response to amphetamine; 0006164: purine nucleotide biosynthetic process; 0006166: purine ribonucleoside salvage; 0006168: adenine salvage; 0006178: guanine salvage; 0007610: behavior; 0007625: grooming behavior; 0009116: nucleoside metabolic process; 0019835: cytolysis; 0021756: striatum development; 0021895: cerebral cortex neuron differentiation; 0021954: central nervous system neuron development; 0032263: GMP salvage; 0032264: IMP salvage; 0042417: dopamine metabolic process; 0045964: positive regulation of dopamine metabolic process; 0046083: adenine metabolic process; 0046100: hypoxanthine metabolic process; 0046651: lymphocyte proliferation; 0048813: dendrite morphogenesis; 0051289: protein homotetramerization |
| 39722_at | RNF187 | 0006350: transcription; 0006366: transcription from RNA polymerase II promoter; 0010553: negative regulation of gene-specific transcription from RNA polymerase II promoter; 0016568: chromatin modification; 0045449: regulation of transcription; 0046329: negative regulation of JNK cascade; 0051225: spindle assembly |
| 1945_at | CCNB1 | 0000086: G2/M transition of mitotic cell cycle; 0000236: mitotic prometaphase; 0000278: mitotic cell cycle; 0001556: oocyte maturation; 0001701: in utero embryonic development; 0001933: negative regulation of protein phosphorylation; 0006461: protein complex assembly; 0006468: protein phosphorylation; 0007049: cell cycle; 0007067: mitosis; 0007080: mitotic metaphase plate congression; 0007283: spermatogenesis; 0009612: response to mechanical stimulus; 0009636: response to toxin; 0010629: negative regulation of gene expression; 0031145: anaphase-promoting complex-dependent proteasomal ubiquitin-dependent protein catabolic process; 0031442: positive regulation of mRNA 3'-end processing; 0033129: positive regulation of histone phosphorylation; 0042246: tissue regeneration; 0042493: response to drug; 0043148: mitotic spindle stabilization; 0045931: positive regulation of mitotic cell cycle; 0046680: response to DDT; 0048565: digestive tract development; 0051301: cell division; 0051437: positive regulation of ubiquitin-protein ligase activity involved in mitotic cell cycle; 0051726: regulation of cell cycle; 0051987: positive regulation of attachment of spindle microtubules to kinetochore; 0055015: ventricular cardiac muscle cell development; 0060045: positive regulation of cardiac muscle cell proliferation; 0060623: regulation of chromosome condensation; 0071174: mitotic cell cycle spindle checkpoint; 0071283: cellular response to iron(III) ion; 0071398: cellular response to fatty acid; 0071407: cellular response to organic cyclic substance; 0071445: cellular response to protein stimulus; 0071456: cellular response to hypoxia |
| 31492_at | EIF3K | 0006412: translation; 0006413: translational initiation; 0006446: regulation of translational initiation |
| 36975_at | TMEM66 | 0016020: membrane; 0016021: integral to membrane |
| 35792_at | MGLL | 0006629: lipid metabolic process; 0006633: fatty acid biosynthetic process; 0006954: inflammatory response; 0008610: lipid biosynthetic process; 0016042: lipid catabolic process; 0019369: arachidonic acid metabolic process; 0035466: regulation of signaling pathway; 0046464: acylglycerol catabolic process; 0050727: regulation of inflammatory response; 0051930: regulation of sensory perception of pain; 2000124: regulation of endocannabinoid signaling pathway |
| 39092_at | H2AFV | 0006334: nucleosome assembly |
| 41765_at | RPL35 | 0006412: translation; 0006414: translational elongation |
| 38368_at | DUT | 0006139: nucleobase, nucleoside, nucleotide and nucleic acid metabolic process; 0006260: DNA replication; 0009117: nucleotide metabolic process; 0046080: dUTP metabolic process |
| 39748_at | SLC7A1 | 0006810: transport; 0006865: amino acid transport; 0006865: amino acid transport; 0015809: arginine transport; 0032006: regulation of TOR signaling cascade; 0055085: transmembrane transport |
| 39357_at | EMG1 | 0006364: rRNA processing; 0042254: ribosome biogenesis; 0042274: ribosomal small subunit biogenesis |
| 38916_at | MAMLD1 | 0006350: transcription; 0008584: male gonad development; 0045449: regulation of transcription |
| 32853_at | TOMM70A | 0006626: protein targeting to mitochondrion |
| 40839_at | UBL3 | 0005622: intracellular; 0005886: plasma membrane; 0016020: membrane |
| 38744_at | SHFM1 | 0006508: proteolysis |
| 36517_at | U2AF1 | 0000398: nuclear mRNA splicing, via spliceosome; 0006397: mRNA processing; 0008380: RNA splicing |
| 1447_at | PSMB1 | 0031145: anaphase-promoting complex-dependent proteasomal ubiquitin-dependent protein catabolic process; 0044419: interspecies interaction between organisms; 0051436: negative regulation of ubiquitin-protein ligase activity involved in mitotic cell cycle; 0051437: positive regulation of ubiquitin-protein ligase activity involved in mitotic cell cycle; 0051603: proteolysis involved in cellular protein catabolic process |
| 1287_at | PARP1 | 0000723: telomere maintenance; 0006259: DNA metabolic process; 0006281: DNA repair;:/ 0006284: base-excision repair; 0006350: transcription; 0006366: transcription from RNA polymerase II promoter; 0006471: protein ADP-ribosylation; 0006974: response to DNA damage stimulus; 0016540: protein autoprocessing; 0032869: cellular response to insulin stimulus; 0040009: regulation of growth rate; 0042769: DNA damage response, detection of DNA damage; 0045449: regulation of transcription |
| 35983_at | WDR18 | na |
| 41242_at | UAP1 | 0006048: UDP-N-acetylglucosamine biosynthetic process; 0006488: dolichol-linked oligosaccharide biosynthetic process; 0008152: metabolic process |
| 35916_s_at | HNRNPA3 | 0006397: mRNA processing; 0008380: RNA splicing |
| 36685_at | AMD1 | 0006596: polyamine biosynthetic process; 0006597: spermine biosynthetic process; 0008295: spermidine biosynthetic process |
| 37717_at | HNRNPM | 0000380: alternative nuclear mRNA splicing, via spliceosome; 0000398: nuclear mRNA splicing, via spliceosome; 0006397: mRNA processing; 0008380: RNA splicing |
| 38404_at | TGM2 | 0001974: blood vessel remodeling; 0006917: induction of apoptosis; 0018149: peptide cross-linking; 0018153: isopeptide cross-linking via N6-(L-isoglutamyl)-L-lysine; 0043123: positive regulation of I-kappaB kinase/NF-kappaB cascade; 0043277: apoptotic cell clearance; 0045785: positive regulation of cell adhesion; 0048661: positive regulation of smooth muscle cell proliferation; 0050729: positive regulation of inflammatory response; 0051260: protein homooligomerization; 0051482: elevation of cytosolic calcium ion concentration involved in G-protein signaling coupled to IP3 second messenger; 0060445: branching involved in salivary gland morphogenesis; 0060662: salivary gland cavitation |
| 34736_at | CCNB1 | 0000086: G2/M transition of mitotic cell cycle; 0000236: mitotic prometaphase; 0000278: mitotic cell cycle; 0001556: oocyte maturation; 0001701: in utero embryonic development; 0001933: negative regulation of protein phosphorylation; 0006461: protein complex assembly; 0006468: protein phosphorylation; 0007049: cell cycle; 0007067: mitosis; 0007080: mitotic metaphase plate congression; 0007283: spermatogenesis; 0009612: response to mechanical stimulus; 0009636: response to toxin; 0010629: negative regulation of gene expression; 0031145: anaphase-promoting complex-dependent proteasomal ubiquitin-dependent protein catabolic process; 0031442: positive regulation of mRNA 3'-end processing; 0033129: positive regulation of histone phosphorylation; 0042246: tissue regeneration; 0042493: response to drug; 0043148: mitotic spindle stabilization; 0045931: positive regulation of mitotic cell cycle; 0046680: response to DDT; 0048565: digestive tract development; 0051301: cell division; 0051437: positive regulation of ubiquitin-protein ligase activity involved in mitotic cell cycle; 0051726: regulation of cell cycle; 0051987: positive regulation of attachment of spindle microtubules to kinetochore; 0055015: ventricular cardiac muscle cell development; 0060045: positive regulation of cardiac muscle cell proliferation; 0060623: regulation of chromosome condensation; 0071174: mitotic cell cycle spindle checkpoint; 0071283: cellular response to iron(III) ion; 0071398: cellular response to fatty acid; 0071407: cellular response to organic cyclic substance; 0071445: cellular response to protein stimulus; 0071456: cellular response to hypoxia |
| 39070_at | FSCN1 | 0008283: cell proliferation; 0030036: actin cytoskeleton organization; 0051017: actin filament bundle assembly |
| 40607_at | DPYSL2 | 0001975: response to amphetamine; 0006139: nucleobase, nucleoside, nucleotide and nucleic acid metabolic process; 0007165: signal transduction; 0007275: multicellular organismal development; 0007399: nervous system development; 0007420: brain development; 0014049: positive regulation of glutamate secretion; 0021510: spinal cord development; 0021772: olfactory bulb development; 0030154: cell differentiation; 0042220: response to cocaine; 0042493: response to drug; 0045664: regulation of neuron differentiation; 0048489: synaptic vesicle transport |
| 32563_at | ATP1B3 | 0006754: ATP biosynthetic process; 0006810: transport; 0006811: ion transport; 0006813: potassium ion transport; 0006814: sodium ion transport; 0030001: metal ion transport |
| 40546_s_at | NDUFA2 | 0006120: mitochondrial electron transport, NADH to ubiquinone; 0006810: transport; 0022900: electron transport chain |
| 38708_at | RAN | 0006259: DNA metabolic process; 0006405: RNA export from nucleus; 0006611: protein export from nucleus; 0006810: transport; 0006886: intracellular protein transport; 0006913: nucleocytoplasmic transport; 0007049: cell cycle; 0007052: mitotic spindle organization; 0007067: mitosis; 0007165: signal transduction; 0015031: protein transport; 0030521: androgen receptor signaling pathway; 0044419: interspecies interaction between organisms; 0045893: positive regulation of transcription, DNA-dependent; 0051301: cell division |
| 38275_at | IMP4 | 0006364: rRNA processing; 0006412: translation; 0042254: ribosome biogenesis |
| 706_at | NR3C1 | 0006111: regulation of gluconeogenesis; 0006338: chromatin remodeling; 0006350: transcription; 0006351: transcription, DNA-dependent; 0006355: regulation of transcription, DNA-dependent; 0006366: transcription from RNA polymerase II promoter; 0006366: transcription from RNA polymerase II promoter; 0006916: anti-apoptosis; 0007165: signal transduction; 0008211: glucocorticoid metabolic process; 0016568: chromatin modification; 0021987: cerebral cortex development; 0030325: adrenal gland development; 0030512: negative regulation of transforming growth factor beta receptor signaling pathway; 0031946: regulation of glucocorticoid biosynthetic process; 0042127: regulation of cell proliferation; 0042921: glucocorticoid receptor signaling pathway; 0043525: positive regulation of neuron apoptosis; 0045449: regulation of transcription; 0046685: response to arsenic; 0048096: chromatin-mediated maintenance of transcription; 0051789: response to protein stimulus; 0060603: mammary gland duct morphogenesis; 0071109: superior temporal gyrus development |
| 306_s_at | HMGN1 | 0003677: DNA binding; 0003702: RNA polymerase II transcription factor activity; 0008159: positive transcription elongation factor activity |
| 40417_at | CCT5 | 0006457: protein folding; 0009615: response to virus; 0044267: cellular protein metabolic process |
| 41146_at | PARP1 | 0000723: telomere maintenance; 0006259: DNA metabolic process; 0006281: DNA repair; 0006284: base-excision repair; 0006350: transcription; 0006366: transcription from RNA polymerase II promoter; 0006471: protein ADP-ribosylation; 0006974: response to DNA damage stimulus; 0016540: protein autoprocessing; 0032869: cellular response to insulin stimulus; 0040009: regulation of growth rate; 0042769: DNA damage response, detection of DNA damage; 0045449: regulation of transcription |
| 39088_at | TMEM147 | 0016020: membrane; 0016021: integral to membrane |
| 37673_at | NSMAF | 0006672: ceramide metabolic process; 0006917: induction of apoptosis; 0007165: signal transduction |
| 35300_at | EPRS | 0006412: translation; 0006418: tRNA aminoacylation for protein translation; 0006424: glutamyl-tRNA aminoacylation; 0006433: prolyl-tRNA aminoacylation; 0006461: protein complex assembly; 0043039: tRNA aminoacylation |
| 33819_at | LDHB | 0005975: carbohydrate metabolic process; 0006089: lactate metabolic process; 0006096: glycolysis; 0008152: metabolic process; 0019674: NAD metabolic process; 0044262: cellular carbohydrate metabolic process; 0055114: oxidation reduction |
| 36616_at | DAZAP2 | 0005515: protein binding; 0050699: WW domain binding |
| 33865_at | ZMYND11 | 0000122: negative regulation of transcription from RNA polymerase II promoter; 0006350: transcription; 0007049: cell cycle; 0008283: cell proliferation; 0044419: interspecies interaction between organisms; 0045449: regulation of transcription |
| 31824_at | ME1 | 0005975: carbohydrate metabolic process; 0006108: malate metabolic process; 0006741: NADP biosynthetic process; 0008152: metabolic process; 0009725: response to hormone stimulus; 0009743: response to carbohydrate stimulus; 0055114: oxidation reduction |
| 41749_at | C21orf33 | 0005739: mitochondrion |
| 35153_at | NBN | 0000077: DNA damage checkpoint; 0000723: telomere maintenance; 0001701: in utero embryonic development; 0001832: blastocyst growth; 0006281: DNA repair; 0006302: double-strand break repair; 0006974: response to DNA damage stimulus; 0007049: cell cycle; 0007050: cell cycle arrest; 0007095: mitotic cell cycle G2/M transition DNA damage checkpoint; 0007126: meiosis; 0008283: cell proliferation; 0008284: positive regulation of cell proliferation; 0030174: regulation of DNA-dependent DNA replication initiation; 0030330: DNA damage response, signal transduction by p53 class mediator; 0031575: mitotic cell cycle G1/S transition checkpoint; 0031954: positive regulation of protein autophosphorylation; 0032508: DNA duplex unwinding; 0033674: positive regulation of kinase activity; 0042493: response to drug; 0045190: isotype switching; 0045665: negative regulation of neuron differentiation; 0048145: regulation of fibroblast proliferation; 0050885: neuromuscular process controlling balance |
| 1848_at | RAP1A | 0007165: signal transduction; 0007264: small GTPase mediated signal transduction; 0023034: intracellular signaling pathway |
| 37389_at | C11orf58 | na |
| 32051_at | ALG8 | 0006487: protein N-linked glycosylation; 0006488: dolichol-linked oligosaccharide biosynthetic process |
| 32434_at | MARCKS | 0005737: cytoplasm; 0005813: centrosome; 0005856: cytoskeleton; 0005938: cell cortex; 0015629: actin cytoskeleton; 0016020: membrane; 0042585: germinal vesicle |
| 41510_s_at | HSPA9 | 0006457: protein folding; 0006611: protein export from nucleus; 0006916: anti-apoptosis; 0006950: response to stress |
| 35714_at | PDXK | 0008283: cell proliferation; 0008615: pyridoxine biosynthetic process; 0042816: vitamin B6 metabolic process; 0042823: pyridoxal phosphate biosynthetic process |
| 37462_i_at | SF3A2 | 0000389: nuclear mRNA 3'-splice site recognition; 0000398: nuclear mRNA splicing, via spliceosome; 0006397: mRNA processing; 0008380: RNA splicing |
| 41185_f_at | SUMO2 | 0016925: protein sumoylation; 0032436: positive regulation of proteasomal ubiquitin-dependent protein catabolic process |
| 38605_at | NDUFB1 | 0006120: mitochondrial electron transport, NADH to ubiquinone; 0006810: transport; 0022900: electron transport chain |
| 229_at | CEBPZ | 0006350: transcription; 0006366: transcription from RNA polymerase II promoter; 0045449: regulation of transcription |
| 37726_at | MRPL3 | 0006412: translation |
| 38720_at | CCT7 | 0006457: protein folding; 0044267: cellular protein metabolic process |
| 38676_at | HSPA13 | 0005783: endoplasmic reticulum; 0005792: microsome; 0005792: microsome |
| 38589_i_at | PTMA | 0006350: transcription |
| 39801_at | PLOD3 | 0006464: protein modification process; 0032870: cellular response to hormone stimulus; 0055114: oxidation reduction |
| 38687_at | ANAPC13 | 0007049: cell cycle; 0007067: mitosis; 0051301: cell division; 0070979: protein K11-linked ubiquitination |
| 38974_at | PARK7 | 0001963: synaptic transmission, dopaminergic; 0006950: response to stress; 0006979: response to oxidative stress; 0008219: cell death; 0008344: adult locomotory behavior; 0032091: negative regulation of protein binding; 0042493: response to drug; 0042542: response to hydrogen peroxide; 0042743: hydrogen peroxide metabolic process; 0051583: dopamine uptake; 0051899: membrane depolarization; 0060081: membrane hyperpolarization; 0060765: regulation of androgen receptor signaling pathway |
| 34199_at | SEC24A | 0006810: transport; 0006886: intracellular protein transport; 0006888: ER to Golgi vesicle-mediated transport; 0015031: protein transport; 0016044: cellular membrane organization; 0016192: vesicle-mediated transport |
| 1789_at | COPS5 | 0000338: protein deneddylation; 0006366: transcription from RNA polymerase II promoter; 0006412: translation; 0051726: regulation of cell cycle |
| 34862_at | SCCPDH | 0008152: metabolic process; 0055114: oxidation reduction |
| 40211_at | HNRNPA1 | 0000380: alternative nuclear mRNA splicing, via spliceosome; 0000398: nuclear mRNA splicing, via spliceosome; 0006397: mRNA processing; 0006405: RNA export from nucleus; 0006810: transport; 0008380 RNA splicing; 0044419: interspecies interaction between organisms; 0051028: mRNA transport; 0051168: nuclear export; 0051170: nuclear import |
| 35818_at | CYCS | 0006309: DNA fragmentation involved in apoptotic nuclear change; 0006810: transport; 0006915: apoptosis; 0006915: apoptosis; 0008635: activation of caspase activity by cytochrome c; 0022900: electron transport chain; 0045333: cellular respiration |
| 38837_at | TMX4 | 0006810: transport; 0022900: electron transport chain; 0045454: cell redox homeostasis |
| 1313_at | PSMB7 | 0031145: anaphase-promoting complex-dependent proteasomal ubiquitin-dependent protein catabolic process; 0044419: interspecies interaction between organisms; 0051436: negative regulation of ubiquitin-protein ligase activity involved in mitotic cell cycle; 0051437: positive regulation of ubiquitin-protein ligase activity involved in mitotic cell cycle; 0051603: proteolysis involved in cellular protein catabolic process |
| 1052_s_at | CEBPD | 0006350: transcription; 0006355: regulation of transcription, DNA-dependent; 0006366: transcription from RNA polymerase II promoter; 0045449: regulation of transcription |
| 38114_at | RAD21 | 0006281: DNA repair; 0006302: double-strand break repair; 0006310: DNA recombination; 0006915: apoptosis; 0006974: response to DNA damage stimulus; 0007049: cell cycle; 0007059: chromosome segregation; 0007067: mitosis; 0007131: reciprocal meiotic recombination; 0010551: regulation of gene-specific transcription from RNA polymerase II promoter; 0051301: cell division |
| 36624_at | IMPDH2 | 0006164: purine nucleotide biosynthetic process; 0006177: GMP biosynthetic process; 0008152: metabolic process; 0009168: purine ribonucleoside monophosphate biosynthetic process; 0046651: lymphocyte proliferation; 0055114: oxidation reduction |
| 339_at | CAV2 | 0001937: negative regulation of endothelial cell proliferation; 0006906: vesicle fusion; 0007005: mitochondrion organization 0007029: endoplasmic reticulum organization; 0007088: regulation of mitosis; 0007268: synaptic transmission; 0008285: negative regulation of cell proliferation; 0016050: vesicle organization; 0048278: vesicle docking; 0048741: skeletal muscle fiber development; 0051259: protein oligomerization; 0060161: positive regulation of dopamine receptor signaling pathway; 0070836: caveola assembly |
| 36090_at | TBL2 | na |
| 39003_at | PTTG1IP | 0006606: protein import into nucleus |
| 37311_at | TALDO1 | 0005975: carbohydrate metabolic process; 0006002: fructose 6-phosphate metabolic process; 0006098: pentose-phosphate shunt; 0008152: metabolic process; 0009052: pentose-phosphate shunt, non-oxidative branch; 0019682: glyceraldehyde-3-phosphate metabolic process |
| 34825_at | TDP2 | 0006281: DNA repair; 0006302: double-strand break repair; 0006974: response to DNA damage stimulus; 0007166: cell surface receptor linked signaling pathway |
| 39731_at | RBMX | 0006397: mRNA processing; 0008380: RNA splicing |
| 36185_at | AARS | 0001942: hair follicle development; 0006400: tRNA modification; 0006412: translation; 0006419: alanyl-tRNA aminoacylation; 0006419: alanyl-tRNA aminoacylation; 0006457: protein folding; 0008033: tRNA processing; 0021680: cerebellar Purkinje cell layer development; 0030968: endoplasmic reticulum unfolded protein response; 0034620: cellular response to unfolded protein; 0043039: tRNA aminoacylation; 0043200: response to amino acid stimulus; 0043524: negative regulation of neuron apoptosis; 0043588: skin development; 0050885: neuromuscular process controlling balance; 0050905: neuromuscular process |
| 40108_at | BZW1 | 0006350: transcription; 0016070: RNA metabolic process; 0045449: regulation of transcription |
| 32119_at | --- | na |
| 39328_at | HMGCR | 0006694: steroid biosynthetic process; 0006695: cholesterol biosynthetic process; 0007584: response to nutrient; 0008299: isoprenoid biosynthetic process; 0008354: germ cell migration; 0008406: gonad development; 0008542: visual learning; 0008610: lipid biosynthetic process; 0009790: embryo development; 0015936: coenzyme A metabolic process; 0016126: sterol biosynthetic process; 0043407: negative regulation of MAP kinase activity; 0055114: oxidation reduction |
| 40125_at | CANX | 0006457: protein folding; 0007568: aging; 0009306: protein secretion |
| 140_s_at | TRA2B | 0000375: RNA splicing, via transesterification reactions; 0000398: nuclear mRNA splicing, via spliceosome; 0006397: mRNA processing; 0008380: RNA splicing |
| 35804_at | ASH2L | 0006350: transcription; 0006355: regulation of transcription, DNA-dependent; 0006366: transcription from RNA polymerase II promoter; 0006974: response to DNA damage stimulus; 0008284: positive regulation of cell proliferation; 0016568: chromatin modification; 0030097: hemopoiesis; 0043627: response to estrogen stimulus; 0045449: regulation of transcription; 0051568: histone H3-K4 methylation |
| 1884_s_at | PCNA | 0006260: DNA replication; 0006275: regulation of DNA replication; 0006281: DNA repair; 0006287: base-excision repair, gap-filling; 0006297: nucleotide-excision repair, DNA gap filling; 0006298: mismatch repair; 0006886: intracellular protein transport; 0007507: heart development; 0008283: cell proliferation; 0019985: translesion synthesis; 0033993: response to lipid; 0046686: response to cadmium ion; 0048015: phosphoinositide-mediated signaling |
| 38075_at | SYPL1 | 0006810: transport; 0007268: synaptic transmission |
| 36511_at | SACM1L | 0046856: phosphoinositide dephosphorylation |
| 36821_at | FAM171A1 | 0016020: membrane; 0016021: integral to membrane |
| 1011_s_at | YWHAE | 0001764: neuron migration; 0006605: protein targeting; 0006915: apoptosis; 0008624: induction of apoptosis by extracellular signals; 0021766: hippocampus development; 0021987: cerebral cortex development; 0023034: intracellular signaling pathway; 0035308: negative regulation of protein dephosphorylation; 0044419: interspecies interaction between organisms |
| 35172_at | TPST2 | 0006478: peptidyl-tyrosine sulfation |
| 35738_at | HMGN4 | 0003677: DNA binding |
| 38443_at | PTPN11 | 0000077: DNA damage checkpoint; 0000187: activation of MAPK activity; 0006470: protein dephosphorylation; 0006629: lipid metabolic process; 0006641: triglyceride metabolic process; 0007165: signal transduction; 0007409: axonogenesis; 0009755: hormone-mediated signaling pathway; 0009967: positive regulation of signal transduction; 0016311: dephosphorylation; 0035265: organ growth; 0040014: regulation of multicellular organism growth; 0042445: hormone metabolic process; 0042593: glucose homeostasis; 0046676: negative regulation of insulin secretion; 0046825: regulation of protein export from nucleus; 0046887: positive regulation of hormone secretion; 0046888: negative regulation of hormone secretion; 0048011: nerve growth factor receptor signaling pathway; 0048609: multicellular organismal reproductive process; 0051463: negative regulation of cortisol secretion; 0060125: negative regulation of growth hormone secretion |
| 38356_at | FST | 0000122: negative regulation of transcription from RNA polymerase II promoter; 0002244: hemopoietic progenitor cell differentiation; 0007276: gamete generation; 0007389: pattern specification process; 0008585: female gonad development; 0030509: BMP signaling pathway; 0031069: hair follicle morphogenesis; 0032926: negative regulation of activin receptor signaling pathway; 0042475: odontogenesis of dentine-containing tooth; 0043616: keratinocyte proliferation; 0045596: negative regulation of cell differentiation; 0046882: negative regulation of follicle-stimulating hormone secretion; 0051798: positive regulation of hair follicle development |
| 34887_at | RDX | 0051693: actin filament capping |
| 34367_at | PHGDH | 0001816: cytokine production; 0006954: inflammatory response; 0006955: immune response; 0007165: signal transduction; 0008203: cholesterol metabolic process; 0008284: positive regulation of cell proliferation; 0032582: negative regulation of gene-specific transcription; 0042098: T cell proliferation; 0043433: negative regulation of transcription factor activity; 0050710: negative regulation of cytokine secretion; 0051024: positive regulation of immunoglobulin secretion |
| 38060_at | NDUFS5 | 0006120: mitochondrial electron transport, NADH to ubiquinone; 0006412: translation; 0006412: translation; 0006810: transport; 0022900: electron transport chain; 0032981: mitochondrial respiratory chain complex I assembly |
| 40068_at | LOC100510546; STX5 | 0006810: transport; 0006886: intracellular protein transport; 0006903: vesicle targeting; 0016192: vesicle-mediated transport; 0042147: retrograde transport, endosome to Golgi |
| 38485_at | NDUFC1 | 0006120: mitochondrial electron transport, NADH to ubiquinone; 0006810: transport; 0022900: electron transport chain |
| 32548_at | PTGES3 | 0000723: telomere maintenance; 0001516: prostaglandin biosynthetic process; 0006633: fatty acid biosynthetic process; 0007165: signal transduction; 0008610: lipid biosynthetic process; 0070389: chaperone cofactor-dependent protein refolding |
| 37964_at | PCGF3 | 0006350: transcription; 0045449: regulation of transcription |
| 37809_at | HOXA10; HOXA9 | 0001501: skeletal system development; 0006350: transcription; 0006355: regulation of transcription, DNA-dependent; 0006412: translation; 0007275: multicellular organismal development; 0007283: spermatogenesis; 0009952: anterior/posterior pattern formation; 0009954: proximal/distal pattern formation; 0030326: embryonic limb morphogenesis; 0045449: regulation of transcription |
| 949_s_at | PSMC6 | 0006511: ubiquitin-dependent protein catabolic process; 0030163: protein catabolic process; 0031145: anaphase-promoting complex-dependent proteasomal ubiquitin-dependent protein catabolic process; 0051436: negative regulation of ubiquitin-protein ligase activity involved in mitotic cell cycle; 0051437: positive regulation of ubiquitin-protein ligase activity involved in mitotic cell cycle |
| 34819_at | CD164 | 0006955: immune response; 0007155: cell adhesion; 0007157: heterophilic cell-cell adhesion; 0007162: negative regulation of cell adhesion; 0007165: signal transduction; 0007275: multicellular organismal development; 0007517: muscle organ development; 0008285: negative regulation of cell proliferation; 0030097: hemopoiesis |
| 32194_at | CEBPZ | 0006350: transcription; 0006366: transcription from RNA polymerase II promoter; 0045449: regulation of transcription |
| 38681_at | EIF3E | 0000184: nuclear-transcribed mRNA catabolic process, nonsense-mediated decay; 0006412: translation; 0006413: translational initiation; 0006446: regulation of translational initiation; 0045947: negative regulation of translational initiation |
| 36631_at | PRDX3 | 0001893: maternal placenta development; 0006979: response to oxidative stress; 0007005: mitochondrion organization; 0008284: positive regulation of cell proliferation; 0030099: myeloid cell differentiation; 0032496: response to lipopolysaccharide; 0033673: negative regulation of kinase activity; 0034614: cellular response to reactive oxygen species; 0042542: response to hydrogen peroxide; 0042744: hydrogen peroxide catabolic process; 0043066: negative regulation of apoptosis; 0043524: negative regulation of neuron apoptosis; 0045454: cell redox homeostasis; 0051092: positive regulation of NF-kappaB transcription factor activity; 0051881: regulation of mitochondrial membrane potential; 0055114: oxidation reduction |
| 41504_s_at | MAF | 0001816: cytokine production; 0002088: lens development in camera-type eye; 0006350: transcription; 0006351: transcription, DNA-dependent; 0006355: regulation of transcription, DNA-dependent; 0006366: transcription from RNA polymerase II promoter; 0032330: regulation of chondrocyte differentiation; 0045449: regulation of transcription; 0045944: positive regulation of transcription from RNA polymerase II promoter; 0048468: cell development; 0070306: lens fiber cell differentiation |
| 32153_s_at | UBB | 0006915: apoptosis; 0006916: anti-apoptosis; 0008624: induction of apoptosis by extracellular signals; 0031145: anaphase-promoting complex-dependent proteasomal ubiquitin-dependent protein catabolic process; 0051436: negative regulation of ubiquitin-protein ligase activity involved in mitotic cell cycle; 0051437: positive regulation of ubiquitin-protein ligase activity involved in mitotic cell cycle |
| 39719_at | POMZP3; ZP3 | 0007338: single fertilization; 0007339: binding of sperm to zona pellucida |
| 33351_at | EIF1B | 0006412: translation; 0006413: translational initiation; 0006446: regulation of translational initiation |
| 41235_at | ATF4 | 0006094: gluconeogenesis; 0006350: transcription; 0006355: regulation of transcription, DNA-dependent; 0006520: cellular amino acid metabolic process; 0006950: response to stress; 0034976: response to endoplasmic reticulum stress; 0045449: regulation of transcription; 0045944: positive regulation of transcription from RNA polymerase II promoter |
| 36187_at | RNH1 | 0006402: mRNA catabolic process; 0045765: regulation of angiogenesis |
| 40112_at | IDH3B | 0006099: tricarboxylic acid cycle; 0006102: isocitrate metabolic process; 0006103: 2-oxoglutarate metabolic process; 0006734: NADH metabolic process; 0007601: visual perception; 0050896: response to stimulus; 0055114: oxidation reduction |
| 39517_at | C5orf15 | 0016020: membrane; 0016021: integral to membrane |
| 37703_at | RABGGTB | 0006464: protein modification process; 0007601: visual perception |
| 37296_at | ARL1 | 0007030: Golgi organization; 0007264: small GTPase mediated signal transduction; 0048193: Golgi vesicle transport |
| 38820_at | Sep 15 | 0035092: sperm chromatin condensation; 0051084: 'de novo' posttranslational protein folding |
| 36636_at | OAT | 0007601: visual perception |
| 40421_at | PIN1 | 0006457: protein folding; 0007049: cell cycle; 0007088: regulation of mitosis; 0030512: negative regulation of transforming growth factor beta receptor signaling pathway; 0051443: positive regulation of ubiquitin-protein ligase activity; 0060393: regulation of pathway-restricted SMAD protein phosphorylation |
| 837_s_at | ME1 | 0005975: carbohydrate metabolic process; 0006108: malate metabolic process; 0006741: NADP biosynthetic process; 0008152: metabolic process; 0009725: response to hormone stimulus; 0009743: response to carbohydrate stimulus; 0055114: oxidation reduction |
| 37035_at | SERP1 | 0001501: skeletal system development; 0006006: glucose metabolic process; 0006464: protein modification process; 0006486: protein glycosylation; 0006810: transport; 0006950: response to stress; 0007009: plasma membrane organization; 0009791: post-embryonic development; 0010259: multicellular organismal aging; 0015031: protein transport; 0030968: endoplasmic reticulum unfolded protein response; 0032024: positive regulation of insulin secretion; 0045727: positive regulation of translation; 0046622: positive regulation of organ growth; 0048644: muscle organ morphogenesis; 0055085: transmembrane transport; 0060124: positive regulation of growth hormone secretion |
| 39025_at | C4orf46; TOMM7 | 0006626: protein targeting to mitochondrion; 0006810: transport; 0006886: intracellular protein transport; 0015031: protein transport |
| 39727_at | DUSP11 | 0006396: RNA processing; 0006470: protein dephosphorylation; 0016311: dephosphorylation |
| 38814_at | ATP6V1G1 | 0006810: transport; 0006811: ion transport; 0015992: proton transport |
| 36913_at | SLBP | 0006397: mRNA processing; 0006398: histone mRNA 3'-end processing; 0033260: DNA replication involved in S phase; 0033261: regulation of S phase; 0051028: mRNA transport |
| 33150_at | UTP3 | 0007275: multicellular organismal development; 0007420: brain development; 0016458: gene silencing; 0016568: chromatin modification |
| 33456_at | MAPRE1 | 0007049: cell cycle; 0007067: mitosis; 0008283: cell proliferation; 0031115: negative regulation of microtubule polymerization; 0035372: protein localization to microtubule; 0051301: cell division |
| 32564_at | SEC61B | 0000060: protein import into nucleus, translocation; 0006810: transport; 0015031: protein transport; 0030433: ER-associated protein catabolic process; 0030970: retrograde protein transport, ER to cytosol; 0055085: transmembrane transport |
| 40522_at | GLUL | 0006542: glutamine biosynthetic process; 0006807: nitrogen compound metabolic process; 0008283: cell proliferation; 0009749: response to glucose stimulus |
| 34814_at | UBA2 | 0006464: protein modification process; 0008152: metabolic process; 0016925: protein sumoylation |
| 32914_f_at | C19orf29 | 0006397: mRNA processing; 0008380: RNA splicing |
| 38828_s_at | KHSRP | 0000375: RNA splicing, via transesterification reactions; 0006350: transcription; 0006397: mRNA processing; 0006810: transport; 0008380: RNA splicing; 0045449: regulation of transcription; 0051028: mRNA transport |
| 34830_at | VOPP1 | 0006350: transcription; 0045449: regulation of transcription |
| 1102_s_at | NR3C1 | 0006111: regulation of gluconeogenesis; 0006338: chromatin remodeling; 0006350: transcription; 0006351: transcription, DNA-dependent; 0006355: regulation of transcription, DNA-dependent; 0006366: transcription from RNA polymerase II promoter; 0006916: anti-apoptosis; 0007165: signal transduction; 0008211: glucocorticoid metabolic process; 0016568: chromatin modification; 0021987: cerebral cortex development; 0030325: adrenal gland development; 0030512: negative regulation of transforming growth factor beta receptor signaling pathway; 0031946: regulation of glucocorticoid biosynthetic process; 0042127: regulation of cell proliferation; 0042921: glucocorticoid receptor signaling pathway; 0043525: positive regulation of neuron apoptosis; 0045449: regulation of transcription; 0046685: response to arsenic; 0048096: chromatin-mediated maintenance of transcription; 0051789: response to protein stimulus; 0060603: mammary gland duct morphogenesis; 0071109: superior temporal gyrus development |
| 38545_at | INHBB | 0001541: ovarian follicle development; 0006952: defense response; 0009267: cellular response to starvation; 0009605: response to external stimulus; 0030154: cell differentiation; 0032869: cellular response to insulin stimulus; 0032924: activin receptor signaling pathway; 0040007: growth; 0045444: fat cell differentiation; 0046676: negative regulation of insulin secretion; 0046881: positive regulation of follicle-stimulating hormone secretion; 0046882: negative regulation of follicle-stimulating hormone secretion; 0048178: negative regulation of hepatocyte growth factor biosynthetic process; 0060279: positive regulation of ovulation |
| 38288_at | SNAI2 | 0000122: negative regulation of transcription from RNA polymerase II promoter; 0001649: osteoblast differentiation; 0006350: transcription; 0007275: multicellular organismal development; 0007499: ectoderm and mesoderm interaction; 0009314: response to radiation; 0045449: regulation of transcription; 0060070: canonical Wnt receptor signaling pathway |
| 36102_at | VDAC3 | 0006810: transport; 0006811: ion transport; 0006820: anion transport; 0015853: adenine transport; 0055085: transmembrane transport |
| 36687_at | COX7B | 0005739: mitochondrion; 0005743: mitochondrial inner membrane; 0005746: mitochondrial respiratory chain; 0016020: membrane; 0016021: integral to membrane |
| 34340_at | CYB5B | 0006810: transport; 0022900: electron transport chain |
| 41335_at | NSL1 | 0007049: cell cycle; 0007059: chromosome segregation; 0007067: mitosis; 0051301: cell division |
| 40638_at | SFPQ | 0000380: alternative nuclear mRNA splicing, via spliceosome; 0006281: DNA repair; 0006310: DNA recombination; 0006350: transcription; 0006397: mRNA processing; 0006974: response to DNA damage stimulus; 0008380: RNA splicing; 0045449: regulation of transcription |
| 36515_at | GNE | 0005975: carbohydrate metabolic process; 0006047: UDP-N-acetylglucosamine metabolic process; 0006054: N-acetylneuraminate metabolic process; 0007155: cell adhesion; 0009103: lipopolysaccharide biosynthetic process |
| 39416_at | TAX1BP3 | 0007266: Rho protein signal transduction; 0016055: Wnt receptor signaling pathway; 0030178: negative regulation of Wnt receptor signaling pathway |
| 37337_at | SNRPG | 0000245: spliceosome assembly; 0000387: spliceosomal snRNP assembly; 0006397: mRNA processing; 0008380: RNA splicing |
| 36189_at | ILF2 | 0006350: transcription; 0006955: immune response; 0045449: regulation of transcription; 0045893: positive regulation of transcription, DNA-dependent |
| 34299_at | PATZ1 | 0006350: transcription; 0006355: regulation of transcription, DNA-dependent; 0006417: regulation of translation; 0006468: protein phosphorylation; 0006950: response to stress; 0006950: response to stress; 0007283: spermatogenesis; 0008285: negative regulation of cell proliferation; 0008584: male gonad development; 0009605: response to external stimulus; 0010468: regulation of gene expression; 0010999: regulation of eIF2 alpha phosphorylation by heme; 0017148: negative regulation of translation; 0030217: T cell differentiation; 0045449: regulation of transcription; 0045993: negative regulation of translational initiation by iron; 0046501: protoporphyrinogen IX metabolic process; 0046777: protein autophosphorylation; 0046984: regulation of hemoglobin biosynthetic process; 0046986: negative regulation of hemoglobin biosynthetic process |
| 39118_at | DNAJA1 | 0006457: protein folding; 0006986: response to unfolded protein; 0007283: spermatogenesis; 0009408: response to heat; 0030317: sperm motility; 0030521: androgen receptor signaling pathway |
| 41448_at | HOXA10 | 0001501: skeletal system development; 0006350: transcription; 0006355: regulation of transcription, DNA-dependent; 0006412: translation; 0007275: multicellular organismal development; 0007283: spermatogenesis; 0009952: anterior/posterior pattern formation; 0009954: proximal/distal pattern formation; 0030326: embryonic limb morphogenesis; 0045449: regulation of transcription |
| 38473_at | TARS | 0006412: translation; 0006418: tRNA aminoacylation for protein translation; 0006435: threonyl-tRNA aminoacylation; 0043039: tRNA aminoacylation |
| 40822_at | NFATC3 | 0006350: transcription; 0006355: regulation of transcription, DNA-dependent; 0006357: regulation of transcription from RNA polymerase II promoter; 0006366: transcription from RNA polymerase II promoter; 0006954: inflammatory response; 0007507: heart development; 0045333: cellular respiration; 0045449: regulation of transcription; 0045944: positive regulation of transcription from RNA polymerase II promoter |
| 33422_at | SEC13 | 0006810: transport; 0006886: intracellular protein transport; 0006888: ER to Golgi vesicle-mediated transport; 0015031: protein transport; 0016044: cellular membrane organization |
| 36690_at | NR3C1 | 0006111: regulation of gluconeogenesis; 0006338: chromatin remodeling; 0006350: transcription; 0006351: transcription, DNA-dependent; 0006355: regulation of transcription, DNA-dependent; 0006366: transcription from RNA polymerase II promoter; 0006916: anti-apoptosis; 0007165: signal transduction; 0008211: glucocorticoid metabolic process; 0016568: chromatin modification; 0021987: cerebral cortex development; 0030325: adrenal gland development; 0030512: negative regulation of transforming growth factor beta receptor signaling pathway; 0031946: regulation of glucocorticoid biosynthetic process; 0042127: regulation of cell proliferation; 0042921: glucocorticoid receptor signaling pathway; 0043525: positive regulation of neuron apoptosis; 0045449: regulation of transcription; 0046685: response to arsenic; 0048096: chromatin-mediated maintenance of transcription; 0051789: response to protein stimulus; 0060603: mammary gland duct morphogenesis; 0071109: superior temporal gyrus development |

**Downregulated genes in the patient with AP-1 consensus sequence**

| **Probe Set ID** | **Gene Symbol** | **Gene Ontology Biological Process** |
| --- | --- | --- |
| 33442_at | PRUNE2 | 0006915: apoptosis; 0006917: induction of apoptosis; 0051318: G1 phase: inferred from expression pattern |
| 32531_at | GJA1 | 0001701: in utero embryonic development; 0001764: neuron migration; 0001947: heart looping; 0002070: epithelial cell maturation; 0006810: transport; 0006915: apoptosis; 0006936: muscle contraction; 0007154: cell communication; 0007267: cell-cell signaling; 0007507: heart development; 0007507: heart development; 0007512: adult heart development; 0008016: regulation of heart contraction; 0008285: negative regulation of cell proliferation; 0009268: response to pH; 0010232: vascular transport; 0010628: positive regulation of gene expression; 0010629: negative regulation of gene expression; 0015867: ATP transport; 0016044: cellular membrane organization; 0016264: gap junction assembly; 0035050: embryonic heart tube development; 0043123: positive regulation of I-kappaB kinase/NF-kappaB cascade; 0043403: skeletal muscle tissue regeneration; 0043434: response to peptide hormone stimulus; 0045732: positive regulation of protein catabolic process; 0045844: positive regulation of striated muscle tissue development; 0048514: blood vessel morphogenesis; 0048812: neuron projection morphogenesis; 0051259: protein oligomerization; 0051924: regulation of calcium ion transport; 0060174: limb bud formation |
| 36892_at | ITGA7 | 0007155: cell adhesion; 0007160: cell-matrix adhesion; 0007229: integrin-mediated signaling pathway; 0007517: muscle organ development; 0008360: regulation of cell shape; 0048514: blood vessel morphogenesis |
| 40422_at | IGFBP2 | 0007026: negative regulation of microtubule depolymerization; 0010976: positive regulation of neuron projection development; 0010977: negative regulation of neuron projection development; 0023034: intracellular signaling pathway; 0030182: neuron differentiation; 0031115: negative regulation of microtubule polymerization; 0031117: positive regulation of microtubule depolymerization |
| 38026_at | FBLN1 | 0044419: interspecies interaction between organisms |
| 115_at | THBS1 | 0000187: activation of MAPK activity; 0001666: response to hypoxia; 0001937: negative regulation of endothelial cell proliferation; 0001953: negative regulation of cell-matrix adhesion; 0002040: sprouting angiogenesis; 0002544: chronic inflammatory response; 0002581: negative regulation of antigen processing and presentation of peptide or polysaccharide antigen via MHC class II; 0002605: negative regulation of dendritic cell antigen processing and presentation; 0003417: growth plate cartilage development; 0006915: apoptosis; 0006916: anti-apoptosis; 0006917: induction of apoptosis; 0006954: inflammatory response; 0006955: immune response; 0007050: cell cycle arrest; 0007155: cell adhesion; 0009749: response to glucose stimulus; 0010595: positive regulation of endothelial cell migration; 0010596: negative regulation of endothelial cell migration; 0010670: positive regulation of oxygen and reactive oxygen species metabolic process; 0010748: negative regulation of plasma membrane long-chain fatty acid transport; 0010751: negative regulation of nitric oxide mediated signal transduction; 0010754: negative regulation of cGMP-mediated signaling; 0010757: negative regulation of plasminogen activation; 0010759: positive regulation of macrophage chemotaxis; 0010763: positive regulation of fibroblast migration; 0010811: positive regulation of cell-substrate adhesion; 0016477: cell migration; 0016525: negative regulation of angiogenesis; 0016525: negative regulation of angiogenesis; 0018149: peptide cross-linking; 0030194: positive regulation of blood coagulation; 0030335: positive regulation of cell migration; 0030511: positive regulation of transforming growth factor beta receptor signaling pathway; 0032026: response to magnesium ion; 0032570: response to progesterone stimulus; 0032695: negative regulation of interleukin-12 production; 0032914: positive regulation of transforming growth factor-beta1 production; 0034605: cellular response to heat; 0040037: negative regulation of fibroblast growth factor receptor signaling pathway; 0042327: positive regulation of phosphorylation; 0042493: response to drug; 0042535: positive regulation of tumor necrosis factor biosynthetic process; 0043032: positive regulation of macrophage activation; 0043066: negative regulation of apoptosis; 0043154: negative regulation of caspase activity; 0043536: positive regulation of blood vessel endothelial cell migration; 0043537: negative regulation of blood vessel endothelial cell migration; 0043652: engulfment of apoptotic cell; 0045727: positive regulation of translation; 0045766: positive regulation of angiogenesis; 0050921: positive regulation of chemotaxis; 0051592: response to calcium ion; 0051895: negative regulation of focal adhesion assembly; 0051897: positive regulation of protein kinase B signaling cascade; 0051918: negative regulation of fibrinolysis |
| 38750_at | NOTCH3 | 0006350: transcription; 0006355: regulation of transcription, DNA-dependent; 0007219: Notch signaling pathway; 0007275: multicellular organismal development; 0030154: cell differentiation; 0030900: forebrain development; 0042246: tissue regeneration; 0045449: regulation of transcription; 0045596: negative regulation of cell differentiation; 0045665: negative regulation of neuron differentiation; 0048663: neuron fate commitment; 0050793: regulation of developmental process |
| 38508_s_at | TNXA; TNXB | 0006350: transcription; 0006355: regulation of transcription, DNA-dependent; 0006629: lipid metabolic process; 0006631: fatty acid metabolic process; 0006641: triglyceride metabolic process; 0006986: response to unfolded protein; 0007155: cell adhesion; 0007160: cell-matrix adhesion; 0007165: signal transduction; 0016337: cell-cell adhesion; 0030036: actin cytoskeleton organization; 0030198: extracellular matrix organization; 0030199: collagen fibril organization; 0032963: collagen metabolic process; 0043206: fibril organization; 0043506: regulation of JUN kinase activity; 0045449: regulation of transcription; 0048251: elastic fiber assembly |
| 32242_at | CRYAB | 0001666: response to hypoxia; 0002088: lens development in camera-type eye; 0006006: glucose metabolic process; 0006457: protein folding; 0006800: oxygen and reactive oxygen species metabolic process; 0006916: anti-apoptosis; 0006936: muscle contraction; 0006950: response to stress; 0007021: tubulin complex assembly; 0007517: muscle organ development; 0007568: aging; 0009408: response to heat; 0010629: negative regulation of gene expression; 0030308: negative regulation of cell growth; 0031109: microtubule polymerization or depolymerization; 0032355: response to estradiol stimulus; 0032387: negative regulation of intracellular transport; 0042542: response to hydrogen peroxide; 0043010: camera-type eye development; 0043066: negative regulation of apoptosis; 0043154: negative regulation of caspase activity; 0051260: protein homooligomerization; 0051403: stress-activated MAPK cascade; 0060561: apoptosis involved in morphogenesis |
| 36513_at | MFAP5 | 0001527: microfibril; 0005576: extracellular region; 0005578: proteinaceous extracellular matrix |
| 1741_s_at | IGFBP2 | 0001558: regulation of cell growth; 0006950: response to stress; 0007165: signal transduction; 0007565: female pregnancy; 0007568: aging; 0007584: response to nutrient; 0009612: response to mechanical stimulus; 0010226: response to lithium ion; 0032355: response to estradiol stimulus; 0032526: response to retinoic acid; 0032870: cellular response to hormone stimulus; 0040008: regulation of growth; 0042493: response to drug; 0043567: regulation of insulin-like growth factor receptor signaling pathway; 0043567: regulation of insulin-like growth factor receptor signaling pathway; 0043627: response to estrogen stimulus; 0048545: response to steroid hormone stimulus; 0051384: response to glucocorticoid stimulus |
| 39101_at | MYH2 | 0006936: muscle contraction; 0006936: muscle contraction; 0030049: muscle filament sliding |
| 35289_at | RABGAP1 | 0007049: cell cycle; 0007165: signal transduction; 0007186: G-protein coupled receptor protein signaling pathway; 0032313: regulation of Rab GTPase activity |
| 32243_g_at | CRYAB | 0001666: response to hypoxia; 0002088: lens development in camera-type eye; 0006006: glucose metabolic process; 0006457: protein folding; 0006800: oxygen and reactive oxygen species metabolic process; 0006916: anti-apoptosis; 0006936: muscle contraction; 0006950: response to stress; 0007021: tubulin complex assembly; 0007517: muscle organ development; 0007568: aging; 0009408: response to heat; 0010629: negative regulation of gene expression; 0030308: negative regulation of cell growth; 0031109: microtubule polymerization or depolymerization; 0032355: response to estradiol stimulus; 0032387: negative regulation of intracellular transport; 0042542: response to hydrogen peroxide; 0043010: camera-type eye development; 0043066: negative regulation of apoptosis; 0043154: negative regulation of caspase activity; 0051260: protein homooligomerization; 0051403: stress-activated MAPK cascade; 0060561: apoptosis involved in morphogenesis |
| 37996_s_at | DMPK | 0002028: regulation of sodium ion transport; 0006464: protein modification process; 0006468: protein phosphorylation; 0008016: regulation of heart contraction; 0014853: regulation of excitatory postsynaptic membrane potential involved in skeletal muscle contraction |
| 36497_at | AHNAK2 | 0005634: nucleus |
| 39729_at | PRDX2 | 0000187: activation of MAPK activity; 0002536: respiratory burst involved in inflammatory response; 0006916: anti-apoptosis; 0006979: response to oxidative stress; 0010310: regulation of hydrogen peroxide metabolic process; 0010671: negative regulation of oxygen and reactive oxygen species metabolic process; 0031665: negative regulation of lipopolysaccharide-mediated signaling pathway; 0032088: negative regulation of NF-kappaB transcription factor activity; 0032496: response to lipopolysaccharide; 0042098: T cell proliferation; 0042743: hydrogen peroxide metabolic process; 0042744: hydrogen peroxide catabolic process; 0042981: regulation of apoptosis; 0045454: cell redox homeostasis; 0045581: negative regulation of T cell differentiation; 0048538: thymus development; 0048872: homeostasis of number of cells; 0055114: oxidation reduction |
| 33764_at | GABBR2 | 0007165: signal transduction; 0007186: G-protein coupled receptor protein signaling pathway; 0007194: negative regulation of adenylate cyclase activity; 0007214: gamma-aminobutyric acid signaling pathway; 0007268: synaptic transmission |
| 38087_s_at | S100A4 | 0001837: epithelial to mesenchymal transition; 0043123: positive regulation of I-kappaB kinase/NF-kappaB cascade |
| 35352_at | ARNT2 | 0001666: response to hypoxia; 0001701: in utero embryonic development; 0006350: transcription; 0006355: regulation of transcription, DNA-dependent; 0007165: signal transduction; 0007417: central nervous system development; 0008284: positive regulation of cell proliferation; 0032355: response to estradiol stimulus; 0043066: negative regulation of apoptosis; 0045449: regulation of transcription; 0045941: positive regulation of transcription; 0045944: positive regulation of transcription from RNA polymerase II promoter |
| 38800_at | STMN2 | 0007026: negative regulation of microtubule depolymerization; 0010976: positive regulation of neuron projection development; 0010977: negative regulation of neuron projection development; 0023034: intracellular signaling pathway; 0030182: neuron differentiation; 0031115: negative regulation of microtubule polymerization; 0031117: positive regulation of microtubule depolymerization |
| 40069_at | SVIL | 0007010: cytoskeleton organization; 0007519: skeletal muscle tissue development |
| 1597_at | GAS6 | 0007165: signal transduction; 0008283: cell proliferation; 0040008: regulation of growth |
| 1466_s_at | FGF7 | 0001541: ovarian follicle development; 0006656: phosphatidylcholine biosynthetic process; 0007165: signal transduction; 0008284: positive regulation of cell proliferation 0008543: fibroblast growth factor receptor signaling pathway; 0008544: epidermis development; 0008654: phospholipid biosynthetic process; 0009611: response to wounding; 0010463: mesenchymal cell proliferation; 0010838: positive regulation of keratinocyte proliferation; 0030324: lung development; 0031069: hair follicle morphogenesis; 0031532: actin cytoskeleton reorganization; 0034394: protein localization at cell surface; 0042060: wound healing; 0043129: surfactant homeostasis; 0050679: positive regulation of epithelial cell proliferation; 0050731: positive regulation of peptidyl-tyrosine phosphorylation; 0050918: positive chemotaxis; 0051549: positive regulation of keratinocyte migration; 0051781: positive regulation of cell division; 0060445: branching involved in salivary gland morphogenesis; 0060501: positive regulation of epithelial cell proliferation involved in lung morphogenesis; 0060665: regulation of branching involved in salivary gland morphogenesis by mesenchymal-epithelial signaling; 0061033: secretion by lung epithelial cell involved in lung growth |
| 36136_at | TP53I11 | 0006950: response to stress; 0008285: negative regulation of cell proliferation |
| 38268_at | SLC1A1 | 0006810: transport; 0006835: dicarboxylic acid transport; 0007268: synaptic transmission; 0015813: L-glutamate transport; 0051260: protein homooligomerization; 0051938: L-glutamate import; 0070779: D-aspartate import |
| 32612_at | GSN | 0006915: apoptosis; 0007568: aging; 0008154: actin polymerization or depolymerization; 0014003: oligodendrocyte development; 0016192: vesicle-mediated transport; 0030041: actin filament polymerization; 0030155: regulation of cell adhesion; 0042060: wound healing; 0042246: tissue regeneration; 0045471: response to ethanol; 0046686: response to cadmium ion; 0048015: phosphoinositide-mediated signaling; 0051014: actin filament severing; 0051016: barbed-end actin filament capping; 0051593: response to folic acid; 0051693: actin filament capping |
| 1005_at | DUSP1 | 0006470: protein dephosphorylation; 0006950: response to stress; 0006979: response to oxidative stress; 0007049: cell cycle; 0009416: response to light stimulus; 0010033: response to organic substance; 0016311: dephosphorylation; 0032355: response to estradiol stimulus; 0032526: response to retinoic acid; 0032870: cellular response to hormone stimulus; 0033574: response to testosterone stimulus; 0042542: response to hydrogen peroxide; 0043065: positive regulation of apoptosis; 0045768: positive regulation of anti-apoptosis; 0051384: response to glucocorticoid stimulus; 0051591: response to cAMP; 0051592: response to calcium ion |
| 692_s_at | SOD3 | 0001666: response to hypoxia; 0006801: superoxide metabolic process; 0006950: response to stress; 0019430: removal of superoxide radicals; 0046688: response to copper ion; 0055114: oxidation reduction |
| 33410_at | ITGA6 | 0007044: cell-substrate junction assembly; 0007155: cell adhesion; 0007160: cell-matrix adhesion; 0007229: integrin-mediated signaling pathway; 0016337: cell-cell adhesion; 0022409: positive regulation of cell-cell adhesion; 0031581: hemidesmosome assembly; 0031668: cellular response to extracellular stimulus; 0033627: cell adhesion mediated by integrin; 0042475: odontogenesis of dentine-containing tooth; 0046847: filopodium assembly; 0050873: brown fat cell differentiation; 0050900: leukocyte migration |
| 33802_at | HMOX1 | 0001525: angiogenesis; 0001666: response to hypoxia; 0001935: endothelial cell proliferation; 0002246: wound healing involved in inflammatory response; 0002686: negative regulation of leukocyte migration; 0006788: heme oxidation; 0006916: anti-apoptosis; 0006979: response to oxidative stress; 0007243: intracellular protein kinase cascade; 0007264: small GTPase mediated signal transduction; 0007588: excretion; 0008217: regulation of blood pressure; 0008219: cell death; 0008285: negative regulation of cell proliferation; 0008630: DNA damage response, signal transduction resulting in induction of apoptosis; 0014806: smooth muscle hyperplasia; 0031670: cellular response to nutrient; 0032764: negative regulation of mast cell cytokine production; 0034101: erythrocyte homeostasis; 0034383: low-density lipoprotein particle clearance; 0035094: response to nicotine; 0042167: heme catabolic process; 0042542: response to hydrogen peroxide; 0043123: positive regulation of I-kappaB kinase/NF-kappaB cascade; 0043305: negative regulation of mast cell degranulation; 0043392: negative regulation of DNA binding; 0043433: negative regulation of transcription factor activity; 0043619: regulation of transcription from RNA polymerase II promoter in response to oxidative stress; 0043627: response to estrogen stimulus; 0045080: positive regulation of chemokine biosynthetic process; 0045765: regulation of angiogenesis; 0045768: positive regulation of anti-apoptosis; 0045909: positive regulation of vasodilation; 0048661: positive regulation of smooth muscle cell proliferation; 0048662: negative regulation of smooth muscle cell proliferation; 0050896: response to stimulus; 0051090: regulation of transcription factor activity; 0051260: protein homooligomerization; 0055072: iron ion homeostasis; 0055114: oxidation reduction |
| 39754_at | ITGB5 | 0006936: muscle contraction; 0007155: cell adhesion; 0007160: cell-matrix adhesion; 0007229: integrin-mediated signaling pathway; 0007275: multicellular organismal development |
| 39395_at | THY1 | 0001525: angiogenesis; 0006469: negative regulation of protein kinase activity; 0007010: cytoskeleton organization; 0016337: cell-cell adhesion; 0030336: negative regulation of cell migration; 0043547: positive regulation of GTPase activity; 0046549: retinal cone cell development; 0048041: focal adhesion assembly; 0050771: negative regulation of axonogenesis; 0050852: T cell receptor signaling pathway; 0050860: negative regulation of T cell receptor signaling pathway; 0050870: positive regulation of T cell activation; 0051281: positive regulation of release of sequestered calcium ion into cytosol |
| 36070_at | KIAA1199 | 0005737: cytoplasm |
| 973_at | SGK1 | 0006468: protein phosphorylation; 0006814: sodium ion transport; 0006915: apoptosis; 0006950: response to stress; 0006974: response to DNA damage stimulus |
| 37765_at | LMOD1 | 0006936: muscle contraction |
| 32667_at | COL4A5 | 0005576: extracellular region; 0005578: proteinaceous extracellular matrix; 0005581: collagen; 0005587: collagen type IV; 0005604: basement membrane |
| 35717_at | ABCA8 | 0006810: transport; 0006810: transport |
| 2058_s_at | ITGB5 | 0006936: muscle contraction; 0007155: cell adhesion; 0007160: cell-matrix adhesion; 0007229: integrin-mediated signaling pathway; 0007275: multicellular organismal development |
| 40455_at | ENDOD1 | 0005576: extracellular region |
| 39930_at | EPHB6 | 0006468: protein phosphorylation; 0007169: transmembrane receptor protein tyrosine kinase signaling pathway |
| 38267_at | SLC1A1 | 0006810: transport; 0006835: dicarboxylic acid transport; 0007268: synaptic transmission; 0015813: L-glutamate transport; 0051260: protein homooligomerization; 0051938: L-glutamate import; 0070779: D-aspartate import |
| 672_at | SERPINE1 | 0000302: response to reactive oxygen species; 0001300: chronological cell aging; 0010469: regulation of receptor activity; 0010757: negative regulation of plasminogen activation; 0010951: negative regulation of endopeptidase activity; 0014912: negative regulation of smooth muscle cell migration; 0030194: positive regulation of blood coagulation; 0030195: negative regulation of blood coagulation; 0030336: negative regulation of cell migration; 0032757: positive regulation of interleukin-8 production; 0033629: negative regulation of cell adhesion mediated by integrin; 0034097: response to cytokine stimulus; 0035491: positive regulation of leukotriene production involved in inflammatory response; 0042060: wound healing; 0042127: regulation of cell proliferation; 0042246: tissue regeneration; 0042593: glucose homeostasis; 0043066: negative regulation of apoptosis; 0045765: regulation of angiogenesis; 0045766: positive regulation of angiogenesis; 0048260: positive regulation of receptor-mediated endocytosis; 0050729: positive regulation of inflammatory response; 0050820: positive regulation of coagulation; 0050829: defense response to Gram-negative bacterium; 0051384: response to glucocorticoid stimulus; 0051918: negative regulation of fibrinolysis; 0061044: negative regulation of vascular wound healing; 0061045: negative regulation of wound healing; 0070887: cellular response to chemical stimulus; 0071222: cellular response to lipopolysaccharide; 0090026: positive regulation of monocyte chemotaxis |
| 40193_at | ENO2 | 0006094: gluconeogenesis; 0006096: glycolysis; 0014070: response to organic cyclic substance; 0032355: response to estradiol stimulus; 0042493: response to drug |
| 34836_at | RAB5C | 0006810: transport; 0007032: endosome organization; 0007264: small GTPase mediated signal transduction; 0015031: protein transport; 0030100: regulation of endocytosis |
| 37621_at | IL6ST | 0002675: positive regulation of acute inflammatory response; 0002821: positive regulation of adaptive immune response; 0005977: glycogen metabolic process; 0006642: triglyceride mobilization; 0007165: signal transduction; 0007259: JAK-STAT cascade; 0007584: response to nutrient; 0008284: positive regulation of cell proliferation; 0008593: regulation of Notch signaling pathway; 0010575: positive regulation vascular endothelial growth factor production; 0010613: positive regulation of cardiac muscle hypertrophy; 0014911: positive regulation of smooth muscle cell migration; 0019221: cytokine-mediated signaling pathway; 0034097: response to cytokine stimulus; 0042102: positive regulation of T cell proliferation; 0042511: positive regulation of tyrosine phosphorylation of Stat1 protein; 0042517: positive regulation of tyrosine phosphorylation of Stat3 protein; 0045669: positive regulation of osteoblast differentiation; 0045768: positive regulation of anti-apoptosis; 0048711: positive regulation of astrocyte differentiation; 0048861: leukemia inhibitory factor signaling pathway; 0051481: reduction of cytosolic calcium ion concentration; 0070104: negative regulation of interleukin-6-mediated signaling pathway; 0070106: interleukin-27-mediated signaling pathway; 0070120: ciliary neurotrophic factor-mediated signaling pathway |
| 36496_at | IMPA2 | 0001666: response to hypoxia; 0006801: superoxide metabolic process; 0006950: response to stress; 0019430: removal of superoxide radicals; 0046688: response to copper ion; 0055114: oxidation reduction |
| 36454_at | CA12 | 0006730: one-carbon metabolic process |
| 1495_at | LTBP1 | 0035583: negative regulation of transforming growth factor beta receptor signaling pathway by extracellular sequestering of TGFbeta |
| 1220_g_at | IRF2 | 0000122: negative regulation of transcription from RNA polymerase II promoter; 0006350: transcription; 0006355: regulation of transcription, DNA-dependent; 0008283: cell proliferation; 0045449: regulation of transcription; 0045449: regulation of transcription |
| 39593_at | FGL2 | 0007165: signal transduction |
| 1530_g_at | FRY | 0006350: transcription; 0045449: regulation of transcription |
| 34265_at | SCG5 | 0006810: transport; 0006886: intracellular protein transport; 0007218: neuropeptide signaling pathway; 0016486: peptide hormone processing; 0046883: regulation of hormone secretion |
| 37542_at | LHFPL2 | 0016020: membrane; 0016021: integral to membrane |
| 33878_at | EFHD1 | 0031175: neuron projection development |
| 33088_at | KCNH1 | 0000160: two-component signal transduction system (phosphorelay); 0006355: regulation of transcription, DNA-dependent; 0006810: transport; 0006811: ion transport; 0006813: potassium ion transport; 0006813: potassium ion transport; 0007165: signal transduction; 0007520: myoblast fusion; 0055085: transmembrane transport |
| 31983_at | EMX2 | 0006355: regulation of transcription, DNA-dependent; 0007275: multicellular organismal development; 0007420: brain development; 0009952: anterior/posterior pattern formation; 0010468: regulation of gene expression; 0021542: dentate gyrus development; 0021796: cerebral cortex regionalization; 0021846: cell proliferation in forebrain; 0021885: forebrain cell migration; 0030182: neuron differentiation; 0030900: forebrain development; 0042493: response to drug; 0045449: regulation of transcription; 0071109: superior temporal gyrus development |
| 32313_at | TPM2 | 0006936: muscle contraction; 0043462: regulation of ATPase activity |
| 39616_at | PTGER3 | 0006351: transcription, DNA-dependent; 0007165: signal transduction; 0007186: G-protein coupled receptor protein signaling pathway; 0008219: cell death; 0031622: positive regulation of fever |
| 38127_at | SDC1 | 0001657: ureteric bud development; 0009636: response to toxin; 0010033: response to organic substance; 0042060: wound healing; 0042476: odontogenesis; 0042542: response to hydrogen peroxide; 0048627: myoblast development; 0051384: response to glucocorticoid stimulus; 0051591: response to cAMP; 0051592: response to calcium ion; 0055002: striated muscle cell development; 0060009: Sertoli cell development; 0060070: canonical Wnt receptor signaling pathway |
| 36644_at | CD151 | 0007155: cell adhesion; 0031581: hemidesmosome assembly |
| 422_s_at | MAX | 0006350: transcription; 0006355: regulation of transcription, DNA-dependent; 0006366: transcription from RNA polymerase II promoter; 0006461: protein complex assembly; 0009267: cellular response to starvation; 0010243: response to organic nitrogen; 0032868: response to insulin stimulus; 0045449: regulation of transcription; 0048678: response to axon injury; 0051402: neuron apoptosis; 0060041: retina development in camera-type eye; 0071375: cellular response to peptide hormone stimulus |
| 32314_g_at | TPM2 | 0006936: muscle contraction; 0043462: regulation of ATPase activity |
| 36042_at | NTRK2 | 0001570: vasculogenesis; 0006468: protein phosphorylation; 0007169: transmembrane receptor protein tyrosine kinase signaling pathway; 0007190: activation of adenylate cyclase activity; 0007275: multicellular organismal development; 0007399: nervous system development; 0007631: feeding behavior; 0014047: glutamate secretion; 0019222: regulation of metabolic process; 0030154: cell differentiation; 0031547: brain-derived neurotrophic factor receptor signaling pathway; 0042490: mechanoreceptor differentiation; 0046548: retinal rod cell development; 0046777: protein autophosphorylation; 0046928: regulation of neurotransmitter secretion; 0050773: regulation of dendrite development; 0051968: positive regulation of synaptic transmission, glutamatergic |
| 32740_at | RAB11FIP2 | 0006810: transport; 0015031: protein transport |
| 36728_at | ADRA1D | 0001986: negative regulation of the force of heart contraction involved in baroreceptor response to increased systemic arterial blood pressure; 0001994: norepinephrine-epinephrine vasoconstriction involved in regulation of systemic arterial blood pressure; 0006259: DNA metabolic process; 0007165: signal transduction; 0007186: G-protein coupled receptor protein signaling pathway; 0007188: G-protein signaling, coupled to cAMP nucleotide second messenger; 0007267: cell-cell signaling; 0007275: multicellular organismal development; 0008283: cell proliferation; 0008284: positive regulation of cell proliferation |
| 34866_at | LOC151162; MGAT5 | 0006487: protein N-linked glycosylation; 0006487: protein N-linked glycosylation |
| 37641_at | IFI44 | 0009615: response to virus |
| 39422_at | PDE4DIP | 0005634: nucleus; 0005737: cytoplasm; 0005794: Golgi apparatus; 0005815: microtubule organizing center; 0005856: cytoskeleton |
| 31856_at | LRRC32 | 0005887: integral to plasma membrane; 0016020: membrane; 0016021: integral to membrane |
| 39058_at | ABR | 0006915: apoptosis; 0007165: signal transduction; 0007264: small GTPase mediated signal transduction; 0008624: induction of apoptosis by extracellular signals; 0035023: regulation of Rho protein signal transduction |
| 885_g_at | ITGA3 | 0001764: neuron migration; 0007155: cell adhesion; 0007160: cell-matrix adhesion; 0007229: integrin-mediated signaling pathway; 0007613: memory |
| 35871_s_at | SLC4A4 | 0006810: transport; 0006811: ion transport; 0006814: sodium ion transport; 0006820: anion transport |

**Downregulated genes in the patient with AP-1 consensus sequence**

| **Probe Set ID** | **Gene Symbol** | **Gene Ontology Biological Process** |
| --- | --- | --- |
| 39206_s_at | ACAN | 0001501: skeletal system development; 0001502: cartilage condensation; 0002063: chondrocyte development; 0006508: proteolysis; 0007155: cell adhesion; 0030166: proteoglycan biosynthetic process; 0030199: collagen fibril organization |
| 38965_at | ACAN | 0001501: skeletal system development; 0001502: cartilage condensation; 0002063: chondrocyte development; 0006508: proteolysis; 0007155: cell adhesion; 0030166: proteoglycan biosynthetic process; 0030199: collagen fibril organization |
| 39207_r_at | ACAN | 0001501: skeletal system development; 0001502: cartilage condensation; 0002063: chondrocyte development; 0006508: proteolysis; 0007155: cell adhesion; 0030166: proteoglycan biosynthetic process; 0030199: collagen fibril organization |
| 38965_at | ACAN | 0001501: skeletal system development; 0001502: cartilage condensation; 0002063: chondrocyte development; 0006508: proteolysis; 0007155: cell adhesion; 0030166: proteoglycan biosynthetic process; 0030199: collagen fibril organization |
| 37892_at | COL11A1 | 0001502: cartilage condensation; 0001503: ossification; 0002063: chondrocyte development; 0003007: heart morphogenesis; 0006029: proteoglycan metabolic process; 0007155: cell adhesion; 0007601: visual perception; 0007605: sensory perception of sound; 0030198: extracellular matrix organization; 0030199: collagen fibril organization; 0042472: inner ear morphogenesis; 0048704: embryonic skeletal system morphogenesis; 0048705: skeletal system morphogenesis; 0050910: detection of mechanical stimulus involved in sensory perception of sound; 0051216: cartilage development; 0055010: ventricular cardiac muscle tissue morphogenesis |
| 35303_at | INSIG1 | 0001558: regulation of cell growth; 0006950: response to stress; 0007165: signal transduction; 0007565: female pregnancy; 0007568: aging; 0007584: response to nutrient; 0009612: response to mechanical stimulus; 0010226: response to lithium ion; 0032355: response to estradiol stimulus; 0032526: response to retinoic acid; 0032870: cellular response to hormone stimulus; 0040008: regulation of growth; 0042493: response to drug; 0043567: regulation of insulin-like growth factor receptor signaling pathway; 0043627: response to estrogen stimulus; 0048545: response to steroid hormone stimulus; 0051384: response to glucocorticoid stimulus |
| 34203_at | CNN1 | 0006940: regulation of smooth muscle contraction; 0031032: actomyosin structure organization |
| 34403_at | MFGE8 | 0001525: angiogenesis; 0006910: phagocytosis, recognition; 0006911: phagocytosis, engulfment; 0007155: cell adhesion; 0007155: cell adhesion; 0007338: single fertilization; 0008284: positive regulation of cell proliferation; 0043627: response to estrogen stimulus; 0044419: interspecies interaction between organisms; 0050766: positive regulation of phagocytosis |
| 37399_at | AKR1C3 | 0006693: prostaglandin metabolic process; 0055114: oxidation reduction; 0055114: oxidation reduction |
| 32814_at | IFIT1 | 0005737: cytoplasm |
| 32521_at | SFRP1 | 0001649: osteoblast differentiation; 0001657: ureteric bud development; 0001756: somitogenesis; 0001954: positive regulation of cell-matrix adhesion; 0002244: hemopoietic progenitor cell differentiation; 0006309: DNA fragmentation involved in apoptotic nuclear change; 0007275: multicellular organismal development; 0008284: positive regulation of cell proliferation; 0008285: negative regulation of cell proliferation; 0009267: cellular response to starvation; 0009950: dorsal/ventral axis specification; 0009952: anterior/posterior pattern formation; 0010564: regulation of cell cycle process; 0010629: negative regulation of gene expression; 0010719: negative regulation of epithelial to mesenchymal transition; 0014070: response to organic cyclic substance; 0016055: Wnt receptor signaling pathway; 0016481: negative regulation of transcription; 0022601: menstrual cycle phase; 0030154: cell differentiation; 0030177: positive regulation of Wnt receptor signaling pathway; 0030178: negative regulation of Wnt receptor signaling pathway; 0030278: regulation of ossification; 0030279: negative regulation of ossification; 0030307: positive regulation of cell growth; 0030308: negative regulation of cell growth; 0030336: negative regulation of cell migration; 0032582: negative regulation of gene-specific transcription; 0032855: positive regulation of Rac GTPase activity; 0033689: negative regulation of osteoblast proliferation; 0042493: response to drug; 0043065: positive regulation of apoptosis; 0043066: negative regulation of apoptosis; 0043193: positive regulation of gene-specific transcription; 0043508: negative regulation of JUN kinase activity; 0044344: cellular response to fibroblast growth factor stimulus; 0045578: negative regulation of B cell differentiation; 0045600: positive regulation of fat cell differentiation; 0045765: regulation of angiogenesis; 0045880: positive regulation of smoothened signaling pathway; 0045941: positive regulation of transcription; 0046676: negative regulation of insulin secretion; 0046851: negative regulation of bone remodeling; 0048147: negative regulation of fibroblast proliferation; 0050679: positive regulation of epithelial cell proliferation; 0050680: negative regulation of epithelial cell proliferation; 0050732: negative regulation of peptidyl-tyrosine phosphorylation; 0051496: positive regulation of stress fiber assembly; 0051894: positive regulation of focal adhesion assembly; 0060070: canonical Wnt receptor signaling pathway; 0060071: Wnt receptor signaling pathway, planar cell polarity pathway; 0060218: hemopoietic stem cell differentiation; 0060527: prostate epithelial cord arborization involved in prostate glandular acinus morphogenesis; 0060687: regulation of branching involved in prostate gland morphogenesis; 0060766: negative regulation of androgen receptor signaling pathway; 0071305: cellular response to vitamin D; 0071347: cellular response to interleukin-1; 0071356: cellular response to tumor necrosis factor; 0071363: cellular response to growth factor stimulus; 0071380: cellular response to prostaglandin E stimulus; 0071391: cellular response to estrogen stimulus; 0071392: cellular response to estradiol stimulus; 0071456: cellular response to hypoxia; 0071504: cellular response to heparin; 0071560: cellular response to transforming growth factor beta stimulus; 0071773: cellular response to BMP stimulus; 0090090: negative regulation of canonical Wnt receptor signaling pathway; 0090175: regulation of establishment of planar polarity; 0090263: positive regulation of canonical Wnt receptor signaling pathway; 2000052: positive regulation of non-canonical Wnt receptor signaling pathway; 2000054: negative regulation of Wnt receptor signaling pathway involved in dorsal/ventral axis specification; 2000080: negative regulation of canonical Wnt receptor signaling pathway involved in controlling pancreatic B cell proliferation; 2000117: negative regulation of cysteine-type endopeptidase activity |
| 915_at | IFIT1 | 0005737: cytoplasm |
| 37014_at | MX1 | 0006917: induction of apoptosis; 0006952: defense response; 0007165: signal transduction; 0009615: response to virus |
| 37512_at | HSD17B6 | 0006629: lipid metabolic process; 0006702: androgen biosynthetic process; 0006710: androgen catabolic process; 0008152: metabolic process; 0008202: steroid metabolic process; 0055114: oxidation reduction |
| 875_g_at | CCL2 | 0006468: protein phosphorylation; 0006814: sodium ion transport; 0006915: apoptosis; 0006950: response to stress; 0006974: response to DNA damage stimulus |
| 37399_at | AKR1C3 | 0006693: prostaglandin metabolic process; 0055114: oxidation reduction |
| 434_at | H1F0 | 0006334: nucleosome assembly |
| 38446_at | XIST | na |
| 353_at | PITPNB | 0001701: in utero embryonic development; 0006629: lipid metabolic process; 0006810: transport |
| 1197_at | ACTG2 | 0006936: muscle contraction; 0008217: regulation of blood pressure; 0014829: vascular smooth muscle contraction |
| 2062_at | IGFBP7 | 0001558: regulation of cell growth; 0007155: cell adhesion; 0007566: embryo implantation; 0008285: negative regulation of cell proliferation; 0009408: response to heat; 0014070: response to organic cyclic substance; 0032526: response to retinoic acid; 0032870: cellular response to hormone stimulus; 0050810: regulation of steroid biosynthetic process; 0051414: response to cortisol stimulus |
| 31557_at | TMSB4X | 0007010: cytoskeleton organization; 0030036: actin cytoskeleton organization; 0042989: sequestering of actin monomers |
| 1221_at | RAB5B | 0006810: transport; 0007032: endosome organization; 0007264: small GTPase mediated signal transduction; 0015031: protein transport; 0030100: regulation of endocytosis |
| 39207_r_at | ACAN | 0001501: skeletal system development; 0001502: cartilage condensation; 0002063: chondrocyte development; 0006508: proteolysis; 0007155: cell adhesion; 0030166: proteoglycan biosynthetic process; 0030199: collagen fibril organization |
| 32675_at | BST1 | 0006959: humoral immune response; 0007275: multicellular organismal development; 0008152: metabolic process |
| 34069_s_at | SS18; SSX2 | 0000226: microtubule cytoskeleton organization; 0000902: cell morphogenesis; 0006350: transcription; 0006355: regulation of transcription, DNA-dependent; 0007010: cytoskeleton organization; 0007243: intracellular protein kinase cascade; 0042493: response to drug; 0045449: regulation of transcription; 0045944: positive regulation of transcription from RNA polymerase II promoter; 0048013: ephrin receptor signaling pathway |
| 34797_at | PPAP2A | 0006629: lipid metabolic process; 0007205: activation of protein kinase C activity by G-protein coupled receptor protein signaling pathway; 0008285: negative regulation of cell proliferation; 0008354: germ cell migration; 0019216: regulation of lipid metabolic process; 0030518: steroid hormone receptor signaling pathway; 0030521: androgen receptor signaling pathway; 0046839: phospholipid dephosphorylation |
| 37678_at | BAMBI | 0007179: transforming growth factor beta receptor signaling pathway; 0008284: positive regulation of cell proliferation; 0008360: regulation of cell shape; 0010718: positive regulation of epithelial to mesenchymal transition; 0016477: cell migration; 0030512: negative regulation of transforming growth factor beta receptor signaling pathway; 0030512: negative regulation of transforming growth factor beta receptor signaling pathway; 0032092: positive regulation of protein binding; 0035413: positive regulation of catenin protein nuclear translocation; 0045893: positive regulation of transcription, DNA-dependent; 0090263: positive regulation of canonical Wnt receptor signaling pathway |
| 39695_at | CD55 | 0006958: complement activation, classical pathway; 0007204: elevation of cytosolic calcium ion concentration; 0045087: innate immune response; 0045730: respiratory burst |
| 920_at | NKTR | 0006457: protein folding |
| 38503_at | ALDH1B1 | 0005975: carbohydrate metabolic process; 0008152: metabolic process; 0055114: oxidation reduction |
| 39145_at | MYL9 | 0006936: muscle contraction; 0006937: regulation of muscle contraction |
| 35961_at | --- | na |
| 37512_at | HSD17B6 | 0006629: lipid metabolic process; 0006702: androgen biosynthetic process; 0006710: androgen catabolic process; 0008152: metabolic process; 0008202: steroid metabolic process; 0055114: oxidation reduction |
| 39170_at | CD59 | 0007166: cell surface receptor linked signaling pathway; 0007596: blood coagulation |
| 34375_at | CCL2 | 0001666: response to hypoxia; 0001938: positive regulation of endothelial cell proliferation; 0002548: monocyte chemotaxis; 0006468: protein phosphorylation; 0006874: cellular calcium ion homeostasis; 0006916: anti-apoptosis; 0006935: chemotaxis; 0006954: inflammatory response; 0006955: immune response; 0006959: humoral immune response; 0007155: cell adhesion; 0007165: signal transduction; 0007166: cell surface receptor linked signaling pathway; 0007179: transforming growth factor beta receptor signaling pathway; 0007186: G-protein coupled receptor protein signaling pathway; 0007187: G-protein signaling, coupled to cyclic nucleotide second messenger; 0007259: JAK-STAT cascade; 0009408: response to heat; 0009612: response to mechanical stimulus; 0009617: response to bacterium; 0009887: organ morphogenesis; 0010332: response to gamma radiation; 0014823: response to activity; 0019079: viral genome replication; 0019221: cytokine-mediated signaling pathway; 0030593: neutrophil chemotaxis; 0031100: organ regeneration; 0032496: response to lipopolysaccharide; 0032570: response to progesterone stimulus; 0033552: response to vitamin B3; 0042493: response to drug; 0043200: response to amino acid stimulus; 0045471: response to ethanol; 0046677: response to antibiotic; 0048010: vascular endothelial growth factor receptor signaling pathway; 0048246: macrophage chemotaxis; 0048247: lymphocyte chemotaxis; 0050806: positive regulation of synaptic transmission; 0051384: response to glucocorticoid stimulus; 0060137: maternal process involved in parturition; 0070098: chemokine-mediated signaling pathway |
| 34512_at | ADRA2C | 0000187: activation of MAPK activity; 0007165: signal transduction; 0007186: G-protein coupled receptor protein signaling pathway; 0007267: cell-cell signaling; 0010700: negative regulation of norepinephrine secretion; 0032148: activation of protein kinase B activity; 0032811: negative regulation of epinephrine secretion; 0043406: positive regulation of MAP kinase activity; 0045666: positive regulation of neuron differentiation |
| 34012_at | KRT34 | 0008544: epidermis development |
| 531_at | GLIPR1 | 0005576: extracellular region; 0016020: membrane; 0016021: integral to membrane |
| 35674_at | PADI2 | 0006464: protein modification process; 0018101: peptidyl-citrulline biosynthetic process from peptidyl-arginine |
| 40776_at | DES | 0006936: muscle contraction; 0007010: cytoskeleton organization; 0007275: multicellular organismal development; 0007369: gastrulation; 0008016: regulation of heart contraction |
